# Acupuncture for nausea and vomiting induced by highly emetogenic chemotherapy: a systematic review and meta-analysis

**DOI:** 10.3389/fneur.2025.1692411

**Published:** 2026-01-05

**Authors:** Run Lin, Zining Guo, Jiewen Zhang, Liying Wang, Lu Zhang, Xiaorong Tang, Wenhao Liu, Shaoyang Cui, Nenggui Xu

**Affiliations:** 1 South China Research Center for Acupuncture and Moxibustion, Medical College of Acu-Moxi and Rehabilitation, Guangzhou University of Chinese Medicine, Guangzhou, China; 2 Shenzhen Hospital (Fu Tian) of Guangzhou University of Chinese Medicine, Shenzhen, China

**Keywords:** acupuncture, cancer, nausea, vomit, meta-analysis

## Abstract

**Background:**

Acupuncture shows potential in treating nausea and vomiting (CINV) induced by highly emetogenic chemotherapy (HEC). However, the certainty of its efficacy evidence remains unclear, warranting a comprehensive evaluation.

**Method:**

Two independent reviewers systematically searched eight databases from inception to December 2024 to identify eligible randomized controlled trials (RCTs). Relevant data were extracted using a standardized form, and risk of bias was assessed using the Cochrane risk of bias tool version 2.0 (ROB 2.0). Meta-analysis was performed using R Studio 4.4 software. Subgroup analysis was conducted based on acupuncture type. Additionally, publication bias was detected using appropriate methods according to the heterogeneity of different outcomes, where appropriate. Finally, evidence quality was rated using the GRADE system.

**Result:**

A total of 58 RCTs were included in this meta-analysis. Rob 2.0 result indicated that most studies were at high risk of bias, with low methodological quality. For the primary outcome, acupuncture significantly improved the complete control rate during the overall (RR 1.54, 95% CI 1.36–1.75; *P* < 0.001; *I*^2^ = 36%) and the delayed phase (RR 1.56, 95% CI 1.32–1.86; *P* < 0.001; *I*^2^ = 0%). For other CINV outcomes, acupuncture demonstrated considerable therapeutic potential for vomiting-related outcomes, while uncertainty in alleviating nausea symptoms. Subgroup analyses showed that different acupuncture types had distinct advantages. Sensitivity analyses for several outcomes were unstable, and there were indications of publication bias. According to GRADE, only the acute vomiting duration score was rated as moderate quality; all other outcomes were rated as low or very low quality.

**Conclusion:**

Although acupuncture for HEC-induced CINV shows some positive effects, there are various limitations that render the current evidence insufficient to conclusively establish its efficacy; therefore, further high-quality studies are required.

**Systematic review registration:**

PROSPERO, identifier: CRD42024588165.

## Introduction

According to Global Cancer Observatory (GLOBOCAN) data, approximately 30 million new cancer cases and deaths were reported worldwide in 2022, with cancer remaining the “top threat” contributing to the global disease burden (1). Moreover, caner-induced public-health burden is projected to rise steadily over the next 20 years (2, 3). Chemotherapy, a cornerstone of cancer management, plays a pivotal role in improving patient prognosis (4, 5). However, its clinical benefits are frequently offset by treatment-related adverse events (AEs), among which chemotherapy-induced nausea and vomiting (CINV) is especially challenging (6–8). Approximately 60%−80% of patients receiving chemotherapy develop CINV. Even when chemotherapy is completed as planned, CINV continues to markedly diminish quality of life (9). More importantly, persistent CINV markedly undermines treatment adherence in cancer patients, preventing chemotherapy from achieving optimal therapeutic effects and thereby resulting in unavoidable disease progression and escalating healthcare costs. Additionally, CINV may lead to reduced or interrupted oral intake, initiating a progressive chain reaction—from malnutrition to impaired immunity and ultimately to treatment delays or interruptions (8, 10, 11). Accordingly, organizations such as the Multinational Association of Supportive Care in Cancer/European Society for Medical Oncology (MASCC/ESMO) and the National Comprehensive Cancer Network (NCCN) consistently regard how to manage CINV as a core issue of optimizing cancer treatment (6–8, 12).

Based on the chemotherapy regimen, CINV can be classified as highly emetogenic (HEC, risk >90%), moderately emetogenic (MEC, 30%−90%), low emetogenic (LEC, 10%−30%), or minimally emetogenic (<10%) (6–8). Most clinical practice guidelines align in their recommendations for CINV management, recommending triple standard therapy—dexamethasone (DEX), a 5-HT3 receptor antagonist, and an NK1 receptor antagonist—or add olanzapine to form a four-drug regimen (6, 7, 13). Although these regimens have achieved encouraging control of vomiting and perform well in non-HEC regimens, several limitations remain, including daytime somnolence, constipation, and headache. Recent studies have also raised concerns that the corticosteroid core component of standard therapy may diminish the efficacy of immune checkpoint inhibitors (CPIs), posing potential risks for patients undergoing combination immunotherapy (10, 14, 15). Notably, these limitations are most pronounced in HEC patients, 34% of whom fail to respond to standard prophylaxis (10, 13). In addition, adherence remains a challenge: a survey across five European countries found that only 15% of HEC patients received the standard therapy, indicating suboptimal compliance (16, 17). Crucially, failure to achieve adequate CINV control in the first chemotherapy cycle quadruples the risk of subsequent CINV episodes (10, 18). Overall, this evidence highlights critical gaps in current standard therapy and underscores the urgent need to explore safe, effective adjunctive treatments to address shortcomings, such as the unmet needs of HEC patients.

Complementary and Integrative Interventions (CIH) have experienced rapid growth in oncology over the past 20 years. In Canada, more than two-thirds of cancer survivors and healthcare professionals believe that CIH can improve cancer-related symptoms (8, 19). Acupuncture, because of its minimal side effects and demonstrated efficacy, has become one of the important CIH therapies. To date, acupuncture is practiced in over 103 countries worldwide; in the United States alone, approximately 3.5 million people receive acupuncture annually, and in China, its country of origin, the number is even higher (19, 20). On this basis, numerous studies have explored the efficacy of acupuncture in alleviating CINV and demonstrating promising results (21–24). Currently, NCCN, the Chinese Society of Clinical Oncology (CSCO), and MASCC/ESMO all recommend that acupuncture could be considered for CINV management when feasible (7, 13, 25). Moreover, a recent study produced encouraging evidence that electroacupuncture (EA), combined with standard therapy, increased the CINV complete control rate (no vomiting and no significant nausea) by nearly 20% and significantly reduced nausea scores (24). Notably, this study prioritized nausea as the primary endpoint and specifically enrolled patients receiving highly emetogenic chemotherapy, differing from previous research and opening new avenues for acupuncture studies in CINV.

However, previous summaries of evidence on acupuncture for CINV have paid insufficient attention to the HEC population and nausea symptoms. The quality of evidence regarding the efficacy of acupuncture for HEC-induced CINV remains unclear. Additionally, previous studies have not comprehensively organized outcome measures (26). Meanwhile, the types of chemotherapy drugs included in HEC vary across different regions. It is necessary to address these limitations and conduct a comprehensive review. Therefore, this study aims to comprehensively evaluate the efficacy and level of evidence of acupuncture treatment for CINV in HEC across different outcomes.

## Method

### Protocol and registration

This study was conducted and reported in accordance with the PRISMA 2020 statement for systematic reviews and meta-analyses and was registered in the International Prospective Register of Systematic Reviews (PROSPERO, registration number: CRD42024588165) (27).

### Inclusion criteria

Inclusion criteria were defined according to the Participants, Interventions, Comparisons, Outcomes, and Study design (PICOS) principle.

Participants (P): patients diagnosed with cancer and receiving HEC drug (studies with a title of HEC population without specifying chemotherapy drugs were not included); since different health organizations have varying standards for HEC, we comprehensively integrated HEC drug regimens specified by ASCO and CSCO to maximize the inclusion of studies, with specific information available in Supplementary material S1 (6, 28).

Interventions (I): Common types of acupuncture in the cancer field.

Control (C): C: Antiemetic with or without sham acupuncture.

Outcome (O): CINV-related outcome. We originally planned to use the complete protection rate (no vomiting, no significant nausea) as one of the primary outcomes of this study. However, due to the limitations of most studies having a large amount of discrete data and not clearly defining how to define significant nausea (lack of effective tool definition), we were unable to use the complete protection rate as one of the primary outcome. Despite this, after organizing the large amount of discrete data, we still included the absence of significant nausea as a separate secondary outcome. Therefore, the primary outcome of this study includes the complete control rate (no vomiting and nausea events) during the overall/acute phase (0–24 h)/delayed phase (Start of Treatment-End of Treatment).

Study (S): Randomized controlled trials (RCTs), without language restrictions. Detailed PICOS information is shown in Table 1.

**Table 1:** Inclusion criteria.

**PICO**	**Inclusion criteria**
Patient	①Diagnosed with cancer ②Intravenous injection of HEC for cancer treatment
Intervention	①Acupuncture interventions commonly used in oncology, including manual acupuncture (MA), electroacupuncture (EA), auricular acupuncture (AA), and transcutaneous acupoint electrical stimulation (TES) ②The intervention group received either acupuncture alone or acupuncture combined with no more than one additional treatment modality
Comparison	Antiemetic alone or antiemetic combined with sham acupuncture
Outcome	Primary outcome: complete control rate during the overall/acute/delayed phases, defined no vomiting and nausea events Second outcome: ①No significant nausea, no vomiting, and no CINV events during the overall/acute/delayed phases; ② Nausea-related scores: severity, duration, and frequency of nausea during the overall/acute/delayed phases; Vomiting-related scores: severity, duration, volume, and frequency of vomiting during the acute, delayed, and overall phases.
Study type and others	①Only RCTs are included ②Language is not restricted

### Exclusion criteria

(1) Animal experiments, systematic reviews or meta-analyses, scoping reviews, and other non-RCT studies. (2) Study comparing two different acupuncture types (both arms receive acupuncture) or those in which the intervention arm comprised more than two combined interventions.

### Study sources

Two independent reviewers (ZN.G and JW.Z), based on inclusion and exclusion criteria, searched eight databases, including PubMed, Embase, Cochrane Library, Web of Science (WOS), China National Knowledge Infrastructure (CNKI), WeiPu (VIP), WanFang, and Chinese biomedical literature service system (SinoMed), spanning from database inception to December 2024. The search strategy was consulted with a database search expert from Guangzhou University of Traditional Chinese Medicine, using medical subject headings (MeSH) and keyword combinations for retrieval, with specific MeSH terms, including “Acupuncture,” “Drug Therapy,” “Vomiting,” “Nausea,” “Randomized Controlled Trial.” The search strategy for RCTs was based on the strategy developed by a database librarian from Harvard University's Countway Library for the highest sensitivity and specificity in retrieving RCTs (29). All finalized search strategies were reviewed by a search expert from Guangzhou University of Traditional Chinese Medicine to ensure the maximum search scope and avoid missing studies. The search strategies were adjusted according to different databases. The search strategies are detailed in Supplementary Table S2.

### Study selection

Two independent reviewers (ZN.G and LY.W) imported the initial retrieval results into EndNote 20.0 (Clarivate Analytics Company, Philadelphia, PA), using the deduplication function to remove duplicate studies and further perform manual deduplication. Subsequently, a preliminary screening of titles and abstracts was conducted based on inclusion and exclusion criteria. For studies initially screened and meeting the inclusion criteria, full-text reading was performed to ensure complete compliance with the standards, ultimately including them in the analysis. In case of any disagreements during the process, the advice of a third reviewer (LY.W) was consulted to reach a consensus and resolve the issue. The reasons for excluding all studies will be recorded in Supplementary Table S3.

### Data extraction

Data extraction tables referred to the details of acupuncture interventions required by Standards for Reporting Interventions in Clinical Trials of Acupuncture (STRICTA) and combined with RCT-related characteristics were pre-designed and extracted using Microsoft Office Excel 2021 (30). Specifically, data extraction included general characteristics of the study, such as authors, publication year, country, cancer type, HEC drugs, and outcome measurement; intervention and control group characteristics included acupuncture type, control group type, acupuncture duration, and sessions. Additional supplementary information included the total sample size and whether ethical registration was conducted. For statistical data, quantitative data included extracting outcome-related mean ± standard deviation (MD ± SD) and the number of events. Furthermore, if the data in the study were not using MD ± SD, we converted it to MD ± SD using formulas from the Cochrane manual and Tong's approximation conversion method based on skewness (31, 32).

### Risk of bias

Two independent reviewers (ZNG and RL) independently assessed the methodological quality of the included RCTs using the Cochrane Bias Risk Assessment Tool (ROB 2.0) (100), with disagreements resolved through discussion with a third reviewer.

### Statistical analysis

Meta-analysis were merged using the “meta” package in R Studio 4.4. Effect sizes for continuous variables were expressed as MD or standardized mean difference (SMD) and 95% confidence interval (CI), based on the homogeneity of measurement tools between studies. Dichotomous data were represented using relative risk (RR) and 95% CI, with a significance level of 0.05. Notably, for three-arm studies, following the Cochrane Handbook recommendations, we independently compared the acupuncture intervention group with the other two groups and treated them as separate studies (31). Additionally, for sample sizes with an even number, we halved the sample size; for odd numbers, we used (*N* ± 1)/2 to avoid statistical power inflation (31). Heterogeneity size, indicating the relationship between meta-analysis results and random error, was quantified using the *I*^2^ statistic, where *I*^2^ <25% represents low heterogeneity, *I*^2^ between 25 and 50% indicates moderate heterogeneity, and *I*^2^ > 50% signifies high heterogeneity. Based on the heterogeneity size, data were merged using fixed-effect or random-effects models. When at least three studies reported for the outcome, we performed subgroup analyses according to acupuncture type to clarify the effects of different acupuncture types and to explore potential sources of heterogeneity. Sensitivity analyses were conducted using a leave-one-out approach to assess the robustness of the meta-analysis result. For outcomes with 10 or more studies, appropriate statistical methods were applied to evaluate the risk of publication bias, with the choice of method determined by the degree of heterogeneity (33).

## Result

### Study selection

**Figure 1:**
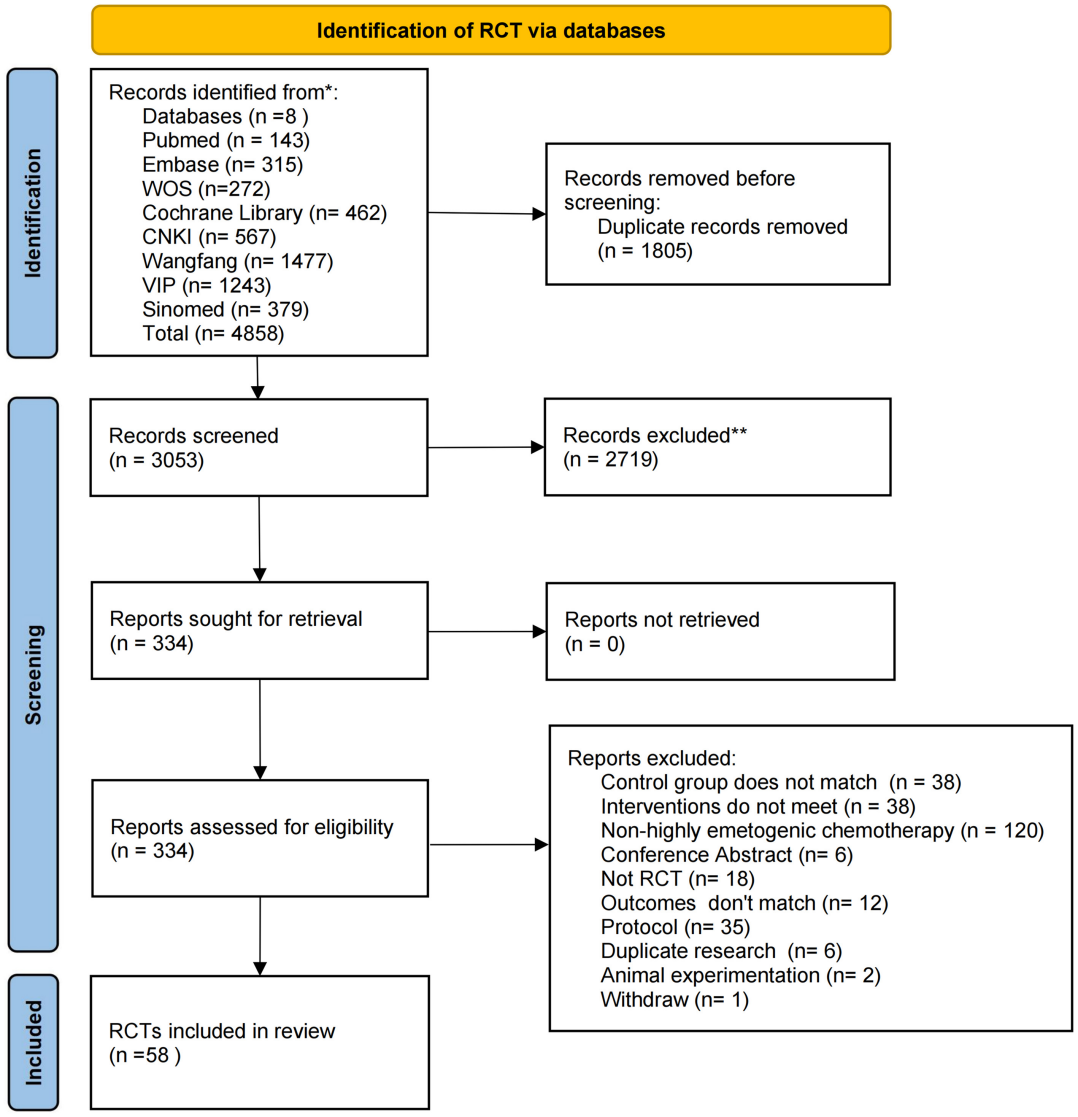
Flow chart.

Initially, 4,858 studies were retrieved, and 1,805 studies were removed using the deduplication function. Subsequently, two independent reviewers screened titles and abstracts based on inclusion and exclusion criteria, initially excluding 2,719 studies, leaving 334 studies. After full-text reading of the 334 studies, 276 were excluded for various reasons, with a final total of 58 studies included in the meta-analysis, as shown in the specific research process in Figure 1, and detailed information on excluded studies is provided in Supplementary Table S3 (24, 34–91).

### Study characteristics

The 58 studies were published between 2000 and 2024, involving a total of 4,685 participants (24, 34–91). Most studies were conducted in China, with other countries including Australia, South Korea, the United States, and Thailand. The studies covered various cancer types, such as colon, liver, breast, and lung cancer, though most studies did not limit participation by cancer type. In the intervention groups, MA was used in the majority of studies. Control groups used various antiemetics, with a small portion employing standard therapy. Outcome measures spanned multiple tools: binary outcomes measurement included the World Health Organization (WHO) antineoplastic adverse level and the number of CINV events, while continuous outcomes measurement was assessed using scales such as the Visual Analog Scale (VAS) and Index of Nausea, Vomiting, and Retching (INVR). Detailed information in Table 2 (24, 34–91).

**Table 2:** Study characteristics.

**Author**	**Year**	**Country**	**Cancer type**	**Chemotherapy drugs**	**Sample size**	**Acupuncture type**	**Control**	**Antiemetic**	**Existing time (Session)**	**Outcome**
Jane M. Beit	2012	Australia	Breast	AC/FEC	30	MA	Sham EA	5 HT_3_+NK_1_+Dexamethasone	20 min (4)	MASCC
Chi Hoon Maeng	2022	Korea	Solid	AC/Cisplatin	42	MA	Antiemetic	NK_1_+D_2_+5-HT_3_	20 min (3)	INVR; MASCC
Ting Mao	2021	China	Lung	Cisplatin-base	122	TES	Usual care	5 HT_3_+NK_1_+Dexamethasone	25 min (14)	MSAS
Kulthida Rithirangsriroj	2015	Thailand	Gynecologic	PC	70	MA	Antiemetic	5 HT_3_+Dexamethasone	30 min (2)	Events
Guoshuang Shen	2024	China	Anytype	EC/Carboplatin/Cisplatin-based	239	EA	Sham EA	5 HT_3_+NK_1_+Dexamethasone	30 min (3)	VAS
Joannie Shen	2000	USA	Breast	CPA/cDDP/BCNU	70	EA	Sham EA/Antiemetic	H_1_+BDZ+D_2_	20 min (5)	Events
Konrad Streitberger	2003	German	Anytype	Various combinations HEC	80	MA	Sham MA	H_1_+BDZ+D_2_	20 min (2)	Events
Jing Xie	2017	China	Liver	Cisplatin	142	TES	Sham TES	5 HT_3_	30 min (12)	Events; VAS
Xing Zhang	2014	China	Anytype	CPA/cDDP/BCNU	72	TES	Sham TES	5 HT_3_	60 min (3)	Events
Xiaoyan Fu	2024	China	Nasopharynx	PC	80	QA	Antiemetic	5 HT_3_	NA (5)	WHO
Shuzeng He	2024	China	Anytype	Cisplatin-based	70	MA	Antiemetic	5 HT_3_	55 min (3)	WHO; INVR
Sheng Wang	2017	China	Anytype	Cisplatin-based	171	MA	Antiemetic	5 HT_3_	30 min (5)	Events
Bei Yao	2024	China	Lung	AP/DP	68	QA+AP	Antiemetic	5 HT_3_+H_1_	24 h (3)	WHO; INVR
Qingyan Duan	2022	China	Anytype	Cisplatin-based	58	MA+AA	Antiemetic	5 HT_3_	30 min (5)	Events/VAS
Yun Yang	2019	China	Anytype	Cisplatin-based	58	MA	Antiemetic	5 HT_3_	30 min (7)	1990 ESMO
Erhua Yao	2017	China	Nasopharyngeal	Cisplatin-based+Radiotherapy	83	MA	Antiemetic	5 HT_3_	30 min (2)	CTCAE
Lingling Liu	2023	China	Ovarian	PC	100	MA+AP	Antiemetic	H_1_	NA	INVR; VAS;Events
Qiong Zhang	2012	China	Lung	Cisplatin-based	60	AA+MX	Antiemetic	5 HT_3_+Dexamethasone	NA (10)	NCI
Xuying Gu	2020	China	Colon	Cisplatin-based	120	MA	Antiemetic	5 HT_3_	30 min (3)	WHO
Xing Zhang	2014	China	Anytype	Various combinations HEC	72	MA	Sham MA	5 HT_3_	30 min (3)	Events
Dianrong Lu	2017	China	Anytype	Cisplatin-based	60	EA	Antiemetic	5 HT_3_	30 min (3)	Events
Kejiang Huang	2016	China	Breast	FAC	120	EA+AA	Antiemetic	5 HT_3_	30 min (16)	Events
Jianghua Yan	2017	China	Anytype	Cisplatin-based	60	EA	Antiemetic	5 HT_3_+Dexamethasone	30 min (4)	INVR
Shuixiu Yang	2021	China	Anytype	DDP+5-FU	60	MA	Antiemetic	5 HT_3_	NA (7)	WHO
Mengjun San	2020	China	Lung	AP/DP/TP	73	MA	Antiemetic	5 HT_3_	45 min (5)	WHO
Jie Tai	2009	China	Anytype	DDP	90	MA+AA	Antiemetic	5 HT_3_	30 min (6)	WHO
Lichun Liang	2018	China	Breast	EC	100	AA+AP	Antiemetic	5 HT_3_	30 min (2)	1990 ESMO
Aiyu Lu	2016	China	Breast	Cisplatin-based	80	AA	Antiemetic	5 HT_3_+Unclear	3 min (3)	WHO
Minzhu Sun	2017	China	Anytype	Cisplatin-based	68	AA+AP	Antiemetic	5 HT_3_+D_2_+Dexamethasone	NR (NR)	MASCC
Yinping Zhou	2018	China	Breast	EC	56	AA+AP	Antiemetic	5 HT_3_	NR (6)	Events
Ling Zhu	2014	China	Breast	Various combinations HEC	84	AA	Antiemetic	5 HT_3_	NR (12)	WHO
Xiaoling Wang	2017	China	Breast	AC-T	50	AA	Antiemetic	5 HT_3_+Dexamethasone	NR (NR)	WHO
Liming Zhang	2017	China	Anytype	Cisplatin-based	60	AA	Antiemetic	5 HT_3_	NR (NR)	1990 ESMO
Xueying Li	2020	China	Breast	EC	92	EA+AA	Antiemetic	5 HT_3_	20 min (12)	CTCAE
Zhuo Liu	2021	China	Anytype	Cisplatin-based	128	EA+AA	Antiemetic	5 HT_3_+Dexamethasone	30 min (3)	WHO; INVR
Yonghao Li	2014	China	Lung	DP	91	AA	Antiemetic	5 HT_3_	NR (7)	Events
Xiangqin Jiang	2012	China	Anytype	AP	85	AA	Antiemetic	5 HT_3_	NR (7)	WHO
Qiuhui Zheng	2021	China	Anytype	Cisplatin-based	64	MA+CHP	Antiemetic	5 HT_3_+Dexamethasone	30 min (6)	WHO; Events
Da Guo	2011	China	Bone	Cisplatin	52	MA	Antiemetic	5 HT_3_	30 min (4)	WHO
Wenhua Lin	2022	China	Ovarian	PC	100	AA+AP	Antiemetic	NR	NR (5)	MASCC
Xiaoxiao Liu	2019	China	Anytype	Cisplatin	60	TES+AA	Antiemetic	5 HT_3_+D_1_+Unclear	NR (NR)	Events
Yin Xu	2016	China	Anytype	Cisplatin-based	44	MA	Antiemetic	D_1_+H_2_	30 min (20)	Events
Donghua Li	2020	China	lung cancer	DP	64	MA	Antiemetic	5 HT_3_+D_1_+Dexamethasone	10 min (8)	CTCAE
Yinfeng Huang	2022	China	Esophageal	Cisplatin+5-FU	64	AA	Antiemetic	5 HT_3_+D_1_	NR	Unverified Tool
Chunyan Peng	2023	China	Lung	PC	75	QA	Antiemetic	5 HT_3_+Dexamethasone	NA (4)	INVR; Events
Chunyan Peng	2022	China	Lung	EP	65	QA	Antiemetic	5 HT_3_+NK_1_	NA (4)	INVR; Events
Siyu Zhao	2023	China	Breast	EC	82	MA+AA	Antiemetic	5 HT_3_	20 min (5)	CTCAE; Events
Haining Yang	2023	China	Gastric	DCF	161	MA	Antiemetic	5 HT_3_+Omeprazole	30 min	WHO; CTCAE
Huihuang Xiao	2022	China	Anytype	AC/GP/DP	40	MA	Antiemetic	5 HT_3_	30 min (5)	INVR; Events
Gu Cai	2019	China	Lung	EP	80	QA	Antiemetic	5 HT_3_+Dexamethasone	NR (4)	FLIE
Shuixiu Yang	2021	China	Anytype	DDP+5-FU	72	QA	Antiemetic	5 HT_3_+Dexamethasone	NR (NR)	WHO
Xingrong Xu	2020	China	Lung	EP/GP	100	MA	Antiemetic	5 HT_3_	30 min (7)	INVR; Events
Jiasheng Lin	2019	China	Anytype	Cisplatin-based	60	MA+MX	Antiemetic	5 HT_3_	20 min (7)	Events
Wen He	2012	China	Lung	EP	49	MA+AA	Antiemetic	5 HT_3_	30 min (3)	WHO; Events
Qing Zhang	2006	China	Anytype	Cisplatin	86	MA	Antiemetic	5 HT_3_+D_1_	30 min (3)	Events
Hongkang Lai	2011	China	Anytype	Cisplatin-based	60	MA	Antiemetic	5 HT_3_	30 min (6)	Unclear tool
Linghong Cai	2011	China	Lung	GP	80	MA	Antiemetic	5 HT_3_+NK_1_+Dexamethasone	30 min (NR)	1990 ESMO
Chunli Zhang	2014	China	Anytype	Cisplatin-based	63	MA	Antiemetic	5 HT_3_+D_1_	30 min (3)	WHO

AC, Anthracycline+Cyclophosphamide; AP, Doxorubicin+Cisplatin; AC-T, Anthracycline+Cyclophosphamide → Taxane; DP, Docetaxel+Cisplatin; DCF, Docetaxel+Cisplatin+5-Fluorouracil; CPA/cDDP/BCNU, Cyclophosphamide+Cisplatin+Carmustine; FAC, Fluorouracil+Doxorubicin+Cyclophosphamide; EC, Doxorubicin+Cyclophosphamide; EP, Etoposide + Cisplatin; GP, Gemcitabine+Cisplatin; PC, Paclitaxel+Cisplatin; PC, Paclitaxel+Cisplatin; 5 HT3, 5-hydroxytryptamine type 3 receptor antagonist; NK1, Neurokinin-1 receptor antagonist; H1, Histamine type 1 receptor antagonist; H2, Histamine type 2 receptor antagonist; D1, Dopamine D1 receptor antagonist; MASCC, MASCC Antiemesis Tool; INVR, Index of Nausea, Vomiting, and Retching; WHO, World Health Organization antineoplastic adverse level; CTCAE, Common Terminology Criteria for Adverse Events; VAS, Visual analog scale; ESMO, European Society For Medical Oncology Efficacy Assessment; FLIE, Functional living index-emesis; NR, Not report.

### Risk of bias

**Figure 2:**
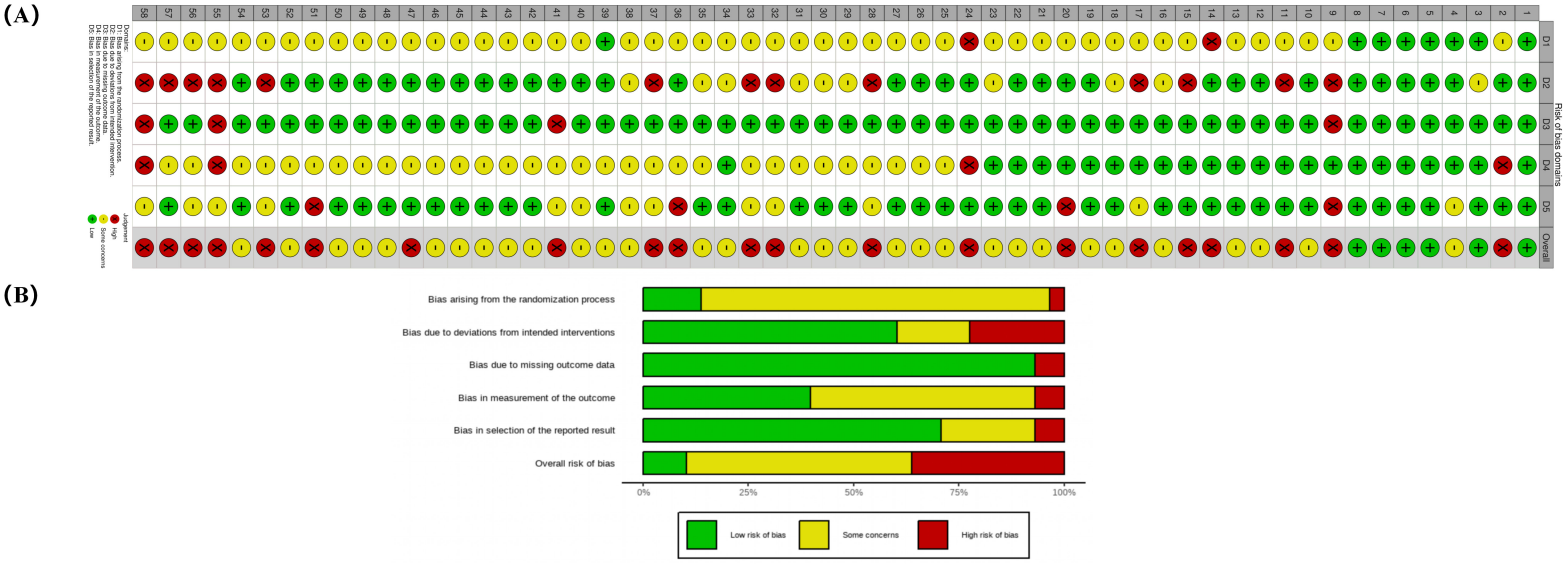
Summary risk of bias. **(A)** Risk of bias domains. **(B)** Individual risk of the bias.

The results of bias risk were not ideal, with 21 studies rated as “high bias” risk, 31 studies rated as “some concern,” and only six studies rated as “low bias” risk (24, 34–91). Specifically, in the randomization bias assessment, most studies were downgraded due to unclear reporting of randomization methods and allocation concealment. In terms of intervention bias related to blinding, most studies were downgraded in this domain because it is difficult to blind the acupuncturists. Additionally, in outcome measurement bias, some studies were downgraded due to unreported outcome assessor blinding and the use of unvalidated tools. Some studies also had selective reporting issues, leading to downgrades in reporting bias assessment. Specific bias risk information can be found in Figure 2 (24, 34–91).

### Meta-analysis of primary outcome

#### Overall complete control rate

**Figure 3:**
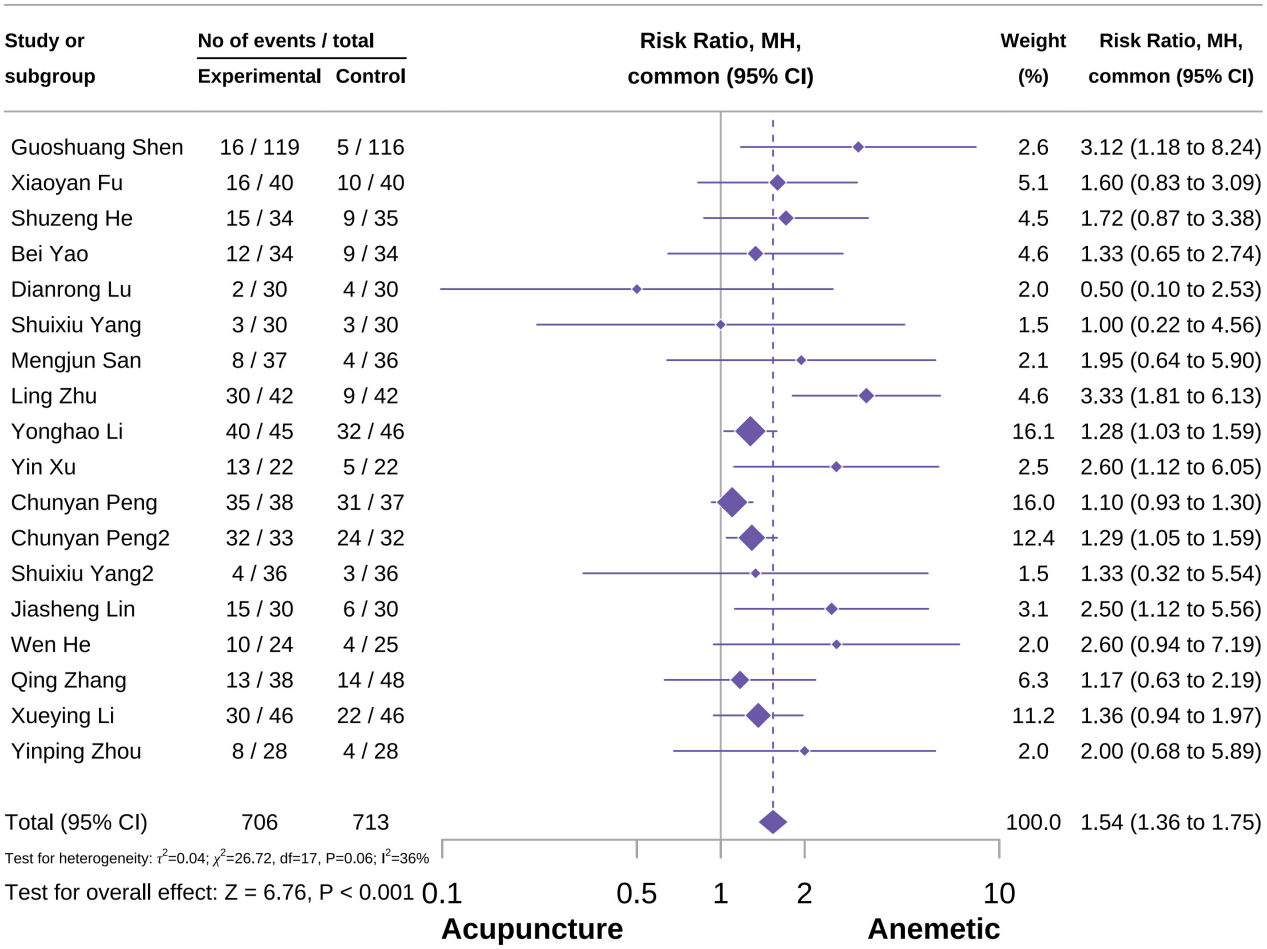
Forest plot of overall complete control rate.

The overall complete control rate included 18 studies, with a total sample size of 1,419 patients, including 706 patients in the acupuncture group and 713 patients in the control group (24, 41, 42, 44, 52, 55, 56, 60, 62, 63, 66, 68, 74, 75, 79, 86–89). Meta-analysis results showed that acupuncture could effectively improve the overall complete control rate of CINV compared to the control group [RR = 1.54, 95% CI = (1.36–1.75), *P* < 0.001], as shown in Figure 3. To further compare the effects of different acupuncture types and explore heterogeneity, we conducted a subgroup analysis. Results showed that EA, which demonstrated advantages in reducing HEC-induced CINV in recent studies, could not effectively improve the overall complete control rate [RR = 1.96, 95% CI = (0.91–4.25), *P* = 0.09, *I*^2^ = 72%]. The most commonly used MA showed significant advantages in the overall complete control rate with less inter-study variation [RR = 1.61, 95% CI = (1.12–2.32), *P* = 0.01, *I*^2^ = 0%]. Additionally, QA and AA also demonstrated certain effects in the overall complete control of CINV [RR = 1.25, 95% CI = (1.06–2.32), *P* = 0.007, *I*^2^ = 0%; RR = 1.73, 95% CI = (1.37–2.20), *P* < 0.001, *I*^2^ = 88%]. There were several scattered studies on acupuncture combined with different treatment measures, which could only display effect sizes. Sensitivity analysis results confirmed the reliability of the result, as shown in Supplementary Figures S1, S2.

#### Acute complete control rate

**Figure 4:**
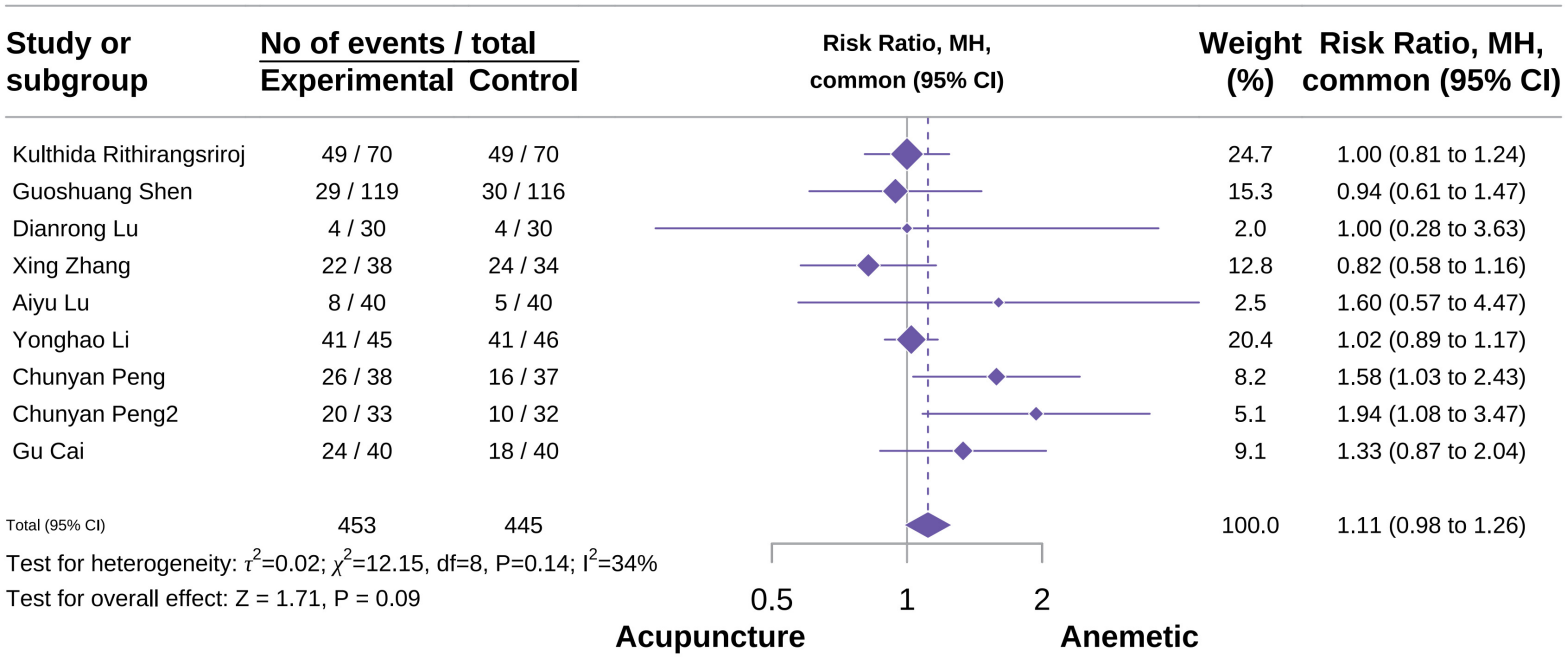
Forest plot of acute complete control rate.

A total of nine studies involving 898 patients reported an acute complete control rate (24, 37, 51, 52, 61, 68, 74, 75, 84). The meta-analysis showed that acupuncture did not significantly improve the acute complete control rate of CINV vs. the control group (RR 1.11, 95% CI 0.98–1.26; *P* = 0.09; *I*^2^ = 34%; Figure 4). Subgroup analyses indicated that neither EA (RR 0.95, 95% CI 0.62–1.44; *P* = 0.81; *I*^2^ = 0%) nor AA (RR 1.09, 95% CI 0.91–1.30; *P* = 0.37; *I*^2^ = 0%) yielded significant benefits. In contrast, QA increased the acute complete control rate (RR 1.56, 95% CI 1.19–2.05; *P* = 0.001; *I*^2^ = 0%). Acupuncture type emerged as a potential source of heterogeneity (see Supplementary Figure S3). Sensitivity analyses revealed that excluding individual studies altered the effect estimates, suggesting that these results should be interpreted with caution (Supplementary Figure S4).

#### Delay complete control rate

**Figure 5:**
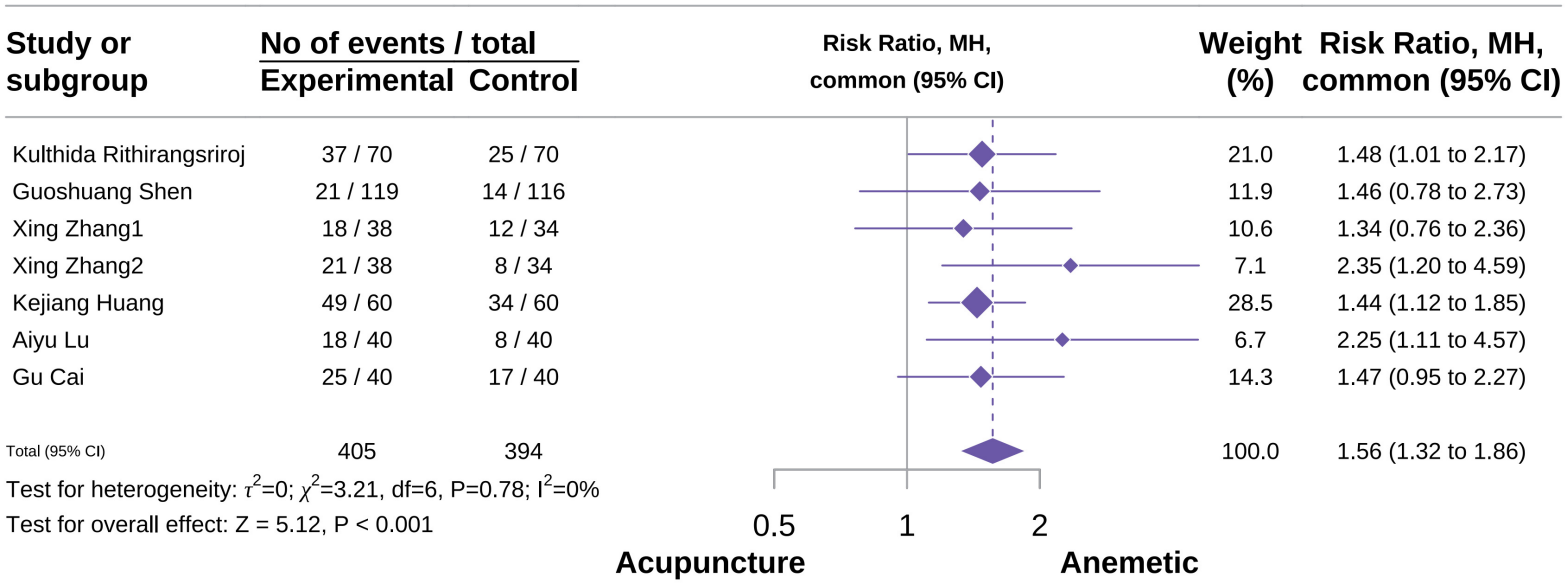
Forest plot of delay complete control rate.

A total of seven studies were included in the meta-analysis for this outcome, comprising 405 patients in the acupuncture group and 394 in the control group (24, 37, 51, 53, 61, 84). The meta-analysis result demonstrated that acupuncture significantly improved the delayed complete control rate compared with the control group, with no observed heterogeneity (RR 1.56, 95% CI 1.32–1.86; *P* < 0.001; *I*^2^ = 0%; Figure 5). In subgroup analysis by acupuncture type, MA significantly enhanced the delay complete control rate (RR 1.70, 95% CI 1.22–2.37; *P* = 0.002; *I*^2^ = 27%). Other acupuncture types lacked sufficient studies to calculate pooled effect sizes; only effect sizes are presented. Sensitivity analyses confirmed the robustness of these results (Supplementary Figures S5, S6).

#### Meta-analysis of second outcome

##### Overall phase

###### Overall no vomiting events

**Figure 6:**
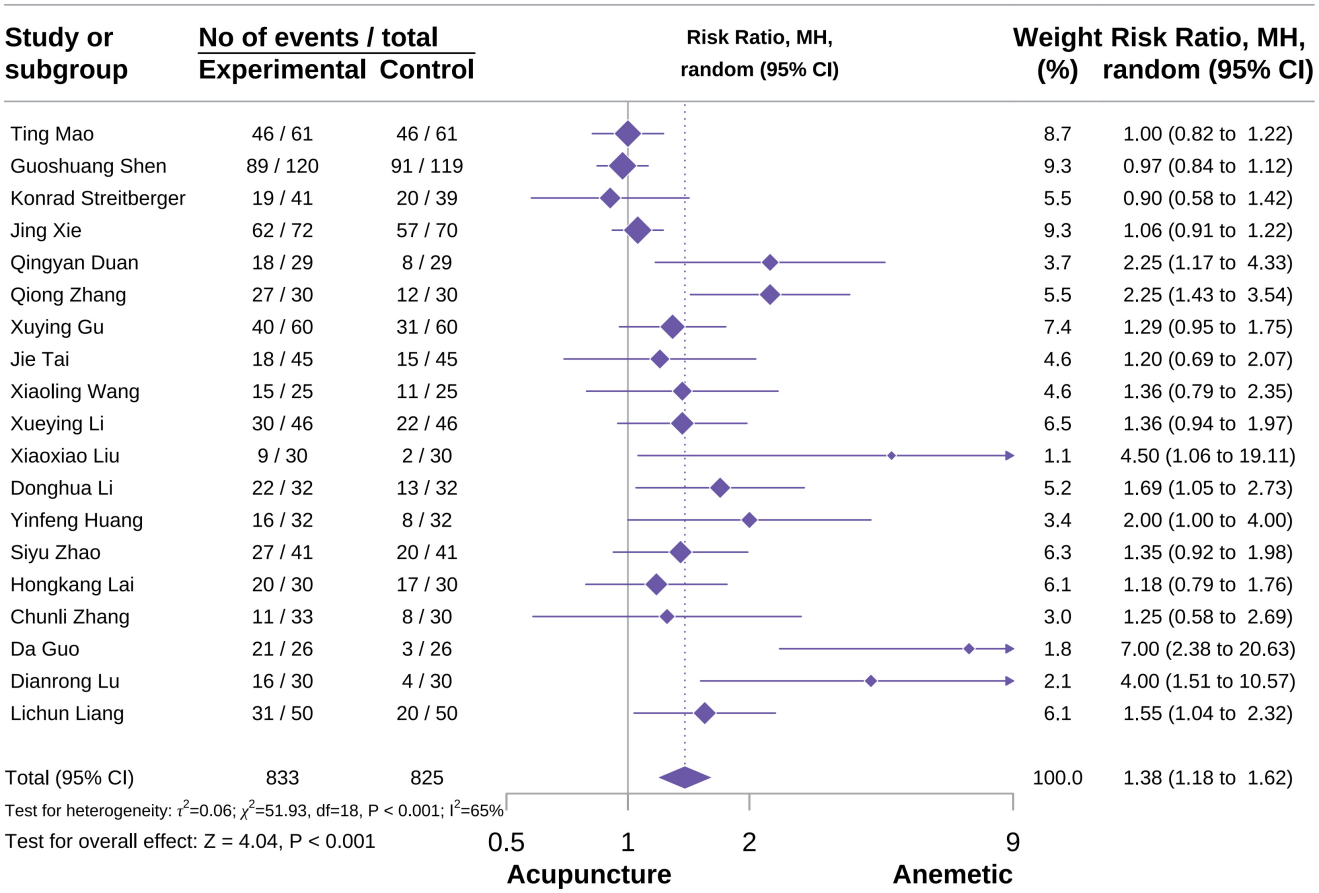
Forest plot of overall no vomiting events.

A total of 19 studies were included in this outcome (22, 24, 36, 39, 46, 49, 50, 52, 57, 58, 64, 66, 71, 76–78, 81, 90, 92). The meta-analysis results showed that acupuncture could effectively improve the overall no vomiting events [RR = 1.38, 95% CI = (1.18–1.62), *P* < 0.001, *I*^2^ = 65%], details shown in Figure 6. According to the subgroup analysis by acupuncture type, the results indicated that acupuncture type was a potential source of heterogeneity. Meanwhile, the subgroup analysis results also showed that EA, TES, and MA could not positively affect to the overall no vomiting events [RR = 1.80, 95% CI = (0.45–7.21), *P* = 0.40, *I*^2^ = 88%; RR = 1.04, 95% CI = (0.92–1.17), *P* = 0.54, *I*^2^ = 0%; RR = 1.39, 95% CI = (1.01–1.93), *P* = 0.04, *I*^2^ = 62%]. In contrast, AA and MA combined with AA could improve the overall no vomiting events [RR = 1.44, 95% CI = (1.08–1.91), *P* = 0.01, *I*^2^ = 14%; RR = 1.58, 95% CI = (1.03–2.42), *P* = 0.04, *I*^2^ = 0%], details shown in Supplementary Figure S1. Sensitivity analysis confirmed the reliability of the results. According to the heterogeneity, we used the Thompson test to precisely detect publication bias, with results showing a certain risk. Specific information on subgroup analysis and publication bias is shown in Supplementary Figure S2.

###### Overall no significant nausea events

**Figure 7:**
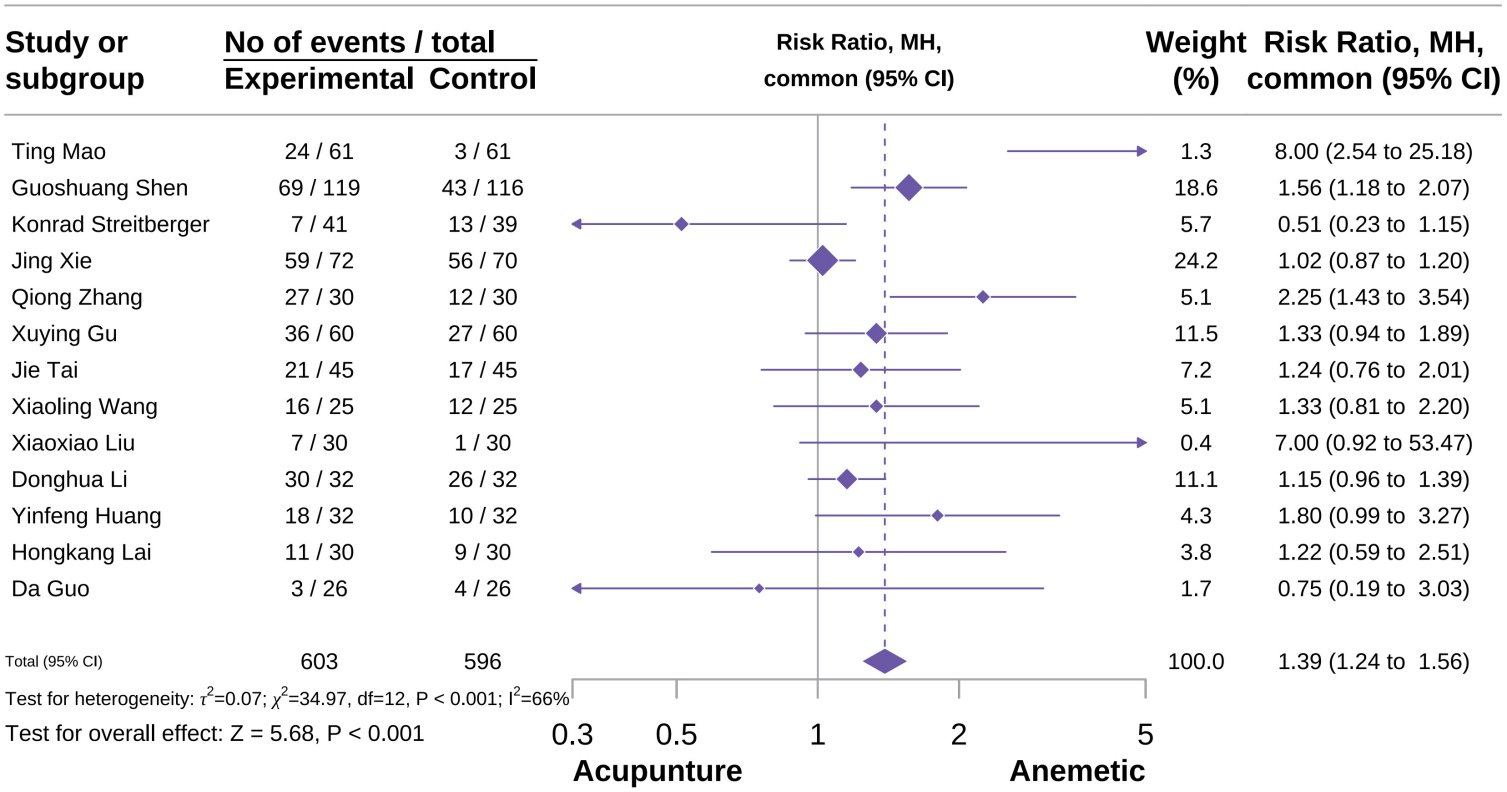
Forest plot of overall no significant nausea events.

A total of 13 studies were included in this outcome meta-analysis. The results showed that compared with the control group, acupuncture effectively improved overall no significant nausea events [RR = 1.37, 95% CI = (1.12–1.68), *P* < 0.001, *I*^2^ = 66%]; for detailed information see Figure 7 (24, 36, 39, 49, 50, 57, 64, 71, 76–78, 90). Subgroup analysis results indicated that acupuncture type was a potential source of heterogeneity. Additionally, subgroup analysis also showed that MA did not have a positive effect on the overall no significant nausea events [RR = 1.15, 95%CI = (0.98–1.35), *P* = 0.08, *I*^2^ = 19%), while AA demonstrated a protective effect [RR = 1.51, 95% CI = (1.03–2.22), *P* = 0.04, *I*^2^ = 0%]. Unfortunately, EA, which has shown advantages in recent studies for the absence of significant nausea, could not be included in the subgroup analysis due to insufficient studies (subgroup detail, see Supplementary Figure S3). Sensitivity analysis confirmed the reliability of the results, and Thompson's test for publication bias indicated no publication bias for this outcome (see Supplementary Figure S4).

###### Overall vomiting severity score

**Figure 8:**
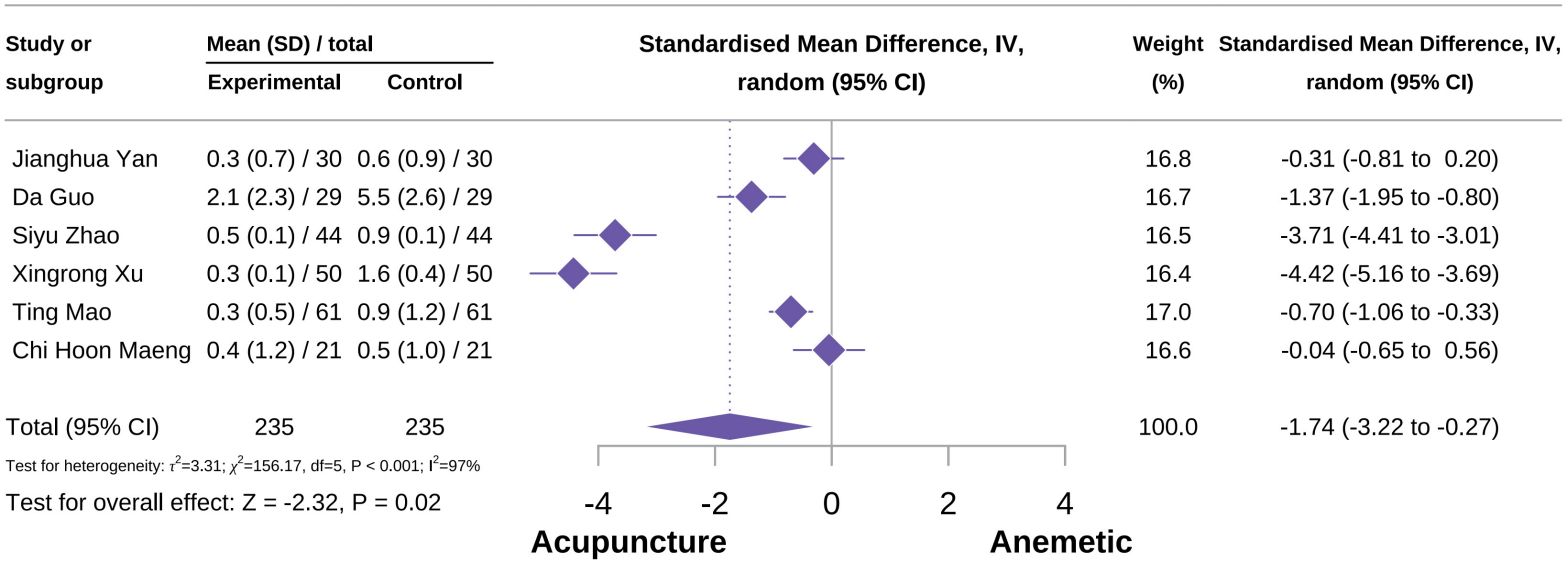
Forest plot of overall vomiting severity score.

A total of six studies were included in this outcome. Meta-analysis results showed that the acupuncture could reduce the overall vomiting severity score [SMD = −1.74, 95% CI = (−3.22 to −0.27), *P* < 0.001, *I*^2^ = 79%], as shown in Figure 8 (34, 36, 54, 71, 81, 85). However, subgroup analysis results indicated that MA had no positive effect on improving the overall vomiting severity score [SMD = −1.53, 95% CI = (−3.48 to 0.43), *P* = 0.13, *I*^2^ = 97%]. Other types of acupuncture lack enough studies to combine effect sizes in subgroup analysis, and the type of acupuncture was a potential source of heterogeneity. Sensitivity analysis results suggested that this outcome was not reliable and should be treated with caution. Specific information on subgroup analysis and sensitivity analysis is supplemented in Supplementary Figure S5.

###### Overall nausea severity score

**Figure 9:**
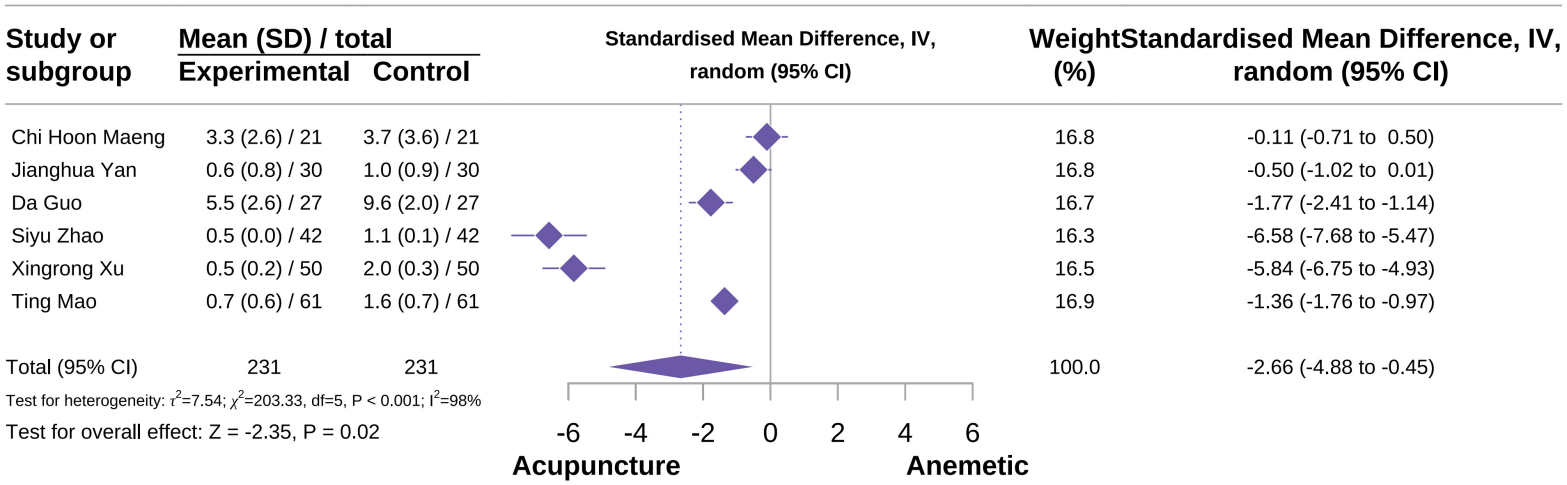
Forest plot of overall nausea severity score.

The results of the meta-analysis showed that acupuncture reduced overall nausea severity scores compared to the control group [SMD = −2.66, 95% CI = (−4.88 to −0.45), *P* = 0.02, *I*^2^ = 98%], see Figure 9 (34, 36, 54, 71, 81, 85). Subgroup analysis based on acupuncture type indicated that different acupuncture types were potential sources of heterogeneity. Additionally, subgroup analysis also showed that MA did not have a positive effect on overall nausea severity scores [SMD = −2.04, 95% CI = (−4.58 to −0.51), *P* = 0.12, *I*^2^ = 98%]. Other acupuncture types could not be further explored due to insufficient studies. Sensitivity analysis results showed that the significance of the differences changed, and this outcome meta-analysis result should be interpreted with caution. Subgroup analysis and sensitivity analysis results are presented in Supplementary Figure S6.

###### Overall nausea frequency score

The meta-analysis included two studies, and the results showed that the acupuncture could not improve the overall nausea frequency score [MD = −1.00, 95% CI (−2.04 to 0.05), *P* = 0.06, *I*^2^ = 93%] (54, 85). Notably, sensitivity analysis results indicated that the meta-analysis effect size changed, suggesting that the results of this outcome meta-analysis should be interpreted with caution. Specific information can be found in Supplementary Figure S7.

###### Overall duration of nausea score

The meta-analysis included two studies, with results showing that the acupuncture could effectively reduce the overall duration of nausea score [MD = −1.08, 95% CI = (−1.70 to −0.46), *P* < 0.001, *I*^2^ = 87%] (54, 85). Sensitivity analysis indicated that the results of this outcome meta-analysis should be interpreted with caution, with specific details available in Supplementary Figure S8.

###### Overall duration of CINV score

**Figure 10:**
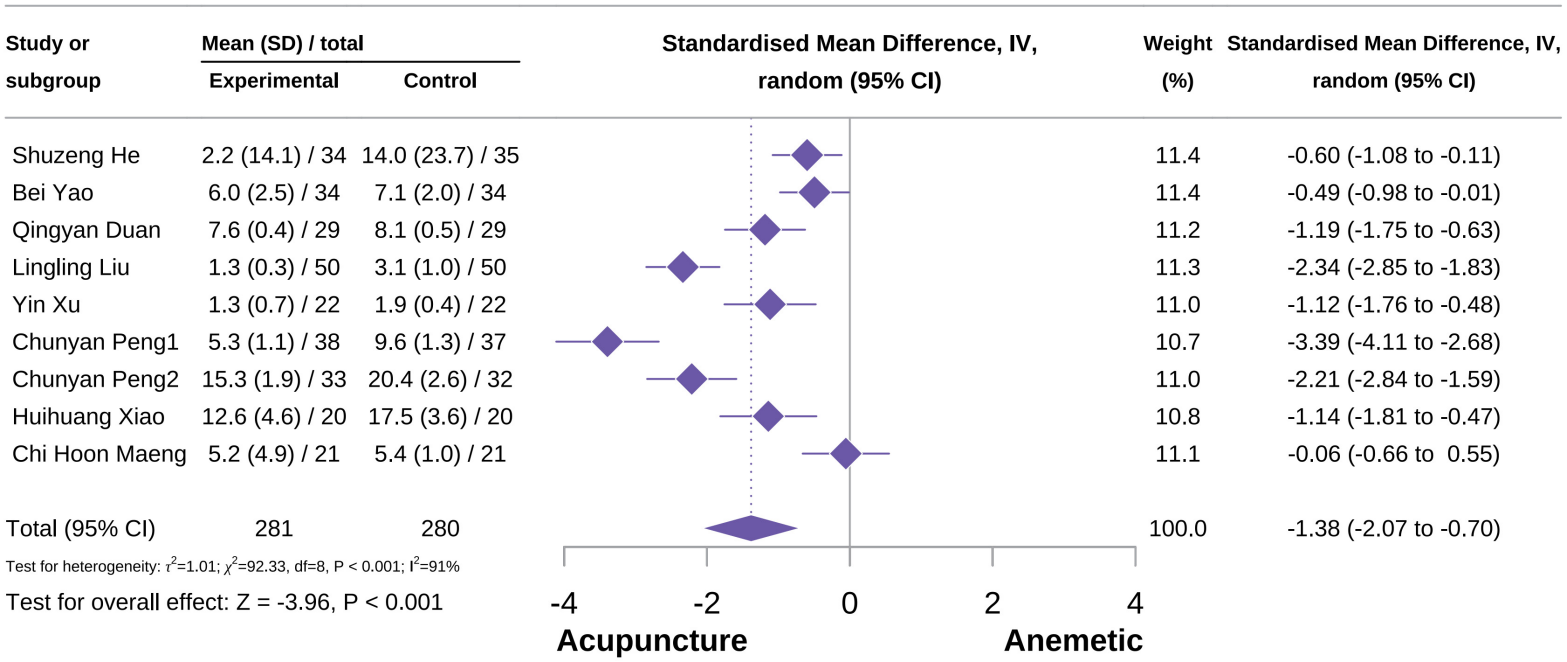
Forest plot of overall CINV score.

The meta-analysis showed that acupuncture had a positive effect on reducing overall CINV score [SMD = −1.38, 95% CI = (−2.07 to −0.70), *P* < 0.001, *I*^2^ = 91%], as shown in Figure 10 (34, 42, 44, 46, 48, 74, 75, 79, 83). Subgroup analysis revealed heterogeneity in the type of acupuncture. Additionally, subgroup analysis also showed that both MA and QA can reduce the overall CINV score [SMD = −1.08, 95% CI = (−1.70 to −0.46), *P* < 0.001, *I*^2^ = 87%; SMD = −2.79, 95% CI = (−3.95 to −1.63), *P* < 0.001, *I*^2^ = 83%]. Sensitivity analysis confirmed the reliability of the outcomes, with detailed information provided in Supplementary Figure S9.

###### Overall vomiting frequency score

The results showed that acupuncture effectively reduced the overall vomiting frequency score [SMD = −1.31, 95% CI = (−2.20 to −0.43), *P* = 0.004, *I*^2^ = 93%] (22, 38, 54, 84, 85). Subgroup analysis indicated that MA had a positive effect on the overall vomiting frequency score [SMD = −1.24, 95% CI = (−2.36 to −0.11), *P* = 0.03, *I*^2^ = 94%]. Additionally, the type of acupuncture was not a potential source of heterogeneity. Sensitivity analysis confirmed the reliability of the outcomes, with detailed information provided in Supplementary Figure S10.

###### Overall vomiting volume score

The result shows that acupuncture could reduce the overall vomiting volume score, compared to the control group [SMD = −2.21,95 %CI = (−6.61 to −2.19), *P* = 0.32, *I*^2^ = 99%]. Sensitivity analysis results indicate that the results for this outcome should be interpreted with caution; specific information is available in Supplementary Figure S11.

##### Acute phase

###### Acute no vomiting events

**Figure 11:**
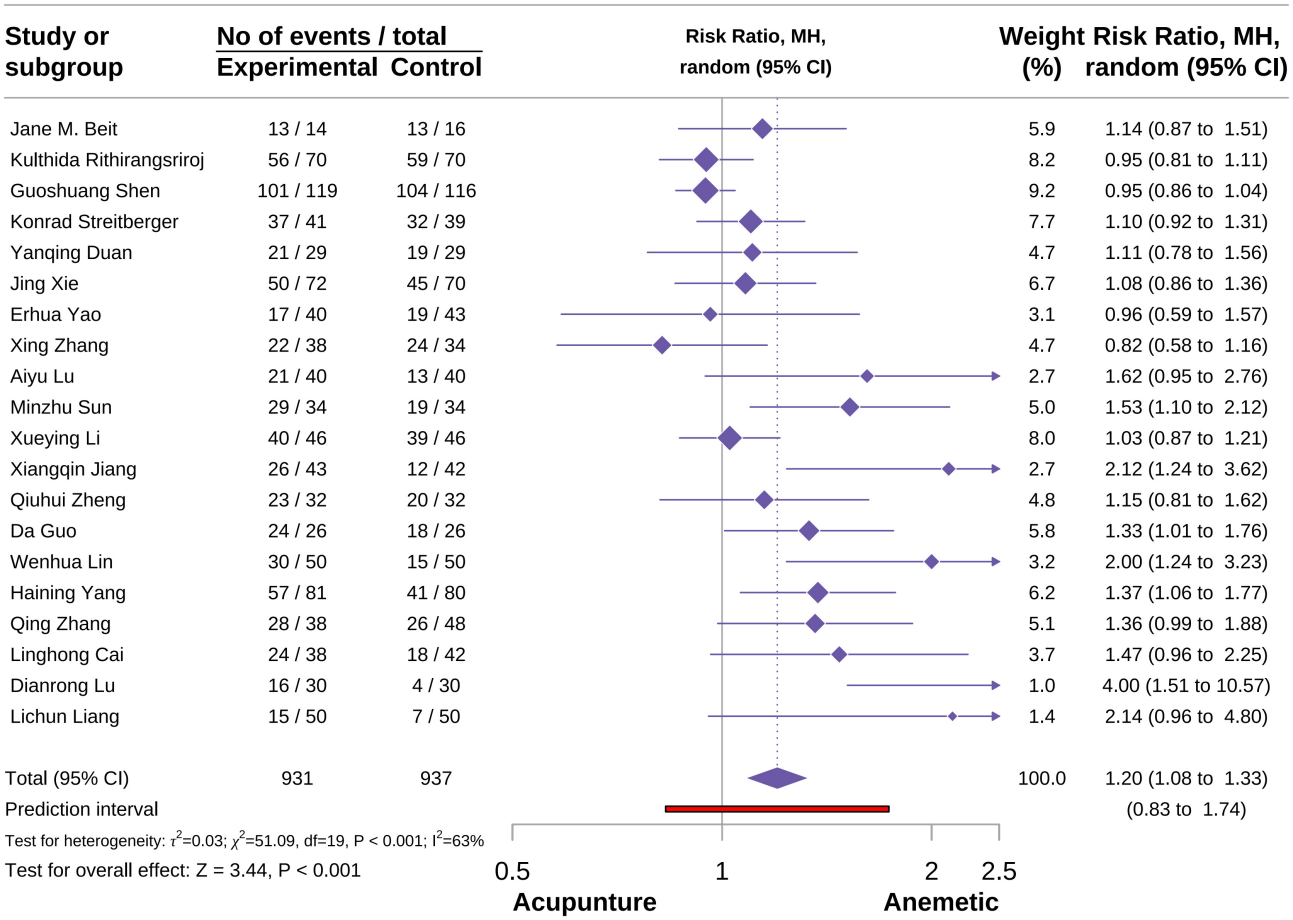
Forest plot of acute no vomiting events.

A total of 20 studies were included in this outcome meta-analysis, comprising 931 participants in the acupuncture group and 937 in the control group (24, 35, 37, 39, 46, 47, 51, 52, 58, 60, 61, 66, 70–73, 82, 89, 91). The meta-analysis result demonstrated that acupuncture significantly increased the incidence of acute no vomiting events [RR = 1.20, 95% CI (1.08–1.33), *P* < 0.001, *I*^2^ = 63%; Figure 11]. Subgroup analyses suggested that the type of acupuncture was a potential source of heterogeneity. Notably, neither MA nor EA showed protective effects on acute no vomiting events [RR = 1.20, 95% CI (1.08–1.33), *P* < 0.001, *I*^2^ = 46%; RR = 1.79, 95% CI (0.44–7.27), *P* > 0.05, *I*^2^ = 88%]. In contrast, AA and AA combined with acupoint acupressure (AP) were associated with increased incidence of acute no vomiting events (Supplementary Figure S1). Sensitivity analyses confirmed the robustness of these results, while publication bias assessment indicated a certain degree of risk (Supplementary Figure S2).

###### Acute no significant nausea events

**Figure 12:**
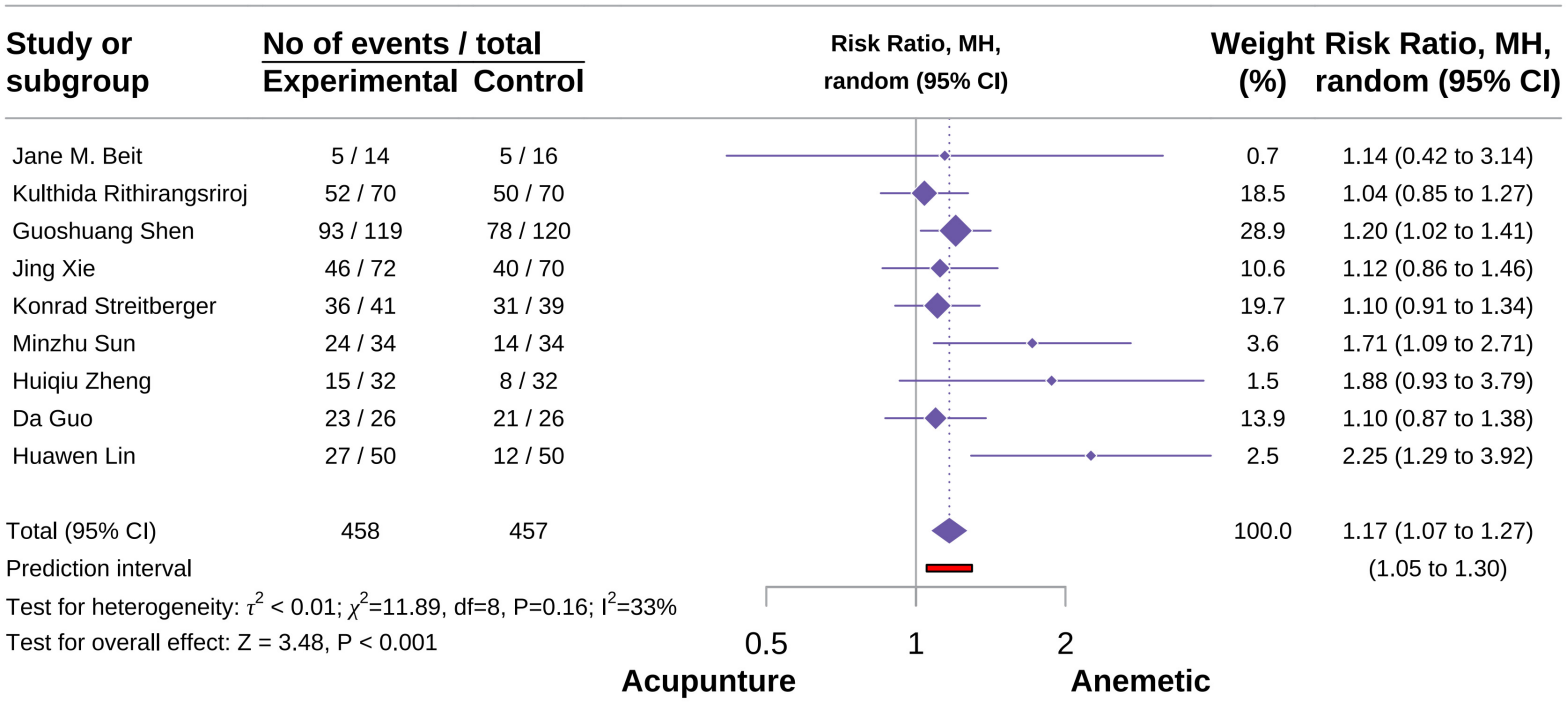
Forest plot of acute no significant nausea events.

A total of nine studies were included in this outcome meta-analysis (22, 24, 35, 37, 39, 60, 71–73). The meta-analysis indicated that acupuncture increased the incidence of acute no-nausea events [RR = 1.17, 95% CI (1.07–1.27), *P* < 0.001, *I*^2^ = 33%; Figure 12]. Subgroup analyses by acupuncture type showed that AA combined with AP exerted a significant beneficial effect with minimal heterogeneity [RR = 1.91, 95% CI (1.34–2.72), *P* < 0.001, *I*^2^ = 0%]. In contrast, MA showed no advantage over control in preventing acute nausea events [RR = 1.08, 95% CI (0.96–1.22), *P* = 0.21, *I*^2^ = 0%]. Sensitivity analyses further supported the reliability of the result (Supplementary Figure S3).

###### Acute vomiting severity score

**Figure 13:**
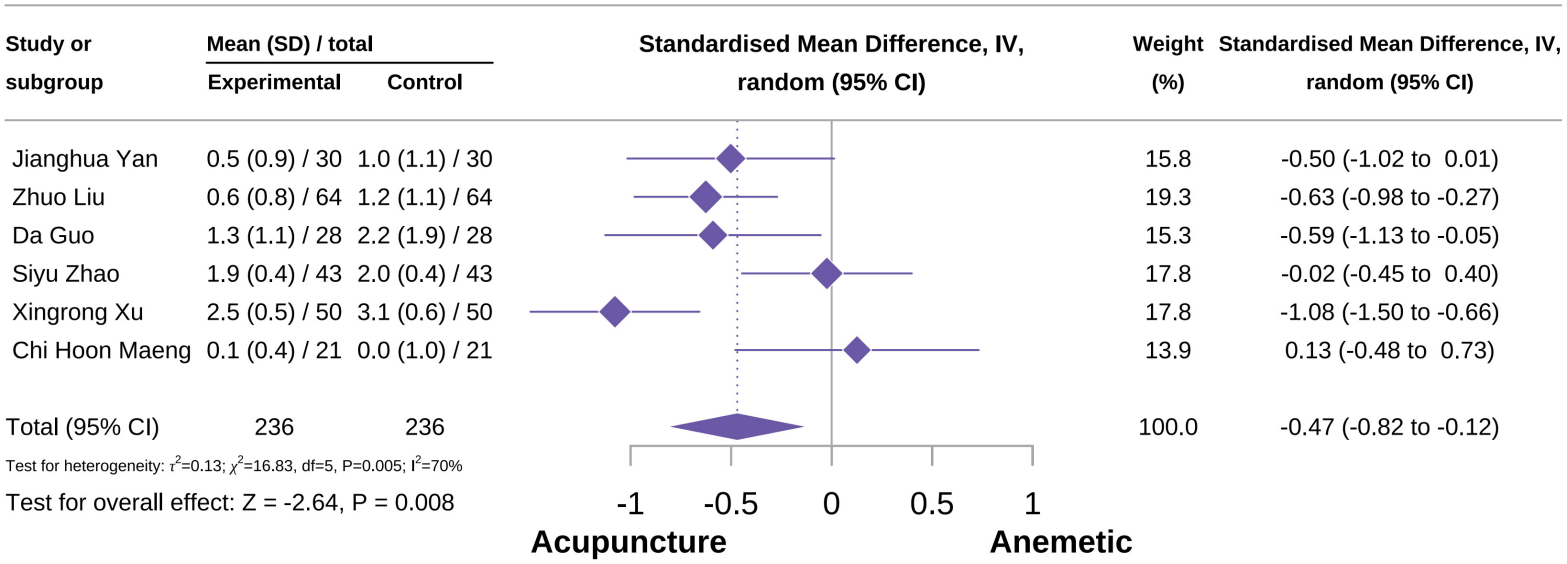
Forest plot of acute vomiting severity score.

A total of six studies were included in this outcome meta-analysis (34, 54, 67, 71, 81, 85). The meta-analysis demonstrated that acupuncture reduced the severity score of acute vomiting [SMD = −0.47, 95% CI (−0.82 to −0.12), *P* = 0.005, *I*^2^ = 70%; Figure 13]. Subgroup analyses indicated that the type of acupuncture was not a potential source of heterogeneity. MA showed a significant beneficial effect in reducing acute vomiting severity [SMD = −0.54, 95% CI (−1.02 to −0.06), *P* = 0.03, *I*^2^ = 72%]. Sensitivity analyses further confirmed the robustness of the result (Supplementary Figure S4).

###### Acute nausea severity score

A total of seven studies were included in this outcome meta-analysis (34, 54, 67, 71, 73, 81, 85). The meta-analysis demonstrated that acupuncture significantly reduced the severity score of acute nausea [SMD = −0.54, 95% CI (−1.02 to −0.06), *P* = 0.03, *I*^2^ = 72%]. Subgroup analyses indicated that neither MA nor AA showed a beneficial effect, and the type of acupuncture was not a potential source of heterogeneity. Sensitivity analyses confirmed the robustness of the result (Supplementary Figure S5).

###### Acute vomiting frequency score

**Figure 14:**
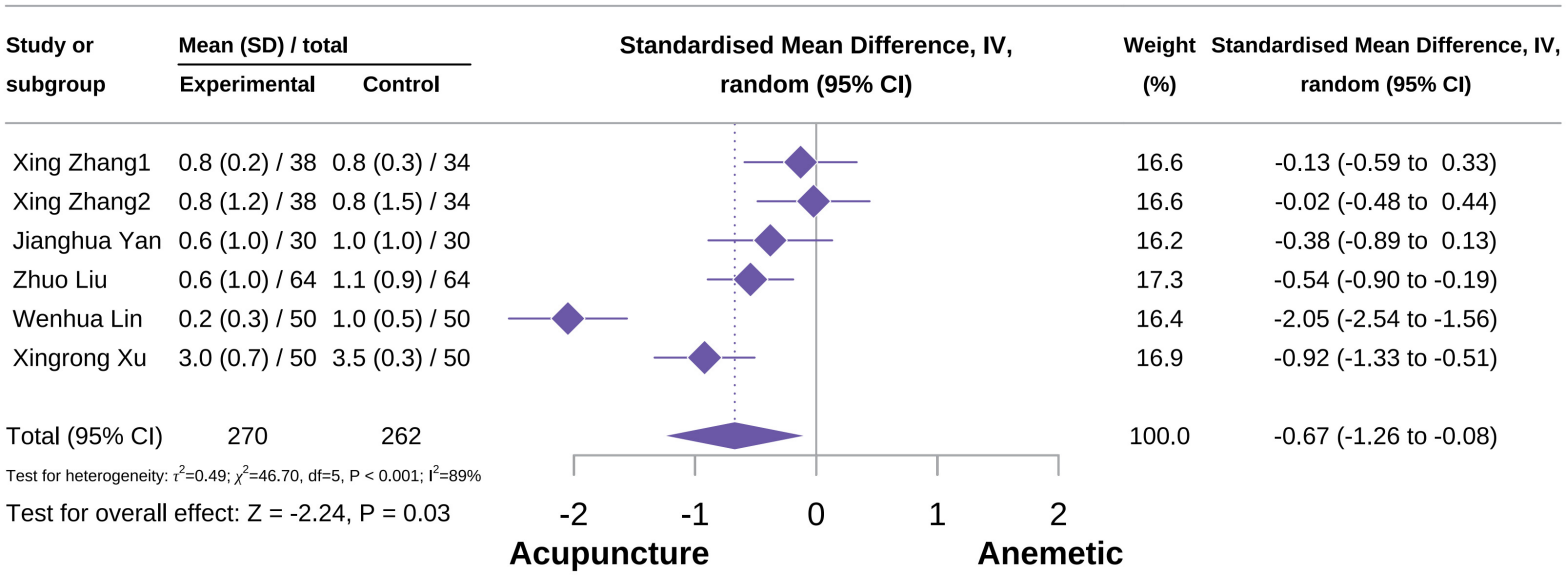
Forest plot of acute vomiting frequency score.

A total of six studies were included in this outcome meta-analysis (51, 54, 67, 73, 85). The meta-analysis indicated that acupuncture significantly reduced the frequency score of acute vomiting compared with the control group [SMD = −0.67, 95% CI (−1.62 to −0.08), *P* = 0.03, *I*^2^ = 89%; Figure 14]. Subgroup analyses suggested that the type of acupuncture was not a potential source of heterogeneity. Neither MA nor AA demonstrated a protective effect on acute vomiting frequency score [SMD = −0.45, 95% CI (−0.98 to 0.08), *P* = 0.09, *I*^2^ = 76%; SMD = −1.29, 95% CI (−2.76 to 0.19), *P* = 0.09, *I*^2^ = 96%]. Notably, sensitivity analyses indicated that the result should be interpreted with caution (Supplementary Figure S6).

###### Acute vomiting volume score

A total of three studies were included in this outcome meta-analysis (54, 67, 85). The meta-analysis demonstrated that acupuncture improves the acute volume score compared with the control group [MD = −0.30, 95% CI (−0.48 to −0.12), *P* = 0.001, *I*^2^ = 0%]. Subgroup analyses solely indicated that MA showed a significant advantage [MD = −0.30, 95% CI (−0.52 to −0.08), *P* = 0.008, *I*^2^ = 0%]. Sensitivity analyses confirmed the robustness of the result (Supplementary Figure S7).

###### Acute nausea duration score

A total of three studies were included in this outcome meta-analysis (54, 67, 85). The meta-analysis demonstrated that acupuncture significantly reduced the acute nausea duration score compared with the control group [MD = −0.62, 95% CI (−0.79 to −0.41), *P* < 0.001, *I*^2^ = 0%]. Subgroup analyses indicated that only MA showed a significant beneficial effect [MD = −0.61, 95% CI (−0.79 to −0.43), *P* < 0.001, *I*^2^ = 0%]. Sensitivity analyses confirmed the robustness of the result (Supplementary Figure S8).

###### Acute nausea frequency score

The meta-analysis demonstrated that acupuncture shows a beneficial effect in reducing the frequency score of acute nausea [MD = −0.62, 95% CI (−0.74 to −0.50), *P* < 0.001, *I*^2^ = 0%] (54, 67, 85). Subgroup analyses indicated that only MA showed a significant advantage (MD = −0.61, 95% CI [−0.73 to −0.48], *P* < 0.001, *I*^2^ = 0%). Sensitivity analyses confirmed the reliability of the result (Supplementary Figure S9).

##### Delay phase

###### Delay no vomiting events

**Figure 15:**
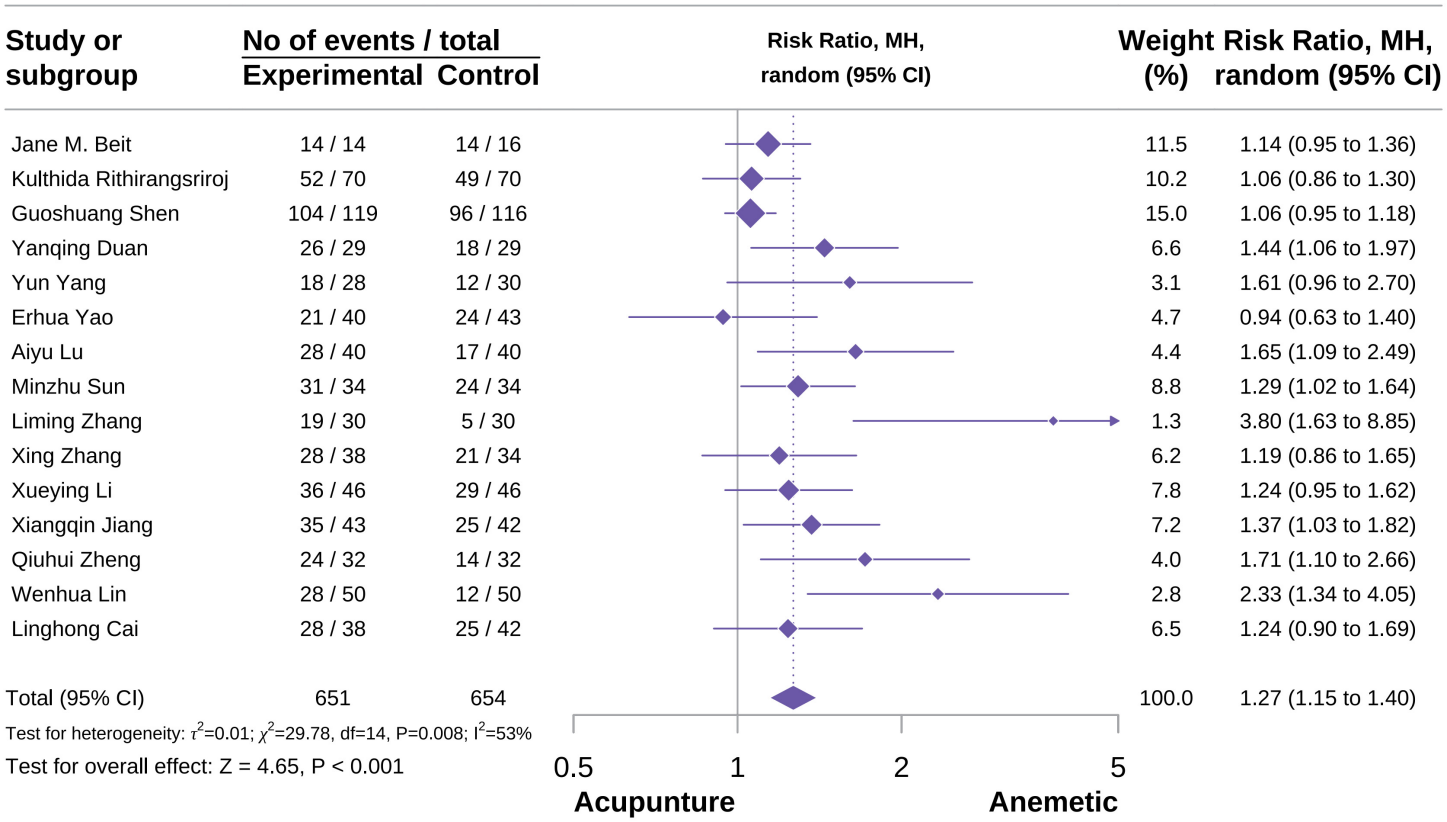
Forest plot of delay no vomiting events.

A total of 15 studies were included in this outcome meta-analysis (24, 35, 37, 45–47, 51, 60, 61, 65, 66, 70, 72, 73, 91). The meta-analysis demonstrated that acupuncture significantly increased the incidence of delayed no vomiting events compared with the control group [RR = 1.27, 95% CI (1.15–1.40), *P* < 0.001, *I*^2^ = 53%; Figure 15]. Subgroup analyses showed that both MA and AA were associated with beneficial effects [RR = 1.13, 95% CI (1.02–1.26), *P* = 0.02, *I*^2^ = 0%; AA: RR = 1.78, 95% CI [1.12–2.82], *P* = 0.01, *I*^2^ = 61%), whereas the results for AA combined with AP were not significant [RR = 1.64, 95% CI (0.93–2.91), *P* = 0.09, *I*^2^ = 73%; Supplementary Figure S1]. Subgroup analyses also suggested that acupuncture type was a potential source of heterogeneity. Sensitivity analyses confirmed the robustness of the result, while the publication bias result indicated some degree of risk (Supplementary Figure S2).

###### Delayed no significant nausea events

A total of eight studies were included in this outcome meta-analysis (24, 37, 51, 60, 65, 72, 73). The meta-analysis demonstrated that acupuncture significantly improved delayed no significant nausea events [RR = 1.48, 95% CI (1.32–1.70), *P* < 0.001, *I*^2^ = 0%; Supplementary Figure S3]. Subgroup analyses further revealed differences in efficacy according to acupuncture type. MA showed a significant advantage [RR = 1.75, 95% CI (1.25–2.45), *P* < 0.001, *I*^2^ = 0%], whereas AA combined with AP did not demonstrate a significant effect [RR = 1.79, 95% CI (0.95–3.37), *P* = 0.07, *I*^2^ = 0%; Supplementary Figure S4]. Sensitivity analyses confirmed the robustness of the result.

###### Delayed nausea severity score

The meta-analysis demonstrated that acupuncture reduced the severity score of delayed nausea compared with the control group [SMD = −2.16, 95% CI (−3.55 to −0.78), *P* = 0.002, *I*^2^ = 95%] (51, 73). Due to the limited number of studies, subgroup analyses could only present effect estimates according to acupuncture type. Notably, sensitivity analyses showed that the result should be interpreted with caution (Supplementary Figure S5).

#### Evidence quality assessment

The GRADE system (101) was applied to evaluate the quality of evidence for 23 meta-analyses (24, 34–91). We classified the primary outcomes as critical and the secondary outcomes as important in terms of clinical importance. The results showed that the primary outcomes, including complete control rates during overall, acute, and delayed phases, had major limitations across all five downgrading domains and were rated as very “low quality.” Among the secondary outcomes, only the acute duration of nausea score was rated as “moderate quality,” and the acute nausea frequency score as “low quality”; all other outcomes were rated as “very low” quality. In total, 21 outcomes were graded as “very low,” 1 as “moderate,” and 1 as “low.” Detailed information is presented in Supplementary Figure S6.

## Discussion

### Summary of the key finding

This meta-analysis included 58 studies involving 4,685 patients with CINV (24, 34–91). To the best of our knowledge, it is the largest evidence synthesis to date on acupuncture for CINV caused by HEC. We synthesized a broad set of discrete outcomes, beginning with the composite outcome of complete control, and then evaluating two domains—vomiting and nausea. Within the vomiting domain, we assessed no vomiting events, severity, frequency, and volume; within the nausea domain, we assessed no nausea events, severity, frequency, duration, and the CINV score. We further stratified effects by phase (overall, acute, and delayed) to comprehensively evaluate the effectiveness of acupuncture for HEC-induced CINV. Overall, the meta-analysis suggested that acupuncture yielded more favorable effects for the complete control rate and vomiting-related outcomes than for nausea-related outcomes (24, 34–91). Several outcomes were unstable in sensitivity analyses, indicating that those results should be interpreted with caution. Following a pre-specified plan, we conducted subgroup analyses by acupuncture type, which partially explained the observed heterogeneity. However, residual heterogeneity persisted. We speculate that this may be due to different characteristics among the studies, including cancer types, outcome assessment instruments, and timing of acupuncture interventions. Unfortunately, because characteristics were missing in some studies, additional subgroup or meta-regression analyses could not be performed to further clarify these heterogeneity sources. In addition, subgroup results also indicated that different acupuncture types did not exert uniform effects across outcomes, and their advantages differed. In the risk of bias domain, most included studies were judged to be at high risk of bias. Combined with the risk of bias and the meta-analytic result, most outcomes were rated as very low in evidence quality assessment. More importantly, almost all studies included in this meta-analysis were conducted in Asian regions, and participants predominantly comprised Asian populations with a relatively high acceptance of acupuncture, whereas studies from other regions and in different populations remain relatively scarce. Against this background, it is unclear whether populations with different genetic characteristics from other regions, and those with lower acceptance of acupuncture, would experience similar therapeutic effects. In other words, the findings of this meta-analysis are subject to geographical and population-related biases, and their extrapolability and generalizability are therefore somewhat limited. Additionally, due to insufficient information in the included studies, we were unable to further analyze the efficacy of acupuncture across different cancer types, which is another factor affecting the generalizability of the results. In summary, while the results suggest that acupuncture may confer benefits for HEC-induced CINV, the overall effectiveness remains uncertain given the various limitations identified. Additional rigorously designed, multi-regional randomized trials are required, confirming the efficacy of acupuncture across diverse participants and clinical settings to improve the certainty of evidence.

### Clinical implication

Over the past four decades, pharmacologic prophylaxis for CINV has achieved milestone advances (6, 13, 93). Nevertheless, a substantial number of patients remain non-responsive to standard regimens recommended by clinical guidelines, particularly those receiving HEC (7, 10). In the context of multimodality cancer treatment, many patients have begun to shift their priorities when selecting therapies, seeking options that control CINV while minimizing adverse effects (7, 94). Therefore, as patient-centered evidence-based medicine has advanced, the search for effective complementary and alternative therapies has become a prominent focus in CINV clinical practice (7, 94). Acupuncture, a key non-pharmacologic intervention, has attracted increasing attention in this setting (7, 94). However, evidence information to acupuncture for HEC-induced CINV remains limited. This meta-analysis supplemented the evidence. In the overall phase, acupuncture favored all vomiting-related outcomes except vomiting volume; in the acute phase, benefits were observed for all vomiting-related outcomes except vomiting frequency score. For delayed-phase vomiting outcomes, due to the lack of included studies, improvement was observed only for delayed no vomiting events. Notably, the meta-analytic results for nausea-related outcomes were somewhat contradictory. Acupuncture appeared to protect against the occurrence of nausea events across all phases, yet it did not demonstrate benefits for subjective outcomes such as nausea severity. Although the sets of included studies differed across outcomes, this constitutes a practical “paradox.” Moreover, the paradox is at odds with a recent study in which EA combined with standard therapy improved both the complete control rate and the visual analog scale (VAS) score for nausea, suggesting that acupuncture may confer dual benefits (24). Improvements in the occurrence of nausea would ordinarily be expected to coincide with improvements in patient-reported nausea outcomes; the reasons for this discrepancy warrant further investigation and create uncertainty regarding the optimal timing of acupuncture for HEC-related CINV in clinical practice. Given that the certainty of efficacy remains unresolved and that no consensus exists on acupuncture modality, treatment duration, or timing, it is appropriate in clinical practice to follow the 2023 MASCC/ESMO expert consensus on non-pharmacologic management of CINV, which recommends offering acupuncture as an adjunct to standard antiemetic therapy when appropriate, with particular emphasis in the acute phase to address vomiting events (94). Taken together, this meta-analysis provides supplementary evidence for clinicians and guideline developers regarding the effectiveness of acupuncture for CINV in patients receiving HEC. Notably, the meta-analytic results for nausea-related outcomes were somewhat contradictory. Acupuncture can protect against the occurrence of nausea events across all phases, yet it did not demonstrate benefits for subjective outcomes such as nausea severity. Although the sets of included studies differed across outcomes, this constitutes a practical “paradox.” Moreover, the paradox is at odds with a recent study in which EA combined with standard therapy improved both the complete control rate and the visual analog scale (VAS) score for nausea, suggesting that acupuncture may confer dual benefits (24). Improvements in the no nausea events would ordinarily be expected to coincide with improvements in patient-reported nausea outcomes; the reasons for this discrepancy warrant further investigation and create uncertainty regarding the optimal timing of acupuncture for HEC-related CINV in clinical practice. Given that the certainty of efficacy remains unresolved and that no consensus exists on acupuncture modality, treatment duration, or timing, it is appropriate in clinical practice to follow the 2023 MASCC/ESMO expert consensus on non-pharmacologic management of CINV, which recommends offering acupuncture as an adjunct to standard antiemetic therapy when appropriate, with particular emphasis in the acute phase to address vomiting events (94). Taken together, this meta-analysis provides supplementary evidence for clinicians and guideline developers regarding the effectiveness of acupuncture for CINV in patients receiving HEC.

### Future implication

First, resolving the contradictory result regarding nausea outcomes is of substantial importance for future research. We further analyzed the general characteristics of the included studies and found virtually no overlap in the selection of instruments used to assess subjective nausea outcomes. The same situation was observed for instruments assessing subjective vomiting symptoms, indirectly indicating that current studies have not reached consensus on the choice of outcome instruments and that the tools with optimal sensitivity and specificity have not been determined. The appropriateness of outcome instrument selection is unquestionably critical for accurately reflecting treatment effects (95). Additionally, heterogeneity in outcome measurement instruments undermines direct assessment of treatment effects across studies and can even impede evidence synthesis, thereby delaying the timely identification and translation of effective interventions into clinical practice, particularly for major diseases (95, 96). In many disease areas, a Core Outcome Set (COS) has been developed to address the disorder in outcome measurement instruments and the inappropriate selection of outcomes. The purpose of establishing a COS is to ensure that the outcomes selected in studies necessarily include the core endpoints that are fundamental and of greatest concern to clinicians and patients. Establishing an effective COS can reduce heterogeneity between studies, improve the quality of evidence synthesis, and mitigate reporting bias. However, a COS has not yet been established for acupuncture in the management of CINV. Therefore, future studies of acupuncture for CINV should establish and adopt a COS and, on that basis, identify or develop outcome measurement instruments that adequately capture the therapeutic effects of acupuncture. Additionally, analysis of the general characteristics of the included studies revealed that the delayed phase was often poorly defined; many studies did not clearly delineate the delayed-phase time window. This may be one factor contributing to differences observed in sensitivity analyses and to the substantial heterogeneity of the meta-analysis. In authoritative clinical practice guidance, including ASCO, the delayed phase is clearly defined as 24–120 h (6, 7). We therefore urge that future studies of acupuncture for CINV adhere to the prevailing gold-standard definition of the delayed phase to enhance methodological rigor and improve the quality of evidence (13).

Against the backdrop of the close integration of evidence-based medicine and acupuncture clinical research, RCTs have become the gold standard for evaluating efficacy because they can adequately control bias (confounding) and support causal inference, which is why RCTs stand at the apex of the evidence hierarchy. It should be noted that this “gold-standard” status presupposes sufficient methodological soundness and rigor. However, according to the risk-of-bias assessments in this meta-analysis, the reliability and rigor of RCTs on this topic are concerning and resulted in substantial downgrading of the certainty of evidence, with concrete problems concentrated in three domains: randomization, blinding, and statistical analysis. Randomization is the critical safeguard against bias in RCTs. Its importance lies in three areas: first, the unpredictability of assignment to reduce selection bias; second, precision in allocation to minimize between-group size imbalance to reduce allocation bias; and third, covariate balance between groups to ensure that factors influencing treatment outcomes are approximately equal at baseline, thereby allowing statistical inference to attribute observed differences solely to the intervention effect (97, 98). The progression from early simple randomization to commonly used block and stratified randomization, and more recently to Restricted Randomization Methods with Maximum Tolerated Imbalance (MTI), underscores the importance of randomization (97). In this meta-analysis, most included studies provided little detail on randomization and merely stated that a random method was used. This lack of specificity significantly undermines transparency and rigor in trials of acupuncture for HEC-related CINV. Future studies should ensure complete and accurate reporting of randomization procedures. Furthermore, because it is difficult to blind the acupuncturist, given the particularities of acupuncture practice, the credibility of the blinding domain has been questioned in some studies. Under these circumstances, ensuring end-to-end participant blinding is especially critical. However, among the studies included in this meta-analysis, sham acupuncture, which is the best method for blinding participants in acupuncture research, was underutilized. Although the “placebo” properties of sham acupuncture as a control remain controversial, it is currently the best available approach to achieve participant blinding throughout acupuncture trials. With respect to statistical analysis, the studies included here commonly did not specify the primary analysis set and misapplied statistical methods. Because statistical analysis is the final critical step that yields estimates of treatment effect, such deficiencies can exaggerate or underestimate the true effect of acupuncture. Notably, all of the above issues are explicitly addressed in CONSORT 2025 (99), which indirectly indicates that current acupuncture clinical researchers have not fully understood clinical reporting standards. In sum, studies of acupuncture for HEC-related CINV urgently require improvements in these areas, and high-quality randomized controlled trials are needed to strengthen the evidence base and inform clinical practice guideline development.

### Limitations and advantages

This meta-analysis has several limitations. First, although no language restrictions were applied during the search, the eventual inclusion of studies published only in Chinese or English may introduce a potential for language bias. Second, the included studies were conducted in specific regions with relatively homogeneous population characteristics, and did not allow an assessment of the efficacy of acupuncture across different cancer types, thereby restricting the generalizability of the results. Third, insufficient reporting of study characteristics in the included studies limited our ability to fully explore the substantial heterogeneity observed through additional subgroup or meta-regression analyses. Despite these limitations, our study has notable strengths. However, this study also has advantages. We collated a large volume of discrete outcome data and comprehensively assessed the efficacy of acupuncture across multiple outcomes of HEC-induced CINV. Subgroup analyses further reflected the effects of different acupuncture modalities on CINV. Although interpretation must be cautious given the overall quality of the included trials, our review still provides a useful supplement to the evidence base.

## Conclusion

Acupuncture for HEC-induced CINV shows some positive effects. However, considering various limitations render the current evidence insufficient to conclusively establish its efficacy; therefore, further high-quality studies are required.

## Data Availability

The original contributions presented in the study are included in the article/Supplementary material, further inquiries can be directed to the corresponding authors.
