# Supplementary material for: Acupuncture for nausea and vomiting induced by highly emetogenic chemotherapy: a systematic review and meta-analysis
Source: Front Neurol. 2026 Jan 5;16:1692411. doi: 10.3389/fneur.2025.1692411 (PMC12812548; doi:10.3389/fneur.2025.1692411)
Supplement: Supplementary file 1 [file Table_1.docx]

**Supplementary material table**

**Sorted by the order in which the articles appear**

1. **Table S1 HEC category**
2. **Table S2 Search Strategy**
3. **Table S3 Reasons for article exclusion after full-text reading**

**Table S1 HEC category**

| **ASCO** |
| --- |
| Anthracycline/cyclophosphamide combination |
| Carmustine |
| Cisplatin |
| Cyclophosphamide >1,500 mg/m2 |
| Dacarbazine |
| Mechlorethamine |
| Streptozocin |
| **CSCO** |
| AC regimen (anthracycline containing plus cyclophosphamide combination regimen) |
| Cyclophosphamide ≥1.5 g/m² |
| Ifosfamide ≥2 g/m² per dose |
| Carboplatin with area under the curve (AUC) ≥4 |
| Dacarbazine |
| Streptozocin |
| Carmustine (BCNU) >250 mg/m² |
| Doxorubicin ≥60 mg/m² |
| Cisplatin |
| Melphalan ≥140 mg/m² |
| Mechlorethamine (mustine) |

**Table S2 Search strategy**

| **PubMed** |
| --- |
| #1：("Acupuncture Therapy"[MeSH Terms] OR "acupuncture, ear"[MeSH Terms] OR "Acupuncture"[MeSH Terms] OR ("Pharmacopuncture"[Title/Abstract] OR "acupuncture treatment"[Title/Abstract] OR "acupuncture treatments"[Title/Abstract] OR "therapy acupuncture"[Title/Abstract] OR "pharmacoacupuncture treatment"[Title/Abstract] OR "pharmacoacupuncture therapy"[Title/Abstract] ] |
| #2: ("Drug Therapy"[MeSH Terms] OR "Drug Therapy"[MeSH Subheading] OR "chemotherapy, adjuvant"[MeSH Terms] OR "Antineoplastic Combined Chemotherapy Protocols"[MeSH Terms] OR "Hyperthermic Intraperitoneal Chemotherapy"[MeSH Terms] OR "Antineoplastic Agents"[MeSH Terms] OR "Neoadjuvant Therapy"[MeSH Terms] OR "drug therapy, combination"[MeSH Terms] OR ("Chemotherapy"[Title/Abstract] OR "Chemotherapies"[Title/Abstract] OR "Pharmacotherapy"[Title/Abstract] OR "Pharmacotherapies"[Title/Abstract] OR "therapy drug"[Title/Abstract] OR "drug therapies"[Title/Abstract] |
| #3：("Nausea"[MeSH Terms] OR "Vomiting"[MeSH Terms] OR "Emesis"[Title/Abstract]) |
| #4: #1 AND #2 AND #3 |
| #8:(randomized controlled trial[pt] OR controlled clinical trial[pt] OR clinical trials as topic[mesh:noexp] OR trial[ti] OR random*[tiab] OR placebo*[tiab]) |
| #9：#4 AND #8 |

| **Embase** |
| --- |
| #1:'acupuncture'/exp OR acupuncture |
| #2:'pharmacopuncture':ab,ti OR 'acupuncture treatment':ab,ti OR 'acupuncture treatments':ab,ti OR 'treatment, acupuncture':ab,ti OR 'therapy, acupuncture':ab,ti OR 'pharmacoacupuncture treatment':ab,ti OR 'treatment, pharmacoacupuncture':ab,ti OR 'pharmacoacupuncture therapy':ab,ti OR 'therapy, pharmacoacupuncture':ab,ti OR 'acupotomy':ab,ti OR 'acupotomies':ab,ti OR 'acupunctures, ear':ab,ti OR 'ear acupunctures':ab,ti OR 'auricular acupuncture':ab,ti OR 'ear acupuncture':ab,ti OR 'acupuncture, auricular':ab,ti OR 'acupunctures, auricular':ab,ti |
| **#3:#1 OR #2** |
| #4: 'chemotherapy'/exp |
| #5: 'chemotherapies':ab,ti OR 'pharmacotherapies':ab,ti OR 'therapy, drug':ab,ti OR 'drug therapies':ab,ti OR 'therapies, drug':ab,ti OR 'pharmacologic therapy':ab,ti OR 'pharmacotherapy':ab,ti OR 'chemotherapy':ab,ti OR 'adjuvant drug therapy':ab,ti OR 'drug therapy, adjuvant':ab,ti OR 'adjuvant chemotherapy':ab,ti OR 'combined antineoplastic agents':ab,ti OR 'antineoplastic agents, combined':ab,ti OR 'agent, combined antineoplastic':ab,ti OR 'neoadjuvant treatments':ab,ti OR 'treatment, neoadjuvant':ab,ti OR 'neoadjuvant chemotherapies':ab,ti OR 'neoadjuvant chemotherapy treatment':ab,ti OR 'chemotherapy treatment, neoadjuvant':ab,ti OR 'neoadjuvant chemotherapy treatments':ab,ti OR 'treatment, neoadjuvant chemotherapy':ab,ti OR 'chemotherapy, combination':ab,ti OR 'chemotherapies, combination':ab,ti OR 'combination chemotherapies':ab,ti OR 'combination chemotherapy':ab,ti OR 'combination drug therapy':ab,ti OR 'combination drug therapies':ab,ti OR 'drug therapies, combination':ab,ti OR 'therapy, combination drug':ab,ti OR 'drug polytherapy':ab,ti OR 'drug polytherapies':ab,ti OR 'polytherapies, drug':ab,ti OR 'polytherapy, drug':ab,ti OR 'polychemotherapy':ab,ti OR 'polychemotherapies':ab,ti OR 'drug therapy':ab,ti OR 'chemotherapy, adjuvant':ab,ti OR 'antineoplastic combined chemotherapy protocols':ab,ti OR 'hyperthermic intraperitoneal chemotherapy':ab,ti OR 'antineoplastic agents':ab,ti OR 'neoadjuvant therapy':ab,ti |
| **#6:#4 OR #5** |
| #7: 'nausea'/exp or 'vomiting'/exp |
| #8: 'vomiting':ab,ti OR 'emesis':ab,ti OR 'nausea':ab,ti |
| **#9：#7 OR #8** |
| #10:'randomized controlled trial':ab,ti OR 'controlled clinical trial':ab,ti OR 'clinical trials as topic':ab,ti OR 'trial':ab,ti OR 'random*':ab,ti OR 'placebo*':ab,ti OR 'search*':ab,ti OR 'rct':ab,ti |
| **#11: #3 AND #6 AND #9** |

| **Web of Science(WOS)** |
| --- |
| TS=( acupuncture OR acupunctur* OR acupoint* OR Pharmacoacupuncture OR electroacupunctur* OR electro-acupunctur* OR electric stimulation therap* OR percutaneous electrical nerve stimulat* OR PENS OR transcutaneous electric nerve stimulated* OR TENS OR auriculotherap* OR auriculoacupunct* OR fire needle ) AND TS=(Chemotherapy OR Chemotherapies OR Pharmacotherapy OR Pharmacotherapies OR Therapy, Drug OR Drug Therapies OR Therapies, Drug OR pharmacologic therapy OR pharmacotherapy OR Chemotherapy OR Adjuvant Drug Therapy OR Drug Therapy OR Chemotherapy Drugs, Cancer OR Drugs, Cancer Chemotherapy OR Chemotherapeutic Anticancer Agents OR Agents, Chemotherapeutic Anticancer OR Chemotherapeutic Anticancer Dug OR Drug, Chemotherapeutic Anticancer OR Cancer Chemotherapy Drug OR Chemotherapy Drug, Cancer OR Drug) AND TS=(randomized controlled trial OR controlled clinical trial OR clinical trials as topic OR trial OR random* OR placebo* OR search* OR rct) |

| **Cochrane** |
| --- |
| #1:MeSH descriptor: [Acupuncture] explode all trees |
| #2:(Acupuncture Therapy):ti,ab,kw OR (Acupuncture, Ear):ti,ab,kw OR (Acupuncture):ti,ab,kw OR (Pharmacopuncture):ti,ab,kw OR (Acupuncture Treatment):ti,ab,kw OR (Acupuncture Treatments):ti,ab,kw OR (Treatment, Acupuncture):ti,ab,kw OR (Therapy, Acupuncture):ti,ab,kw OR (Pharmacoacupuncture Treatment):ti,ab,kw OR (Treatment, Pharmacoacupuncture):ti,ab,kw OR (Pharmacoacupuncture Therapy):ti,ab,kw OR (Therapy, Pharmacoacupuncture):ti,ab,kw OR (Acupotomy):ti,ab,kw OR (Acupotomies):ti,ab,kw OR (Acupunctures, Ear):ti,ab,kw OR (Ear Acupunctures):ti,ab,kw OR (Acupuncture, Auricular):ti,ab,kw OR (Acupunctures, Auricular):ti,ab,kw OR (Auricular Acupunctures):ti,ab,kw OR (Auricular Acupuncture):ti,ab,kw OR (Ear Acupuncture):ti,ab,kw OR (acupunc*):ti,ab,kw |
| #3:#1 OR #2 |
| #4:MeSH descriptor: [Drug Therapy] explode all trees |
| #5:(Drug Therapy):ti,ab,kw OR (drug therapy):ti,ab,kw OR (Chemotherapy, Adjuvant):ti,ab,kw OR (Antineoplastic Combined Chemotherapy Protocols):ti,ab,kw OR (Hyperthermic Intraperitoneal Chemotherapy):ti,ab,kw OR (Antineoplastic Agents):ti,ab,kw OR (Neoadjuvant Therapy):ti,ab,kw OR (Drug Therapy, Combination):ti,ab,kw OR (Chemotherapy):ti,ab,kw OR (Chemotherapies):ti,ab,kw OR (Pharmacotherapy):ti,ab,kw OR (Pharmacotherapies):ti,ab,kw OR (Therapy, Drug):ti,ab,kw OR (Drug Therapies):ti,ab,kw OR (Therapies, Drug):ti,ab,kw OR (pharmacologic therapy):ti,ab,kw OR (pharmacotherapy):ti,ab,kw OR (Chemotherapy):ti,ab,kw OR (Adjuvant Drug Therapy):ti,ab,kw OR (Drug Therapy, Adjuvant):ti,ab,kw OR (Adjuvant Chemotherapy):ti,ab,kw OR (Combined Antineoplastic Agents):ti,ab,kw OR (Antineoplastic Agents, Combined):ti,ab,kw OR (Agent, Combined Antineoplastic):ti,ab,kw OR (Cancer Chemotherapy Protocols):ti,ab,kw OR (Cancer Chemotherapy Protocol):ti,ab,kw OR (Chemotherapy Protocol, Cancer):ti,ab,kw OR (Chemotherapy Protocols, Cancer):ti,ab,kw OR (Protocol, Cancer Chemotherapy):ti,ab,kw OR (Protocols, Cancer Chemotherapy):ti,ab,kw OR (Chemotherapy Protocols, Antineoplastic):ti,ab,kw OR (Chemotherapy, Hyperthermic Intraperitoneal):ti,ab,kw OR (Intraperitoneal Chemotherapy, Hyperthermic):ti,ab,kw OR (Intraperitoneal Hyperthermic Chemotherapy):ti,ab,kw OR (Chemotherapy, Intraperitoneal Hyperthermic):ti,ab,kw |
| #6:#4 OR #5 |
| #7: MeSH descriptor: [Nausea] explode all trees |
| #8: MeSH descriptor: [Vomiting] explode all trees |
| #9:(Nausea):ti,ab,kw OR (Vomiting):ti,ab,kw OR (Emesis):ti,ab,kw |
| #10:#7 OR #8 OR #9 |
| #11: #3 AND #6 AND #10 |

| **CNKI** |
| --- |
| TKA%=('针刺' + '针灸' + '电针' + '耳针' + '针') AND TKA%=('化疗' + '癌' + '肿瘤') AND TKA%=('恶心' + '呕吐') AND TKA%=('临床' + '随机' + 'RCT' + '疗效') |

| **WanFang** |
| --- |
| (题名或关键词:(针刺 or 针 or 针灸 or 耳针 or 电针) or 摘要:(针刺 or 针 or 针灸 or 耳针 or 电针)) and (题名或关键词:(化疗 or 癌 or 肿瘤) or 摘要:(化疗 or 癌 or 肿瘤)) and (题名或关键词:(恶心 or 呕吐) or 摘要:(恶心 or 呕吐)) and (题名或关键词:(临床 or 疗效 or 随机 or 摘要:(临床 or 疗效 or 随机)) |

| **SinoMed** |
| --- |
| #1:("恶心"常用字段:智能])OR"呕吐"[常用字段:智能] |
| #2: ("癌"[常用字段:智能]) OR'肿瘤"[常用字段:智能] |
| #3: "化疗"[常用字段:智能] |
| #4:(#3)或(#2) |
| #5:(("针"[不加权:扩展] OR"皮内针疗法"[不加权:扩展) OR"头针疗法"[不加权:扩展] OR"手针疗法"[不加权:扩展] OR"耳针疗法"[不加权:扩展] OR"电针疗法"[不加权:扩展]) OR"针灸疗法"[不加权:扩展])OR"针刺"[不加权:扩展] |
| #6: "随机对照试验"[常用字段:智能] |
| #7:"随机"[常用字段:智能] OR"疗效"[常用字段:智能]OR"观察"[常用字段:智能] OR"临床"[常用字段:智能] |
| #8:(#7)或(#6) |
| #9:(#8)和(#5)和(#4)和(#1) |

**Table S3 Reasons for article exclusion after full-text reading**

| **ID** | **Author** | **Title** | | **Reason** |
| --- | --- | --- | --- | --- |
| 1 | W. Beate | | A randomised placebo-controlled pilot study of pericardium 6 acupressure and acupuncture as additive antiemetic therapy during chemotherapy in children and adolescents | Conference Abstract |
| 2 | C. I. Chi | | Acupuncture intervention time for nausea and vomiting caused by chemotherapy regimens including platinum the curative effect of impact assessment studies | protocol |
| 3 | C. I. Chi | | Study on the correlation between substance P and acupuncture treat delayed chemotherapy-incluced nausea and vomiting | protocol |
| 4 | C. T. Chi | | Prevention and treatment of chemotherapy-induced nausea and vomiting in cancer patients by ondansetron plus wrist-ankle acupuncture: a randomized controlled trial | protocol |
| 5 | ChiCtr | | Nursing effect of sputum acupuncture in the prevention and treatment of chemotherapy patients with nausea and vomiting | protocol |
| 6 | ChiCtr | | Acupuncture for the management of side effects caused by chemotherapy among I~ III period breast cancer patients: a randomized, controlled, blind, prospective clinical study | protocol |
| 7 | ChiCtr | | The efficacy and safety of auricular acupuncture versus electroacupuncture in ameliorating chemotherapy-induced nausea and vomiting among patients receiving cisplatin-based regimens | protocol |
| 8 | ChiCtr | | Effect of acupuncture on nausea and vomiting induced by cisplatin chemotherapy during pelvic external irradiation for gynecological tumors | protocol |
| 9 | ChiCtr | | Clinical study on acupuncture intervention of 'Siguan point' on nausea and vomiting induced by high emetic chemotherapy in patients with breast cancer | protocol |
| 10 | ChiCtr | | Observation on the curative effect of acupuncture on gynecological tumor postoperative adverse reactions caused by chemotherapy | protocol |
| 11 | ChiCtr | | A randomized controlled trial on the effectiveness and safety of acupuncture in the treatment of gastrointestinal dysfunction after chemotherapy for colorectal cancer | protocol |
| 12 | ChiCtr | | Effect of different methods of auricular point stimulation on chemotherapy-induced nausea and vomiting and appetite in patients receiving platinum-based chemotherapy | protocol |
| 13 | ChiCtr | | Electroacupuncture intervention for gastrointestinal dysfunction after chemotherapy in colorectal cancer: a randomized controlled trial | protocol |
| 14 | Nct | | Adjuvant Acupuncture Care for Breast Cancer Patients Experiencing Side Effects From Chemotherapy | protocol |
| 15 | Nct | | Acupuncture Point Stimulation for Treatment of Chemotherapy Nausea and Vomiting | protocol |
| 16 | Nct | | The Efficacy of Acupuncture in Treating Chemotherapy Side Effects in Breast Cancer Patients | protocol |
| 17 | Nct | | Acupuncture for Chemical Therapy Induced Nausea and Vomiting: a Cross-over Trail | protocol |
| 18 | Nct | | Acupuncture for Chemotherapy-Induced Nausea and Vomiting | protocol |
| 19 | Nct | | The Effect of Acupuncture in Chemotherapy-induced Nausea and Vomiting | protocol |
| 20 | Nct | | The Effect and Mechanism of Electroacupuncture on Acute Chemotherapy-induced Nausea and Vomiting | protocol |
| 21 | Nct | | Auriculotherapy and Acupuncture's Treatment for Chemotherapy-induced Nausea and Vomiting (CINV) | protocol |
| 22 | Nct | | Acupuncture for Chemical Therapy Induced Nausea and Vomiting | protocol |
| 23 | Nct | | Effects of Aqualief® in Patients With Xerostomia as Consequence of Radiotherapy for Head and Neck Cancer | protocol |
| 24 | Nct | | Is Acupuncture Able to Reduce Nausea and Vomiting in the Terminal Ill Patient | protocol |
| 25 | Nct | | Personalized Electroacupuncture Treatment for Chemotherapy-induced Nausea and Vomiting in Breast Cancer (PET) | protocol |
| 26 | Nct | | Efficacy of Ear Acupuncture in Preventing Chemotherapy Induced Nausea and Vomiting in Cancer Patients | protocol |
| 27 | Nct | | Electroacupuncture for the Prevention of Chemotherapy-induced Nausea and Vomiting in Patients With Breast Cancer | protocol |
| 28 | J. W. Dundee | | Acupuncture prophylaxis of cancer chemotherapy-induced sickness | outcomes don't match |
| 29 | X. Zhang | | He research about mechanism of wrist electric acupuncture apparatus stimulation on CINV | outcomes don't match |
| 30 | J. W. Dundee | | Randomised comparison of the antiemetic effects of metoclopramide and electro-acupuncture in cancer chemotherapy | protocol |
| 31 | S. Gottschling | | Acupuncture to alleviate chemotherapy-induced nausea and vomiting in pediatric oncology - a randomized multicenter crossover pilot trial | outcomes don't match |
| 32 | M. Guo | | Effect of intradermal needle therapy at combined acupoints on patients’ gastrointestinal function following surgery for gastrointestinal tumors | outcomes don't match |
| 33 | Isrctn | | The effect of acupuncture in the prevention and treatment of chemotherapy-induced nausea and vomiting in patients with advanced cancer | protocol |
| 34 | Nct | | Effect of different methods of auricular point stimulation on chemotherapy-induced nausea and vomiting and appetite in patients receiving platinum-based chemotherapy | protocol |
| 35 | Nct | | Effect of acupuncture on chemotherapy-induced nausea and vomiting | protocol |
| 36 | T. K. Reindl | | Acupuncture against chemotherapy-induced nausea and vomiting in pediatric oncology. Interim results of a multicenter crossover study | protocol |
| 37 | M. Y. Liu | | The efficacy and safety of auricular acupuncture versus electroacupuncture in ameliorating chemotherapy-induced nausea and vomiting among patients receiving cisplatin-based regimens | protocol |
| 38 | M. Eghbali, S. | | Use of ear acupressure as a strategy to relieve nausea and vomiting caused by chemotherapy in patients with breast cancer | Conference Abstract |
| 39 | A. Genç, G | | The efficiency of the acupressure in prevention of the chemotherapy-induced nausea and vomiting | Interventions do not meet |
| 40 | E. Jones, S | | Acupressure for chemotherapy-associated nausea and vomiting in children | Interventions do not meet |
| 41 | R. Kaur, D | | An experimental study to assess effectiveness of acupressure on relief of chemotherapy induced nausea and vomiting among cancer patients in selected hospital, Punjab | Interventions do not meet |
| 42 | J. Klein and P. Griffiths | | Acupressure for nausea and vomiting in cancer patients receiving chemotherapy | Interventions do not meet |
| 43 | Kong C | | Curative effect of auricular point sticking for SOX chemotherapy-induced nausea, vomiting and diarrhea | protocol |
| 44 | L. Lao | | A controlled study using acupuncture as an adjuvant to treat chemotherapy-induced nausea and vomiting | protocol |
| 45 | L. X. Lao | | The effect of electroacupuncture as an adjunct on cyclophosphamide-induced emesis in ferrets | animal experimentation |
| 46 | D. Lu | | Electrothermal acupuncture in the prevention and treatment of chemotherapy-induced nausea and vomiting: a randomized controlled trial | protocol |
| 47 | C. Maeng | | Additional effect of acupuncture on standard anti-emetic prophylaxis in patients under highly emetogenic chemotherapy (KHMC HO-01) | Conference Abstract |
| 48 | C. McKeon | | Electroacupuncture vs sham electroacupuncture vs standard care for chemotherapy induced nausea and vomiting-a pilot study | Conference Abstract |
| 49 | A. Molassiotis | | The effectiveness of acupressure for the control and management of chemotherapy-related acute and delayed nausea: a randomized controlled trial | Interventions do not meet |
| 50 | B. Oh | | Randomized, controlled pilot trial of electro-acupuncture for nausea, Vomiting and myelosuppression in women receiving adjuvant chemotherapy for early breast cancer | Conference Abstract |
| 51 | M. J. Paech | | The use of acupressure to prevent nausea and vomiting in labour and delivery: A randomised, double-blind, placebo-controlled study evaluating antiemetic efficacy of the Pressure Right™ acupressure wristband | Interventions do not meet |
| 52 | P. Perkins | | Does acupressure help reduce nausea and vomiting in palliative care patients? A double blind randomised controlled trial | Interventions do not meet |
| 53 | J. A. Roscoe | | The efficacy of acupressure and acustimulation wrist bands for the relief of chemotherapy-induced nausea and vomiting: A University of Rochester Cancer Center Community Clinical Oncology Program Multicenter study | Interventions do not meet |
| 54 | S. M. Sagar | | Acupuncture as an Evidence-Based Option for Symptom Control in Cancer Patients | Not RCT |
| 55 | H. S. Sallam | | Electroacupuncture via Chronically Implanted Electrodes: Potential Treatment for Chemotherapy-Induced Delayed Emesis | animal experimentation |
| 56 | C. H. Shen and L. Y. Yang | | The Effects of Acupressure on Meridian Energy as well as Nausea and Vomiting in Lung Cancer Patients Receiving Chemotherapy | Interventions do not meet |
| 57 | Y. H. Shin | | Effect of acupressure on nausea and vomiting during chemotherapy cycle for Korean postoperative stomach cancer patients | Interventions do not meet |
| 58 | P. Siegel, N. F | | Acupuncture for cancer patients undergoing chemotherapy in a Brazilian hospital-An exploratory study | Not RCT |
| 59 | E. E. Suh | | The effects of P6 acupressure and nurse-provided counseling on chemotherapy-induced nausea and vomiting in patients with breast cancer | Interventions do not meet |
| 60 | Tctr | | Efficacy of acupuncture in prevention delayed chemotherapy induced nausea and vomiting in gynecologic cancer patients | duplicate reserch |
| 61 | D. T. C. Tony | | The effectiveness of pericardium 6 (PC6) and stomach 36 (ST36) point: Acupressure in the management of acute type nausea and vomiting, anxiety and quality of life of patients undergoing chemotherapy | Interventions do not meet |
| 62 | B. Wicaksono, F. I. Hadian | | The effect of digital acupressure on chemotherapy-induced nausea in Indonesian patients with stages III-IV breast cancer | Interventions do not meet |
| 63 | E. T. Xia Yue-Shan | | Acupuncture Plus Ear-Points Press in Preventing Vomiting Induced by Chemotherapy with Cisplatin | Interventions do not meet |
| 64 | X. Zhang and Y. Fan | | The research about the wrist electric acupuncture apparatus stimulation on CINV | Conference Abstract |
| 65 | Qingchun Zhao | | To Explore the Effects of Acupuncture and Medical Treatment at Different Times on the Gastrointestinal Reaction and White Blood Cell Count of Patients with Lung Cancer Chemotherapy | Withdraw |
| 66 | X. Chenbing | | Effect of ginger and P6 acupressure on chemotherapy-induced nausea and vomiting: a randomized controlled study | Interventions do not meet |
| 67 | Wang Wangsheng | | Observation on the efficacy of auricular acupoint bean burrowing on the improvement of nausea and vomiting after chemotherapy for gastric cancer | Non-highly emetogenic chemotherapy |
| 68 | Zhu Chunning | | Clinical study on the prevention of nausea and vomiting due to tumor chemotherapy by burying beans in ear acupuncture points | Non-highly emetogenic chemotherapy |
| 69 | Gao Pan | | Effects of auricular pressure bean combined with psychological nursing measures on patients with nausea and vomiting in adenocarcinoma chemotherapy | Interventions do not meet |
| 70 | Xu Lei | | Comparison of the efficacy of moxibustion and acupuncture gastrofacial on gastrointestinal reactions after chemotherapy for digestive tract tumors | Interventions do not meet |
| 71 | Zheng Qimei | | Interventional effects of intradermal acupuncture and acupoint submergence on chemotherapy-induced nausea and vomiting in patients with malignant hematologic diseases | Interventions do not meet |
| 72 | Fei Juan | | Comparison of the efficacy of Ziwu Liuzhi selective time acupoint application and combined microneedle acupuncture in preventing and treating chemotherapy-induced gastrointestinal reactions | Interventions do not meet |
| 73 | Huang Yinfeng | | Treatment of chemotherapy-related nausea and vomiting by lifting needle combined with gastroretentive acupoint injection | Interventions do not meet |
| 74 | Wu Tao | | Application effect of auricular acupressure with acupoint moxibustion in preventing delayed vomiting in breast cancer chemotherapy patients | Interventions do not meet |
| 75 | Wen Nini | | Effectiveness of auricular acupuncture point burying beans combined with spacer moxibustion in preventing nausea and vomiting caused by platinum-based chemotherapy | Interventions do not meet |
| 76 | Zhou Xiaoqiong | | The efficacy of acupoint application combined with electroacupuncture in the prevention and treatment of nausea and vomiting in patients undergoing chemotherapy with cisplatin-containing regimen for lung cancer | Interventions do not meet |
| 77 | Kong Chen | | Efficacy of auricular acupressure in the treatment of nausea, vomiting and diarrhea associated with SOX chemotherapy regimen for gastric cancer | Interventions do not meet |
| 78 | Xu Jinglan | | Clinical observation of auricular acupuncture point burying bean combined with Neiguan point burying needle in the prevention and treatment of nausea and vomiting in breast cancer chemotherapy | Not RCT |
| 79 | Rong Huanlan | | Clinical nursing study of acupoint burrowing combined with massage for prevention and treatment of post-chemotherapy gastrointestinal adverse reactions in patients with leukemia | Non-highly emetogenic chemotherapy |
| 80 | Feng Xiumei | | Clinical efficacy of acupuncture combined with acupressure in the treatment of gastrointestinal reactions to chemotherapy in breast cancer patients | Non-highly emetogenic chemotherapy |
| 81 | Liu Meng | | Efficacy of acupuncture treatment on gastrointestinal toxic reactions in patients with chemotherapy for colorectal cancer | Non-highly emetogenic chemotherapy |
| 82 | Xu Lin | | Effectiveness observation of acupuncture combined with granisetron hydrochloride in treating gastrointestinal reactions in patients with chemotherapy for gastrointestinal tumors | Non-highly emetogenic chemotherapy |
| 83 | Yang Jinliang | | Clinical Study on the Mechanism of Combined Acupuncture and Medication in Treating Gastrointestinal Symptoms in Tumor Patients After Chemotherapy | Non-highly emetogenic chemotherapy |
| 84 | Lu Aiyu | | Observation on the Efficacy of Auricular Acupoint Embedding in Preventing Vomiting in Breast Cancer Patients Undergoing Chemotherapy | Not RCT |
| 85 | Gao Yinyin | | Application of Auricular Acupressure in Relieving Nausea and Vomiting in Pediatric Leukemia Patients Undergoing Chemotherapy | Non-highly emetogenic chemotherapy |
| 86 | Su Boshen | | Efficacy Observation of Auricular Acupressure Combined with Acupoint Application in Preventing Chemotherapy-Induced Nausea and Vomiting | control group does not match |
| 87 | Liu Shuyu | | Efficacy Observation of Neiguan Acupoint Ginger Application Combined with Ear Acupuncture in Treating Nausea, Vomiting, and Retching in Tumor Patients After Chemotherapy | Interventions do not meet |
| 88 | Li Zizhuo | | Therapeutic Effects of Acupuncture on Malignant Tumors After Chemotherapy | Non-highly emetogenic chemotherapy |
| 89 | Chen Meng | | Efficacy Observation of Acupuncture Combined with Medication in Treating Gastrointestinal Reactions in Gastrointestinal Tumor Patients Undergoing Chemotherapy | Non-highly emetogenic chemotherapy |
| 90 | Lai Milin | | Efficacy Observation of Bo's Abdominal Acupuncture in Treating Nausea and Vomiting in Breast Cancer Patients After Chemotherapy | Non-highly emetogenic chemotherapy |
| 91 | Zhao Chunlei | | The Impact of Auricular Acupressure on Gastrointestinal Reactions in Cancer Patients During Chemotherapy | Non-highly emetogenic chemotherapy |
| 92 | Liang Xiaoning | | Efficacy Observation of Modified Ginger Heart-Draining Decoction Combined with Acupuncture in Preventing Vomiting After Chemotherapy | Non-highly emetogenic chemotherapy |
| 93 | Liu Yan | | The Effect of Early Auricular Acupressure on Nausea and Vomiting in Colorectal Cancer Patients After Postoperative Chemotherapy | Non-highly emetogenic chemotherapy |
| 94 | Wang Line | | Application of Auricular Acupoint Embedding in Preventing Chemotherapy-Induced Nausea and Vomiting in Tumor Patients | control group does not match |
| 95 | Guo Chunmei | | Clinical Observation of Auricular Acupressure Combined with Nursing Intervention in Preventing Nausea and Vomiting Induced by Gastric Cancer Chemotherapy | Non-highly emetogenic chemotherapy |
| 96 | Liu Yugan | | Efficacy Observation of Auricular Acupressure Combined with Zusanli Acupoint Injection in Preventing Chemotherapy-Induced Nausea and Vomiting | Non-highly emetogenic chemotherapy |
| 97 | Lu Chunling | | Observation and Nursing of Auricular Acupressure in Preventing Chemotherapy-Induced Nausea and Vomiting | Non-highly emetogenic chemotherapy |
| 98 | Hong Ri | | Efficacy Observation of Auricular Acupressure in Preventing Vomiting in Breast Cancer Patients During Chemotherapy | control group does not match |
| 99 | Zhao Xu | | Efficacy Observation of Auricular Acupressure Combined with Jiangxia Powder Umbilical Therapy in Preventing Nausea and Vomiting Induced by Primary Gastric Cancer Chemotherapy | Non-highly emetogenic chemotherapy |
| 100 | Yan Yujing | | Clinical Observation of Acupuncture Combined with Moxibustion in Preventing Chemotherapy-Induced Nausea and Vomiting | Non-highly emetogenic chemotherapy |
| 101 | Gao Haili | | Efficacy Observation of "Xiaqi Decoction" Combined with Auricular Acupressure in Treating Vomiting After Chemotherapy | control group does not match |
| 102 | Zhang Yongmei | | Efficacy Observation of Auricular Acupuncture in Preventing Nausea and Vomiting Induced by Breast Cancer Chemotherapy: 40 Cases | Non-highly emetogenic chemotherapy |
| 103 | Yang Yan | | Auricular Acupressure in Preventing Vomiting After Chemotherapy for Malignant Hematological Diseases: 38 Cases | Non-highly emetogenic chemotherapy |
| 104 | Xing Jinyun | | Efficacy Observation of Harmonizing Stomach and Lowering Rebellious Qi Acupuncture in Treating Cisplatin-Induced Nausea and Vomiting | Not RCT |
| 105 | Peng Meiping | | Efficacy Observation of Acupoint Therapy on Nausea and Vomiting Induced by Breast Cancer Chemotherapy | Non-highly emetogenic chemotherapy |
| 106 | Cui Junling | | Efficacy Observation of Acupuncture in Preventing Nausea and Vomiting After Chemotherapy for Malignant Tumors | Non-highly emetogenic chemotherapy |
| 107 | Huang Yinfeng | | Clinical Study of Acupuncture Combined with Acupoint Injection in Preventing Chemotherapy-Induced Vomiting | Non-highly emetogenic chemotherapy |
| 108 | Shen Baoyu | | Clinical Observation of Zusanli and Neiguan Acupuncture in Treating Gastrointestinal Reactions in Gastrointestinal Tumor Patients After Chemotherapy | Non-highly emetogenic chemotherapy |
| 109 | Fan Yashuo | | Acupuncture and Acupoint Block Therapy for Chemotherapy-Induced Vomiting | control group does not match |
| 110 | Han Jianhong | | Acupuncture in Treating Gastrointestinal Side Effects After Chemotherapy | Non-highly emetogenic chemotherapy |
| 111 | Zhang Wei | | Clinical Observation of Acupuncture in Treating Nausea and Vomiting Induced by Chemotherapy and Radiotherapy | control group does not match |
| 112 | Fu Qiang | | Efficacy Analysis of Ondansetron Combined with Acupuncture in Treating Chemotherapy-Induced Nausea and Vomiting | Non-highly emetogenic chemotherapy |
| 113 | Que Tiesheng | | Clinical Observation of Acupuncture in Preventing Cisplatin-Induced Vomiting | Non-highly emetogenic chemotherapy |
| 114 | Shen Guowei | | The Impact of Acupuncture on Gastric Dynamics in Chemotherapy Patients with Vomiting | outcomes don't match |
| 115 | Fan Ruzhen | | Acupuncture Combined with Psychological Counseling for Gastrointestinal Side Effects Induced by Chemotherapy | Non-highly emetogenic chemotherapy |
| 116 | Li Dongfang | | Acupuncture in Preventing Chemotherapy-Induced Vomiting: 34 Cases | Not RCT |
| 117 | Huang Ting | | Treatment of Chemotherapy-Induced Vomiting with Shudan Acupuncture: 28 Cases | Not RCT |
| 118 | Zhang Bo | | Efficacy Observation of Acupuncture in Treating Gastrointestinal Reactions Induced by Chemotherapy | Non-highly emetogenic chemotherapy |
| 119 | Zhou Yun | | Efficacy Observation of Acupuncture in Reducing Adverse Reactions in Liver Cancer Patients Undergoing Interventional Chemotherapy | Non-highly emetogenic chemotherapy |
| 120 | Wang Shouzhang | | Clinical Study on Acupuncture Combined with Acupoint Injection in Preventing Chemotherapy-Induced Vomiting: 45 Cases | Not RCT |
| 121 | Gong Hongjie | | Clinical Observation of Traditional Chinese Medicine Combined with Acupuncture in Treating Chemotherapy-Induced Vomiting | control group does not match |
| 122 | Wang Guiping | | The Effect of Different Timing of Acupuncture on Postoperative Nausea and Vomiting in Rectal Cancer Patients: Measurement of Plasma Gastrin Concentration | duplicate reserch |
| 123 | Yao Xinyu | | Efficacy Observation of Auricular Acupressure as an Adjunctive Therapy for Nausea and Vomiting in Breast Cancer Patients After Chemotherapy | Not RCT |
| 124 | Wang Yi | | Auricular Acupressure Combined with Ginger Moxibustion in Preventing Nausea and Vomiting After Chemotherapy: 60 Cases | Non-highly emetogenic chemotherapy |
| 125 | Wang Hong | | The Impact of Neiguan Acupoint Intradermal Needle Embedding on Nausea and Vomiting in Breast Cancer Patients Undergoing Chemotherapy | Non-highly emetogenic chemotherapy |
| 126 | Zheng Wei | | Auricular Acupressure Combined with Acupoint Massage in Preventing Nausea and Vomiting in Breast Cancer Patients After Chemotherapy: 30 Cases | control group does not match |
| 127 | Qin Hongyu | | Efficacy Observation of Auricular Acupressure Combined with 5-HT3 Receptor Antagonists in Treating Chemotherapy-Induced Vomiting | Non-highly emetogenic chemotherapy |
| 128 | Ye Yufei | | Clinical Observation of Acupoint Injection Combined with Auricular Acupressure in Preventing Vomiting in Malignant Tumor Patients Undergoing Chemotherapy | Non-highly emetogenic chemotherapy |
| 129 | Ding Zhifang | | Clinical Observation of Antiemetic Patch Combined with Acupuncture in Treating Chemotherapy-Related Nausea and Vomiting | Not RCT |
| 130 | Yang Haining | | Clinical Observation of Acupuncture Combined with Auricular Acupressure in Treating Chemotherapy-Induced Vomiting: 60 Cases | Non-highly emetogenic chemotherapy |
| 131 | Tai Jie | | The Impact of Different Acupuncture Methods at Zusanli on Gastric Dynamics in Chemotherapy Patients with Vomiting | Non-highly emetogenic chemotherapy |
| 132 | Shen Guowei | | Electroacupuncture at Zusanli in Treating Nausea and Vomiting Induced by Chemotherapy for Malignant Tumors: A Multicenter Randomized Controlled Study | control group does not match |
| 133 | Yang Yan | | Acupuncture Combined with Herbal Pack in Preventing Vomiting in Breast Cancer Patients Undergoing Chemotherapy: 68 Cases | Non-highly emetogenic chemotherapy |
| 134 | Wang Cong | | Efficacy Observation of Acupuncture Combined with Acupoint Injection in Treating Chemotherapy-Induced Vomiting | control group does not match |
| 135 | Zhao Hexin | | Efficacy Observation of Auricular Acupressure Combined with Acupuncture in Preventing Nausea and Vomiting in Chemotherapy Patients | control group does not match |
| 136 | Cao Cuiqin | | Acupuncture Combined with Auricular Acupressure in Treating Chemotherapy-Induced Vomiting: 36 Cases | Non-highly emetogenic chemotherapy |
| 137 | Tai Jie | | Clinical Observation of Auricular Acupressure in Relieving Vomiting in Cancer Patients After Chemotherapy: 80 Cases | control group does not match |
| 138 | Wang Bianli | | Efficacy Observation of Acupuncture at Gongsun and Neiguan in Preventing Chemotherapy-Induced Vomiting | Non-highly emetogenic chemotherapy |
| 139 | Zhou Li | | Acupuncture and Acupressure in Treating Chemotherapy-Induced Nausea and Vomiting | Not RCT |
| 140 | Zhang Jing | | Clinical Observation of Acupuncture in Treating Chemotherapy-Induced Vomiting | Not RCT |
| 141 | Chen Jie | | Combined Acupuncture and Medication in Treating Gastrointestinal Reactions After Chemotherapy: 23 Cases | Non-highly emetogenic chemotherapy |
| 142 | Li Ying | | Clinical Observation of Danshen Powder Injection in Preventing Toxic Side Effects of Tumor Chemotherapy | Non-highly emetogenic chemotherapy |
| 143 | Su Zhong | | Application of Auricular Acupressure Combined with Ondansetron in Chemotherapy-Induced Nausea and Vomiting | Interventions do not meet |
| 144 | Ding Yaqin | | Application of Acupuncture in Chemotherapy-Induced Vomiting | Non-highly emetogenic chemotherapy |
| 145 | Yu Cuizhi | | Clinical Observation of Acupuncture Combined with Auricular Acupressure in Treating Chemotherapy-Induced Vomiting: 60 Cases | duplicate reserch |
| 146 | Tai Jie | | The Impact of Different Acupuncture Methods at Zusanli on Gastric Dynamics in Chemotherapy Patients with Vomiting | Non-highly emetogenic chemotherapy |
| 147 | Shen Guowei | | Comparative Efficacy of Moxibustion and Acupuncture with Metoclopramide on Gastrointestinal Reactions in Digestive Tract Tumor Patients After Chemotherapy | Interventions do not meet |
| 148 | Zhang Zhijun | | The Impact of Aromatherapy Combined with Acupoint Application Nursing on Nausea and Vomiting Induced by Chemotherapy in Gastrointestinal Tumor Patients | Non-highly emetogenic chemotherapy |
| 149 | Zhang Dongmei | | Intervention Effects of Intradermal Needle and Acupoint Catgut Embedding on Nausea and Vomiting Induced by Chemotherapy in Malignant Hematological Disease Patients | Non-highly emetogenic chemotherapy |
| 150 | Zheng Qimei | | Clinical Study on Warm Needling Combined with Acupoint Application and Medication in Treating Chemotherapy-Related Nausea and Vomiting in Breast Cancer Patients | Non-highly emetogenic chemotherapy |
| 151 | Wei Liuhong | | Comparative Efficacy of Time-Based Acupoint Application and Combined Micro-Acupuncture in Preventing Gastrointestinal Reactions Induced by Chemotherapy | Non-highly emetogenic chemotherapy |
| 152 | Fei Juan | | Efficacy of Acupoint Application Therapy in Treating Nausea and Vomiting in Breast Cancer Patients After Chemotherapy | Non-highly emetogenic chemotherapy |
| 153 | Chen Ying | | Clinical Study on Ondansetron Combined with Warm Acupuncture in Treating Vomiting Induced by Tumor Chemotherapy | Not RCT |
| 154 | Wu Chunyu | | Efficacy and Nursing Study of Auricular Acupressure Combined with Neiguan and Zusanli Micro-Acupuncture in Preventing Nausea and Vomiting Induced by Colorectal Cancer Chemotherapy | Non-highly emetogenic chemotherapy |
| 155 | Fei Juan | | The Impact of Abdominal Acupuncture Intervention on Quality of Life and Gastrointestinal Reactions in Malignant Bone Tumor Patients Undergoing Chemotherapy | Non-highly emetogenic chemotherapy |
| 156 | Si Rong | | Clinical Efficacy of Intradermal Needle in Treating Nausea and Vomiting Induced by Malignant Tumor Chemotherapy | Non-highly emetogenic chemotherapy |
| 157 | Deng Bei | | Clinical Observation of Press Needle Embedding Combined with Moxibustion in Treating Spleen-Stomach Deficiency Cold Type Vomiting in Breast Cancer Patients After Chemotherapy | Non-highly emetogenic chemotherapy |
| 158 | Jiang Wenting | | Efficacy Analysis of Body Acupoints Combined with Auricular Press Needle Embedding in Preventing Nausea and Vomiting During Malignant Tumor Chemotherapy | Non-highly emetogenic chemotherapy |
| 159 | Sun Xiaoli | | Efficacy Observation of Press Needle Embedding Combined with Antiemetic Patch in Treating Vomiting in Lung Cancer Patients After Postoperative Chemotherapy | Non-highly emetogenic chemotherapy |
| 160 | Wang Jinguo | | Clinical Study on Acupuncture at Weitong Acupoint and Neiguan Acupoint in Treating Nausea and Vomiting Induced by Chemotherapy in NSCLC Patients: 30 Cases | Non-highly emetogenic chemotherapy |
| 161 | Xuan Jing | | Observation and Analysis of the Efficacy and Nursing Measures of Auricular Acupressure in Preventing Gastrointestinal Reactions in Tumor Patients After Chemotherapy | Non-highly emetogenic chemotherapy |
| 162 | Zhou Li | | Nursing Efficacy Study of Press Needle Combined with Box Moxibustion in Preventing Nausea and Vomiting During Breast Cancer Chemotherapy | Non-highly emetogenic chemotherapy |
| 163 | Zheng Xiaoxiao | | Clinical Efficacy of Ginger Juice Oral Administration Combined with Auricular Acupressure in Improving Cisplatin-Induced Nausea and Vomiting | Non-highly emetogenic chemotherapy |
| 164 | Zhou Jialing | | Intervention Effect of Auricular Acupressure Combined with Cognitive Behavioral Therapy on Chemotherapy-Induced Nausea and Vomiting | control group does not match |
| 165 | Feng Lixia | | Efficacy Observation of Acupuncture Combined with Traditional Chinese Medicine in Treating Nausea and Vomiting in Tumor Patients After Chemotherapy | control group does not match |
| 166 | Meng Zhaoying | | Acupuncture Treatment of Nausea and Vomiting in Tumor Patients After Chemotherapy | control group does not match |
| 167 | Meng Zhaoying | | Efficacy Observation of Auricular Acupressure in Improving Gastrointestinal Reactions in Colorectal Cancer Patients After Postoperative Chemotherapy | Non-highly emetogenic chemotherapy |
| 168 | Xu Zhihui | | Efficacy Observation of Auricular Acupressure Combined with Ginger Moxibustion in Preventing Gastrointestinal Reactions Induced by Gynecological Malignant Tumor Chemotherapy | control group does not match |
| 169 | Wang Xiying | | Efficacy Observation of Auricular Acupressure Combined with Acupoint Application in Preventing Chemotherapy-Induced Nausea and Vomiting | Non-highly emetogenic chemotherapy |
| 170 | Su Boshen | | Clinical Efficacy Observation of Auricular Acupressure Combined with Nursing Intervention in Preventing Nausea and Vomiting Induced by Gastric Cancer Chemotherapy | Non-highly emetogenic chemotherapy |
| 171 | Zhu Xiaorui | | The Impact of Electroacupuncture at Neiguan and Jianshi Acupoints on Chemotherapy-Induced Nausea and Vomiting | Non-highly emetogenic chemotherapy |
| 172 | Zhang Xing | | Efficacy of Auricular Acupressure and Ondansetron in Preventing Nausea and Vomiting After Chemotherapy | control group does not match |
| 173 | Xu Jun | | Study on Auricular Acupressure Combined with Acupoint Finger Pressure in Reducing Chemotherapy-Related Nausea and Vomiting in Gastric Cancer Patients | control group does not match |
| 174 | Hou Qingmei | | Application of Auricular Acupressure in Preventing Chemotherapy-Induced Nausea and Vomiting in Tumor Patients | control group does not match |
| 175 | Wang Line | | Clinical Efficacy Observation of Pulse-Based Acupuncture Combined with Traditional Chinese Medicine in Treating Chemotherapy-Related Toxicity in Colorectal Cancer Patients | Not RCT |
| 176 | Gao Qin | | The Impact of Acupoint Catgut Embedding on Chemotherapy-Related Nausea and Vomiting in Lung Cancer Patients | Non-highly emetogenic chemotherapy |
| 177 | Xu Ran | | Phase III Clinical Study on the Effectiveness and Safety of Acupuncture Combined with Conventional Antiemetic Compared to Conventional Antiemetic in Preventing CINV in Lung Cancer Patients Undergoing Highly Emetogenic Chemotherapy | Interventions do not meet |
| 178 | Yan Bingchuan | | Efficacy of Modified Pingwei Powder Combined with Fire Needle in Treating Gastrointestinal Reactions Induced by Chemotherapy in Rectal Cancer Patients | control group does not match |
| 179 | Ma Hongyu | | Clinical Observation on the Efficacy of Traditional Chinese Medicine Diet Combined with Auricular Acupressure as an Adjunctive Therapy in Controlling Nausea and Vomiting in Tumor Patients Undergoing Chemotherapy | control group does not match |
| 180 | Shen Pan | | The Impact of Auricular Acupressure on Nausea, Vomiting, and Quality of Life in Gastric Cancer Patients Undergoing Chemotherapy | Non-highly emetogenic chemotherapy |
| 181 | Liu Hong | | Clinical Study on Oral Traditional Chinese Medicine Combined with Acupuncture in Treating Vomiting (Spleen-Stomach Deficiency Type) Induced by Postoperative Chemotherapy in Breast Cancer Patients | control group does not match |
| 182 | Wang Haitao | | Nursing Efficacy Study of Press Needle Combined with Box Moxibustion in Preventing Nausea and Vomiting During Breast Cancer Chemotherapy | control group does not match |
| 183 | Liu Jie | | Clinical Observation of Press Needle Embedding Combined with Tropisetron in Relieving Nausea and Vomiting in Colorectal Cancer Patients Undergoing Chemotherapy | control group does not match |
| 184 | Chen Xiaoqiong | | The Impact of Acupuncture Combined with Thunder-Fire Moxibustion on Chemotherapy-Related Nausea and Vomiting | control group does not match |
| 185 | Liao Zilong | | Clinical Observation of Auricular Acupressure in Improving Gastrointestinal Reactions in Colorectal Cancer Patients After Postoperative Chemotherapy | control group does not match |
| 186 | Wang Xiaoying | | Efficacy Observation of Auricular Acupressure Combined with Acupoint Injection in Preventing Gastrointestinal Reactions in Chemotherapy Patients | Non-highly emetogenic chemotherapy |
| 187 | Li Yanping | | Clinical Observation of Acupuncture in Treating Chemotherapy-Induced Vomiting: 56 Cases | Non-highly emetogenic chemotherapy |
| 188 | Sun Jin | | Acupuncture in Preventing Nausea and Vomiting After Tumor Chemotherapy: 138 Cases | control group does not match |
| 189 | Zhi Ming | | Clinical Study on Combined Acupuncture and Medication in Treating Vomiting After Chemotherapy | Non-highly emetogenic chemotherapy |
| 190 | Yang Zhiyong | | Nursing Efficacy Observation of Press Needle Embedding in Relieving Nausea and Vomiting in Colorectal Cancer Patients After Postoperative Chemotherapy | control group does not match |
| 191 | Qian Chao | | The Impact of Auricular Acupressure Combined with Psychological Nursing on Nausea and Vomiting in Breast Cancer Patients Undergoing Chemotherapy | control group does not match |
| 192 | Gao Pan | | Clinical Study on Time-Based Acupuncture Combined with Tropisetron in Preventing Platinum-Induced Nausea and Vomiting in Gastric Cancer Patients | control group does not match |
| 193 | Yang Ling | | Clinical Study on Different Acupuncture Methods in Treating Chemotherapy-Induced Nausea and Vomiting | Non-highly emetogenic chemotherapy |
| 194 | Pu Zhongjian | | Prospective Randomized Controlled Study on "Lao Shi Zhen" in Preventing Chemotherapy-Related Nausea and Vomiting in Breast Cancer Patients | Non-highly emetogenic chemotherapy |
| 195 | He Peishan | | Clinical Study on "Wei San Zhen" Acupuncture Combined with Herbal Ointment Massage in Preventing Chemotherapy-Related Nausea and Vomiting | Non-highly emetogenic chemotherapy |
| 196 | Dang Yuanyuan | | The Impact of Time-Based Acupuncture on Gastrointestinal Reactions in Colon Cancer Patients Undergoing Postoperative Intraperitoneal Hyperthermic Chemotherapy | Non-highly emetogenic chemotherapy |
| 197 | Gu Xuying | | Efficacy Study of Time-Based Wrist-Ankle Acupuncture in Preventing Chemotherapy-Induced Nausea and Vomiting | Interventions do not meet |
| 198 | Jiang Xiaofang | | Clinical Study on Time-Based Acupuncture Combined with Press Needle in Treating Vomiting in Tumor Patients After Chemotherapy | Interventions do not meet |
| 199 | Ji Xiuling | | Efficacy Observation of Traditional Chinese Medicine Diet Combined with Auricular Acupressure as an Adjunctive Therapy in Controlling Nausea and Vomiting in Tumor Patients Undergoing Chemotherapy | Non-highly emetogenic chemotherapy |
| 200 | Huang Yurong | | The Impact of Press Needle Embedding Combined with Comprehensive Nursing on Nausea and Vomiting in Gastrointestinal Tumor Patients After Chemotherapy | Non-highly emetogenic chemotherapy |
| 201 | Xie Jing | | Clinical Observation of Antiemetic Patch Combined with Acupuncture in Treating Chemotherapy-Related Nausea and Vomiting | Non-highly emetogenic chemotherapy |
| 202 | Yang Haining | | Clinical Analysis of Acupuncture in Treating Chemotherapy-Related Nausea and Vomiting | Non-highly emetogenic chemotherapy |
| 203 | Zhang Min | | Clinical Exploration of Acupuncture in Treating Vomiting in Tumor Patients After Chemotherapy | Non-highly emetogenic chemotherapy |
| 204 | Yan Jihong | | Acupuncture in Treating Vomiting in Tumor Patients After Chemotherapy and Its Impact on Serum GAS and MTL Levels | Non-highly emetogenic chemotherapy |
| 205 | Sun Xuliang | | The Impact of Ginger Moxibustion Combined with Press Needle on Gastrointestinal Reactions in Malignant Tumor Patients Undergoing Chemotherapy | Non-highly emetogenic chemotherapy |
| 206 | Zhuang Yu | | Efficacy Observation of Dingxia Combined with Press Needle in Treating Vomiting in Cervical Cancer Patients After Chemotherapy | Non-highly emetogenic chemotherapy |
| 207 | Zou Chengxiu | | Clinical Efficacy Observation of Buzhong Yiqi Decoction Combined with Acupoint Press Needle and Chemotherapy in Treating Advanced Lung Adenocarcinoma | Non-highly emetogenic chemotherapy |
| 208 | Ding Zhenyang | | The Impact of Different Auricular Therapies on Digestive Symptoms and Quality of Life in Platinum-Based Chemotherapy Patients | control group does not match |
| 209 | He Yanyan | | The Impact of Different Timing of Acupuncture Intervention on Nausea and Vomiting Induced by Lung Cancer Chemotherapy | Non-highly emetogenic chemotherapy |
| 210 | Wang Yaling | | The Impact of Different Time Windows of Acupuncture Intervention on Chemotherapy-Related Nausea and Vomiting Symptoms: A Self-Crossover Control Study Report | Non-highly emetogenic chemotherapy |
| 211 | Wen Qian | | Clinical Observation of Electroacupuncture at the Ear Vagus Nerve Point in Preventing Nausea and Vomiting in Malignant Tumor Patients After Chemotherapy | Interventions do not meet |
| 212 | Zhu Weijian | | Efficacy Observation of Electroacupuncture at the Ear Vagus Nerve in Preventing Nausea and Vomiting After Chemotherapy | Non-highly emetogenic chemotherapy |
| 213 | Zhu Weijian | | Comparative Efficacy of Electroacupuncture Combined with Auricular Acupressure in Treating Chemotherapy-Induced Vomiting: 54 Cases | Non-highly emetogenic chemotherapy |
| 214 | Xing Xiaojun | | Observation of Electroacupuncture in Treating Nausea and Vomiting in Breast Cancer Patients with Diabetes Undergoing Chemotherapy | Non-highly emetogenic chemotherapy |
| 215 | Chen Mei | | Efficacy Observation of Ear Moxibustion Combined with Wrist-Ankle Acupuncture in Preventing Chemotherapy-Related Nausea and Vomiting in Lung Cancer Patients | control group does not match |
| 216 | Ma Chao | | Efficacy Observation and Nursing of Auricular Acupressure in Treating Vomiting Induced by Postoperative Chemotherapy in Cardia Cancer Patients | Interventions do not meet |
| 217 | Jiang Ping | | Efficacy Observation of Auricular Acupressure Combined with Hegu Acupoint Pressure in Reducing Nausea in Chemotherapy Patients | Non-highly emetogenic chemotherapy |
| 218 | He Honglei | | Clinical Observation of Auricular Acupressure Combined with Acupoint Application in Preventing Chemotherapy-Induced Nausea and Vomiting in Tumor Patients | Non-highly emetogenic chemotherapy |
| 219 | Jiang Lu | | Efficacy Observation of Auricular Acupressure Combined with Acupoint Massage in Preventing Nausea and Vomiting in Breast Cancer Patients After Chemotherapy | Non-highly emetogenic chemotherapy |
| 220 | Hu Yurong | | Clinical Study on Auricular Acupuncture in Treating Nausea and Vomiting Induced by Breast Cancer Chemotherapy | Non-highly emetogenic chemotherapy |
| 221 | Ye Ruchao | | The Role of Auricular Acupressure in Relieving Adverse Reactions in Breast Cancer Patients Undergoing Chemotherapy | Interventions do not meet |
| 222 | Feng Yuanyuan | | Efficacy Observation of Auricular Acupressure Massage in Preventing Cisplatin-Induced Vomiting in Tumor Patients | outcomes don't match |
| 223 | Yang Wenyu | | Clinical Observation of Press Needle in Treating Nausea and Vomiting in Colorectal Cancer Patients After Postoperative Chemotherapy | Interventions do not meet |
| 224 | Wang Jucui | | Efficacy Observation of Acupuncture, Diet Therapy, and Tegafur in Treating Advanced Pancreatic Cancer Patients | Non-highly emetogenic chemotherapy |
| 225 | Huang Chao | | Efficacy Observation of Auricular Acupressure Combined with Acupoint Application in Preventing Nausea and Vomiting Induced by Chemotherapy in Digestive Tract Tumor Patients | Non-highly emetogenic chemotherapy |
| 226 | Zhou Zhi Li | | Efficacy Analysis of Auricular Acupressure in Preventing Nausea and Vomiting Induced by Chemotherapy in Gynecological Tumor Patients | Non-highly emetogenic chemotherapy |
| 227 | He Xiaodan | | Efficacy Observation of Auricular Acupressure Combined with Umbilical Therapy in Preventing Chemotherapy-Induced Nausea and Vomiting | Non-highly emetogenic chemotherapy |
| 228 | Hou Wei | | Observation of Electroacupuncture in Treating Nausea and Vomiting in Breast Cancer Patients with Diabetes Undergoing Chemotherapy | Non-highly emetogenic chemotherapy |
| 229 | Chen Yilin | | The Impact of Acupuncture, Ginger Moxibustion, and Warm Needling Compared to Granisetron on Gastrointestinal Reactions in Cisplatin-Based Chemotherapy Patients at Different Stages | outcomes don't match |
| 230 | Qin Junyun | | Abdominal Acupuncture Combined with Ondansetron Injection in Treating Vomiting in Malignant Bone Tumor Patients After Chemotherapy: | Non-highly emetogenic chemotherapy |
| 231 | Ren Wenjun | | Clinical Efficacy of Acupuncture Based on the “Xingpi Tiaoshen Method” in Treating Chemotherapy-Induced Nausea and Vomiting | Non-highly emetogenic chemotherapy |
| 232 | Liao Guiya | | A Clinical Study on the Detoxification Effect of the Xuanji Needling Technique in Platinum-Containing Chemotherapy Regimens for Malignant Tumors | Non-highly emetogenic chemotherapy |
| 233 | Cai Hu | | Clinical Observation of Acupoint Pressing Needling Combined with Western Medicine in the Prevention and Treatment of Chemotherapy-Induced Nausea and Vomiting in Small Cell Lung Cancer with the EP Regimen | duplicate reserch |
| 234 | Li Yan | | Application of Acupoint Patching Combined with Embedded Needling Intervention Together with Oxaliplatin in Alleviating Nausea and Vomiting Symptoms in Breast Cancer Patients | Non-highly emetogenic chemotherapy |
| 235 | Zhang Zhengyu | | A Clinical Study on Abdominal Needling Combined with Umbilical Patching for Treating Acute Gastrointestinal Adverse Reactions After Chemotherapy in Breast Cancer | Non-highly emetogenic chemotherapy |
| 236 | Li Xiaoxiang | | Observation of the Alleviating Effect of Ear Seed Embedding Combined with Wu Zhu Yu Patching on the Yongquan Acupoint for Nausea and Vomiting in Post-Radical Surgery Chemotherapy Patients with Colorectal Cancer | Non-highly emetogenic chemotherapy |
| 237 | Zhang Hanwen | | Effect of Buccal (Cheek) Needling During the Peri-Chemotherapy Period in Preventing Oxaliplatin-Induced Neurotoxicity and Gastrointestinal Reactions in Colorectal Cancer | Non-highly emetogenic chemotherapy |
| 238 | Qi Jing | | Clinical Observation on the Efficacy of Acupuncture in Preventing and Treating Chemotherapy-Induced Gastrointestinal Reactions | Non-highly emetogenic chemotherapy |
| 239 | Qiu Xiuqiong | | Clinical Analysis of Acupuncture at Neiguan and Zusanli in Inhibiting Cisplatin-Induced Gastrointestinal Reactions | control group does not match |
| 240 | Xie Wenyu | | The Impact of Combined Acupuncture and Acupoint Patching Nursing on Gastrointestinal Reactions in Patients with Gastrointestinal Tumors Undergoing Chemotherapy | Non-highly emetogenic chemotherapy |
| 241 | Guo Xiaoling | | A Study on the Prevention of Vomiting During Chemotherapy for Gastric Cancer Using Ear Acupressure Patching Combined with Granisetron Hydrochloride | Non-highly emetogenic chemotherapy |
| 242 | Gao Rui | | The Effect of Abdominal Needling Combined with Acupuncture at Zusanli on Post-Chemotherapy Gastrointestinal Side Effects in Lymphoma Patients | Non-highly emetogenic chemotherapy |
| 243 | Gong Junjun | | The Effect of Warm Acupuncture on Acute Stress Reactions and Immune Function During Radiotherapy and Chemotherapy in Patients with Gastrointestinal Malignant Tumors | control group does not match |
| 244 | Feng Yuan | | The Application Effect of Subcutaneous Needle Embedding in Reducing Adverse Reactions During Concurrent Chemoradiotherapy for Cervical Cancer | Interventions do not meet |
| 245 | Zhang Sichong | | The Effect of Ear Seed Embedding Combined with Palonosetron Hydrochloride in Preventing and Treating Nausea and Vomiting in Postoperative Chemotherapy Patients with Breast Cancer | Non-highly emetogenic chemotherapy |
| 246 | Wu Chun | | Clinical Observation of Ear Seed Embedding Combined with Acupuncture at Zusanli for the Prevention and Treatment of Chemotherapy-Induced Nausea and Vomiting in Colorectal Cancer | Non-highly emetogenic chemotherapy |
| 247 | Ye Jianyi | | Observation of the Efficacy of Ear Pressing Needling Combined with Zusanli Injection of Metoclopramide Hydrochloride in Improving Chemotherapy-Induced Nausea and Vomiting in Gynecological Malignant Tumors | Non-highly emetogenic chemotherapy |
| 248 | Duan Heng | | Clinical Observation of the Use of Ear Acupressure Patching in Preventing and Treating Chemotherapy-Induced Vomiting in Lung Cancer Patients | control group does not match |
| 249 | Bi Hongda | | Observation of the Effect of Ear Acupressure Patching on Post-Chemotherapy Vomiting | control group does not match |
| 250 | Ma Xinrong | | Clinical Observation of Pressing Needling at the Hegu and Neiguan Acupoints in the Treatment of Chemotherapy-Induced Nausea and Vomiting After Rectal Cancer Surgery | Non-highly emetogenic chemotherapy |
| 251 | Li Menglin | | A Self-Controlled Study on the Prevention and Treatment of Chemotherapy-Induced Nausea and Vomiting Using Abdominal Needling Combined with Antiemetics | Non-highly emetogenic chemotherapy |
| 252 | Zou Xiumei | | Clinical Observation of Pressing Needling Combined with Ondansetron in the Treatment of Nausea and Vomiting After Chemotherapy for Malignant Tumors | Non-highly emetogenic chemotherapy |
| 253 | Zhou Jin | | Clinical Efficacy of Xian Needling in the Treatment of Chemotherapy-Induced Nausea and Vomiting in Cancer Patients | Non-highly emetogenic chemotherapy |
| 254 | Lu Huanzhang | | The Effect of Pressing Needling and Needle Embedding Combined with a Traditional Chinese Medicine Hot Compress on Gastrointestinal Reactions and Quality of Life During Peri-Chemotherapy in Colorectal Cancer | Non-highly emetogenic chemotherapy |
| 255 | Jiang Lijin | | The Improvement Effect of Pressing Needling and Needle Embedding Treatment on Post-Chemotherapy Nausea and Vomiting Symptoms in Colorectal Cancer Patients | Non-highly emetogenic chemotherapy |
| 256 | Zhao Lan | | Observation of the Effect of Pressing Needling and Needle Embedding Treatment in Improving Post-Chemotherapy Nausea and Vomiting Symptoms in Colorectal Cancer Patients | Non-highly emetogenic chemotherapy |
| 257 | Zhang Shuwen | | Pre-Embedded Pressing Needling at Neiguan and Zusanli for the Treatment of Chemotherapy-Induced Nausea and Vomiting in 60 Rectal Cancer Patients | Non-highly emetogenic chemotherapy |
| 258 | Lu Daozhen | | Clinical Observation of the Efficacy of Acupoint Pressing Needling and Needle Embedding in Treating Chemotherapy-Induced Nausea and Vomiting in Gynecological Malignant Tumors | Non-highly emetogenic chemotherapy |
| 259 | Xuan Jing | | Clinical Observation of Acupoint Patching Combined with Acupuncture in the Treatment of Chemotherapy-Induced Nausea and Vomiting in 30 Cases of NSCLC | Interventions do not meet |
| 260 | Lei Tengteng | | Clinical Efficacy of Acupoint Acupuncture Therapy Combined with Ondansetron in the Treatment of Chemotherapy-Induced Nausea and Vomiting After Breast Cancer Surgery | Non-highly emetogenic chemotherapy |
| 261 | Gan Pingping | | The Effect of Early Ear Acupressure Patching on Nausea and Vomiting in Post-Chemotherapy Patients After Colorectal Cancer Surgery | Interventions do not meet |
| 262 | Xie Teng | | Clinical Observation of Acupuncture Combined with Umbilical Patching and Western Medicine in Preventing Delayed Vomiting Induced by Platinum-Based Chemotherapy | Non-highly emetogenic chemotherapy |
| 263 | Xu Yuping | | Clinical Observation of Acupuncture at Specific Acupoints for the Prevention and Treatment of Chemotherapy-Induced Vomiting in Cancer Patients | duplicate reserch |
| 264 | Hu Gaowu | | Clinical Observation of Acupuncture in the Treatment of Post-Chemotherapy Fatigue, Nausea, and Vomiting in Breast Cancer Patients | control group does not match |
| 265 | Sima Lei | | Clinical Observation of Acupuncture in the Treatment of Cisplatin-Induced Nausea and Vomiting | Interventions do not meet |
| 266 | Wu Baiqiang | | A Clinical Study of Acupuncture at Zusanli for the Prevention and Treatment of Chemotherapy-Induced Vomiting in Lung Cancer | duplicate reserch |
| 267 | Zhang Yu | | Observation of the Effect of Acupuncture in Improving Nausea and Vomiting in Patients with Advanced Cervical Cancer Undergoing Concurrent Chemoradiotherapy | control group does not match |
| 268 | Li Cao | | Clinical Observation of Acupuncture Therapy for Chemotherapy-Induced Vomiting | Not RCT |
| 269 | Cuilian Hu | | Application of Ear Acupressure Seed Pressing Combined with Injection at Zusanli in 30 Cases of Chemotherapy-Related Side Effects in Cancer Patients | Not RCT |
| 270 | Shupin Li | | Clinical Observation of Acupuncture Therapy for Chemotherapy-Induced Vomiting | Not RCT |
| 271 | Yuanfeng Guo | | Clinical Observation of Ear Acupressure Patching Combined with Ondansetron in Preventing Vomiting Induced by Cisplatin in Combined Chemotherapy | outcomes don't match |
| 272 | Huizhen Yang | | Clinical Observation of Acupuncture Therapy Combined with Psychological Suggestion in the Treatment of Chemotherapy-Induced Gastrointestinal Reactions | Not RCT |
| 273 | Zhifen Huang | | Clinical Observation of Acupuncture at Neiguan and Zusanli for the Prevention and Treatment of Gastrointestinal Reactions Induced by Cisplatin and Other Chemotherapeutic Agents | outcomes don't match |
| 274 | Ying Li | | Clinical Observation of the Efficacy of Ear Acupressure Seed Pressing Combined with Antiemetic Therapy in Preventing Chemotherapy-Induced Vomiting | outcomes don't match |
| 275 | Keqin Kuang | | Clinical Observation of Pressing Needling at Neiguan Combined with Antiemetics in the Prevention of Chemotherapy-Induced Vomiting | outcomes don't match |
| 276 | Xiaoying Bao | | Effect Analysis of Ear Acupressure Seed Pressing Combined with Acupoint Patching on Chemotherapy-Induced Nausea and Vomiting in Female Breast Cancer Patients | outcomes don't match |
