# Supplementary material for: Acupuncture for nausea and vomiting induced by highly emetogenic chemotherapy: a systematic review and meta-analysis
Source: Front Neurol. 2026 Jan 5;16:1692411. doi: 10.3389/fneur.2025.1692411 (PMC12812548; doi:10.3389/fneur.2025.1692411)

Supplementary Material 2

## Supplementary Figures

**Supplementary Figure S1 Subgroup analysis of overall completely control rate**


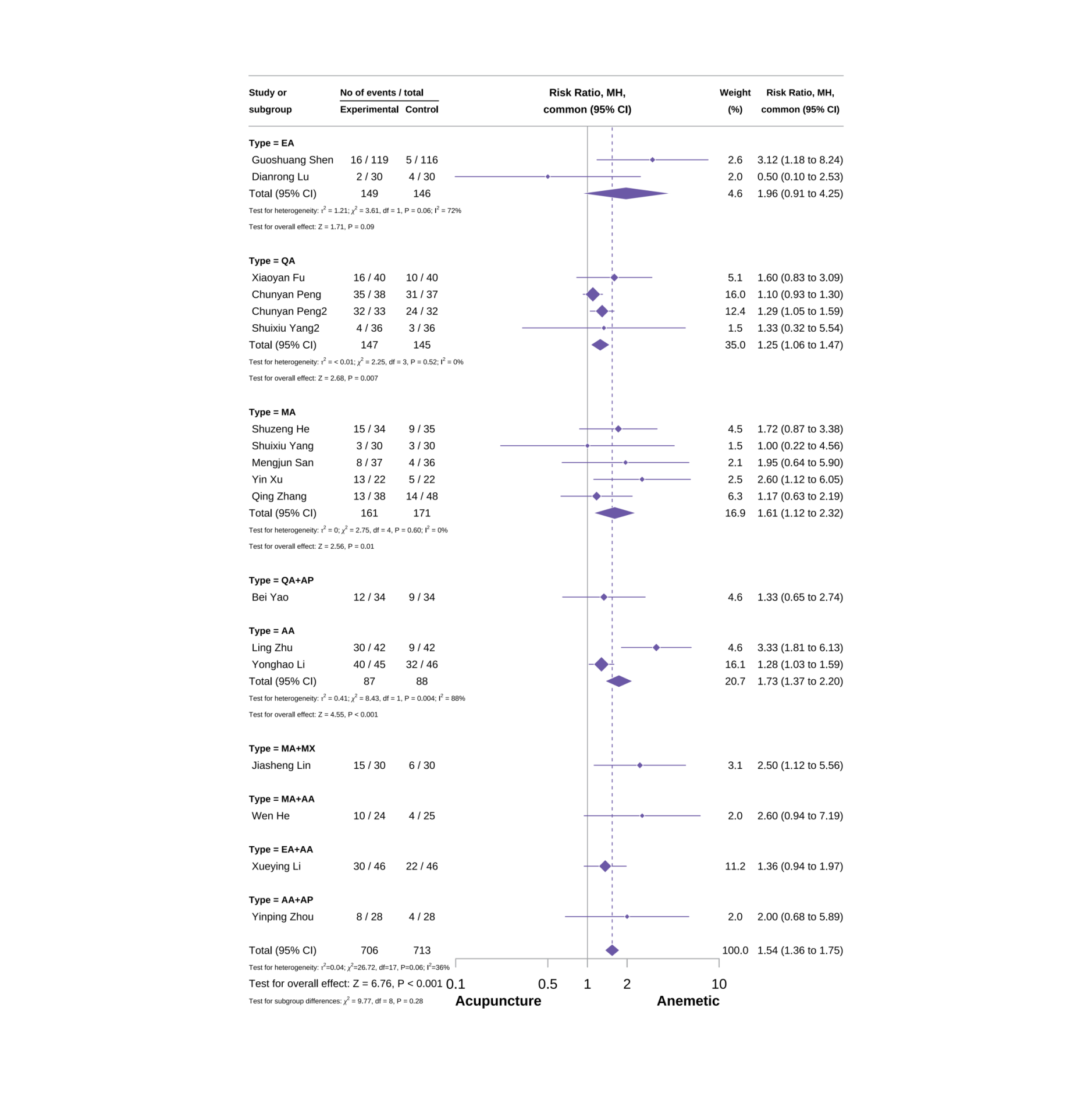


**Supplementary Figure S2 Sensitivity analysis of overall completely control rate**


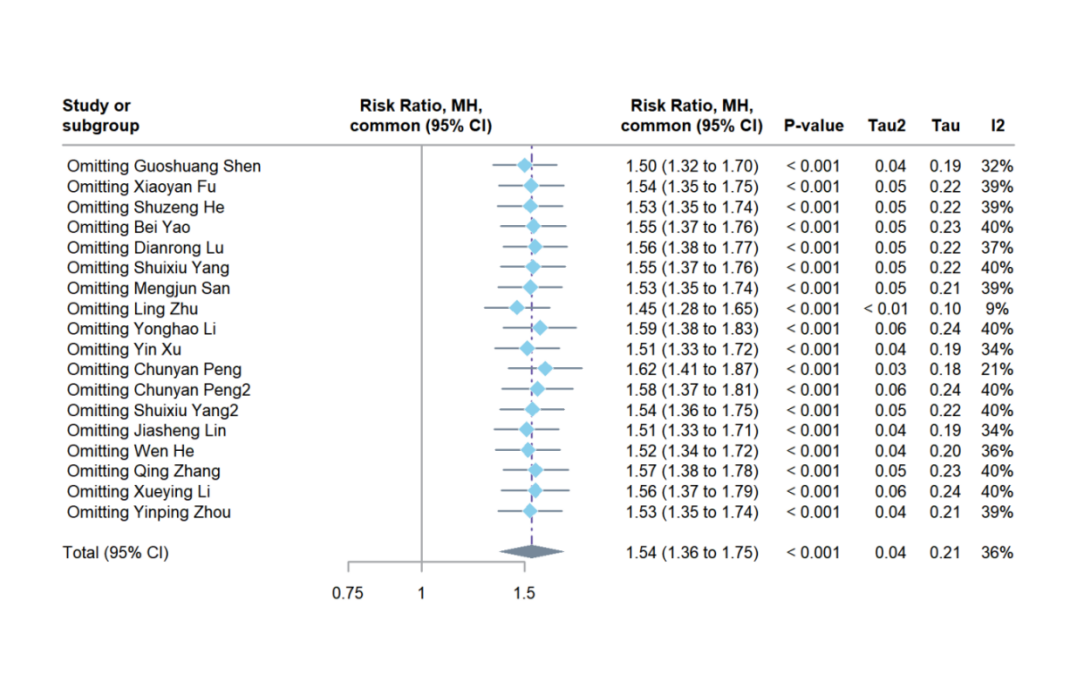


**Supplementary Figure S3 Subgroup analysis of acute completely control rate**


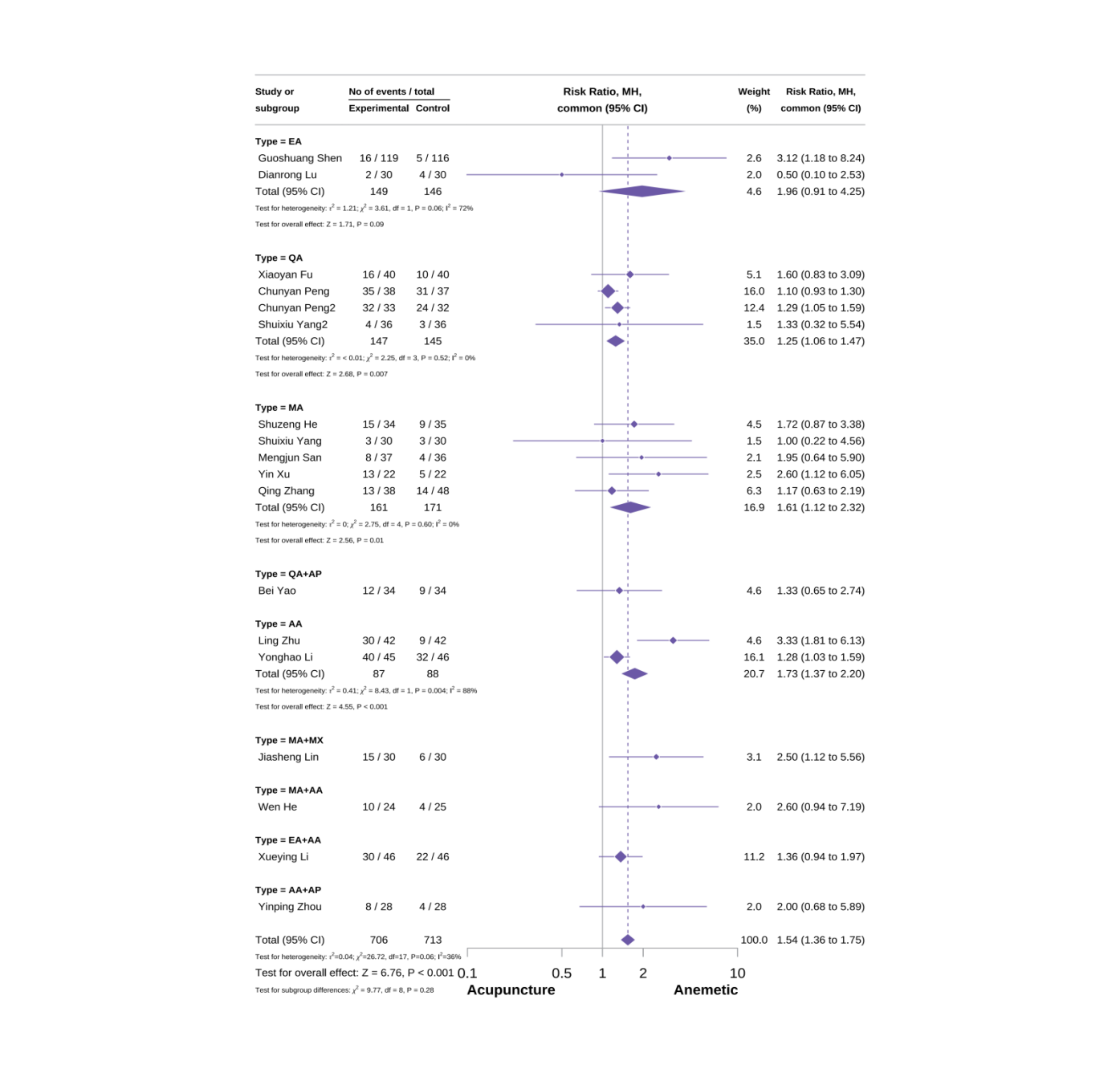


**Figure S4 Sensitive analysis of acute completely control**


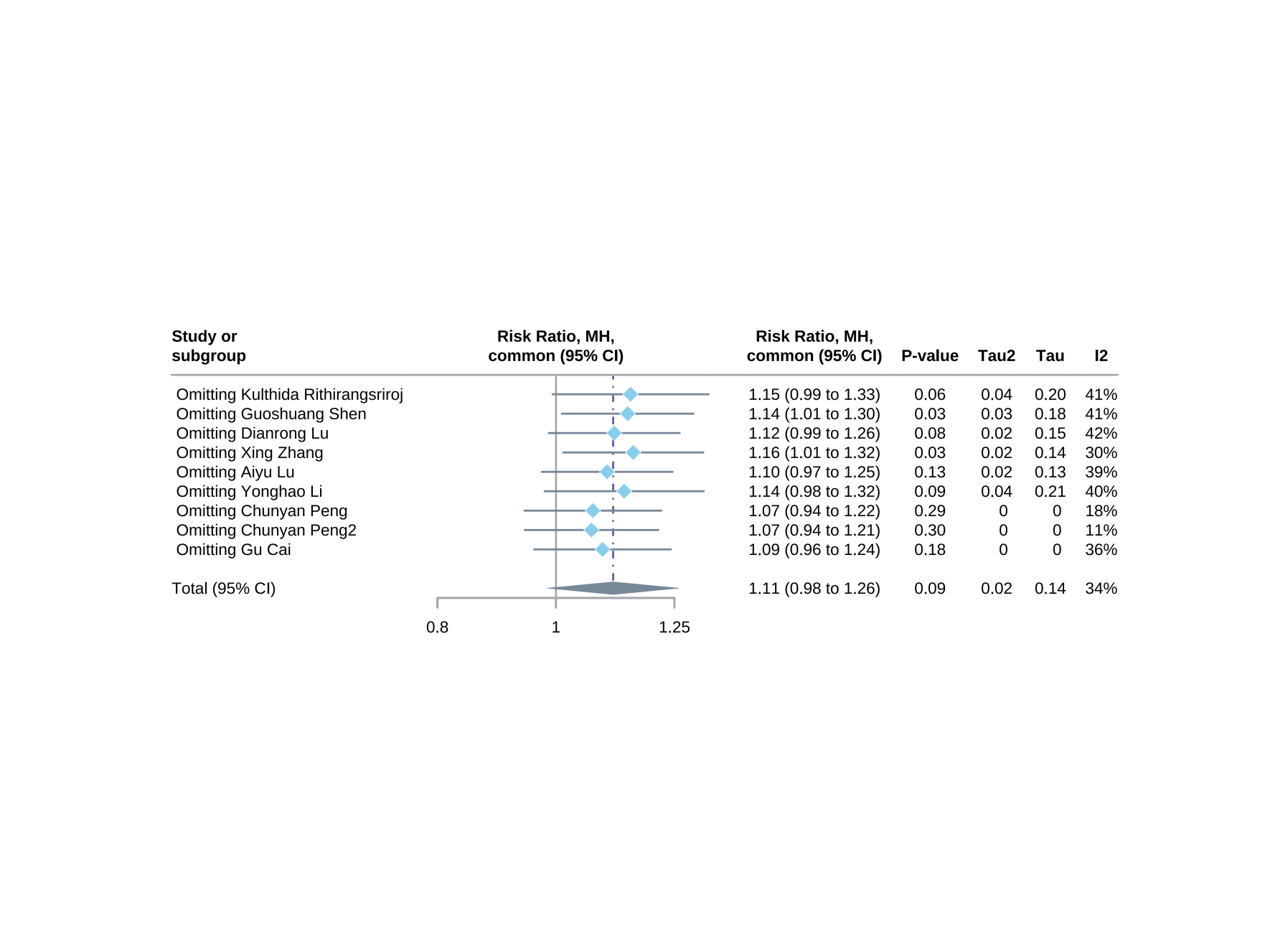


**Figure S5 Subgroup analysis of delay completely control rate**


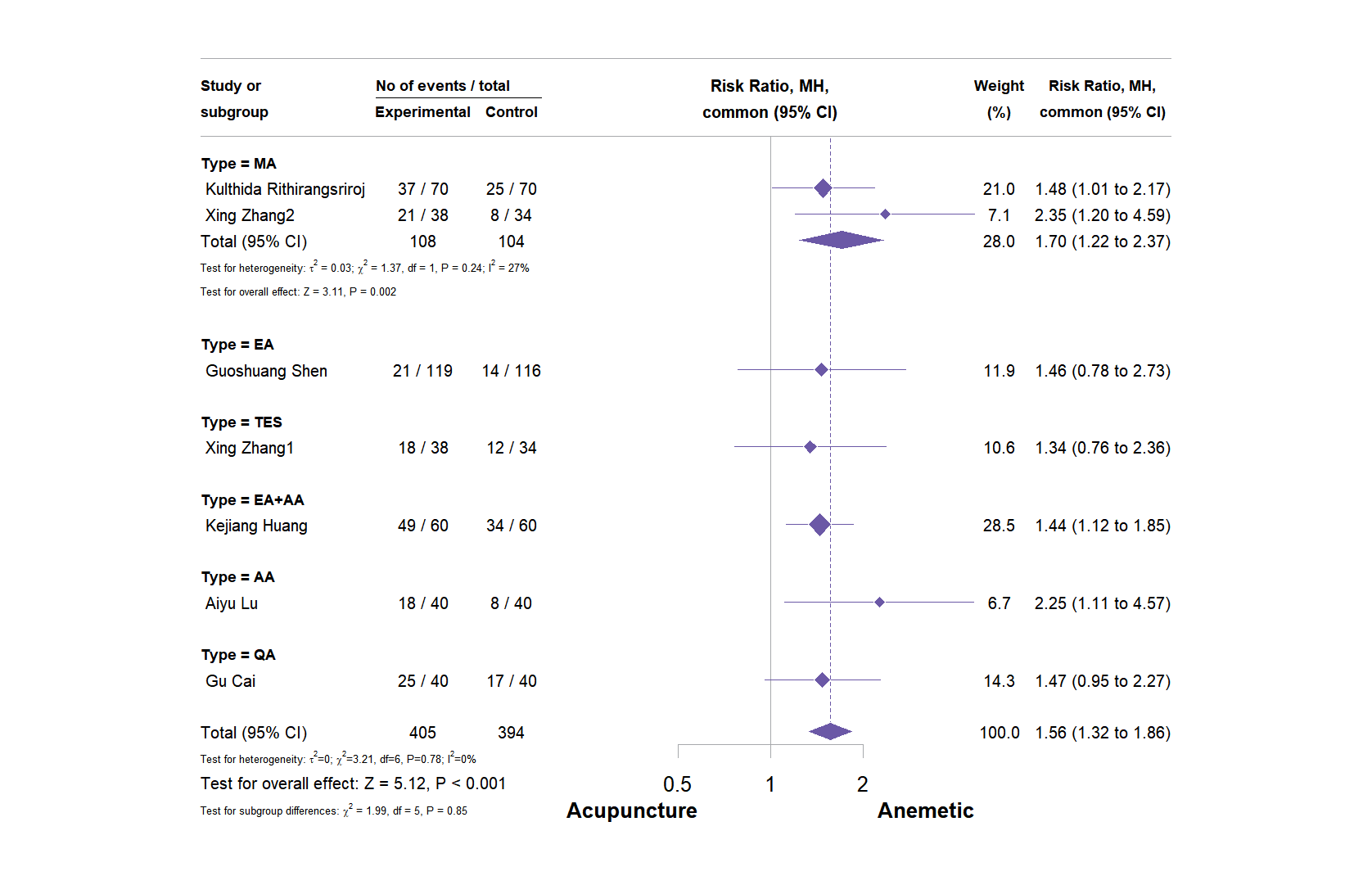


**Figure S6 Sensitive analysis of delay completely control rate**


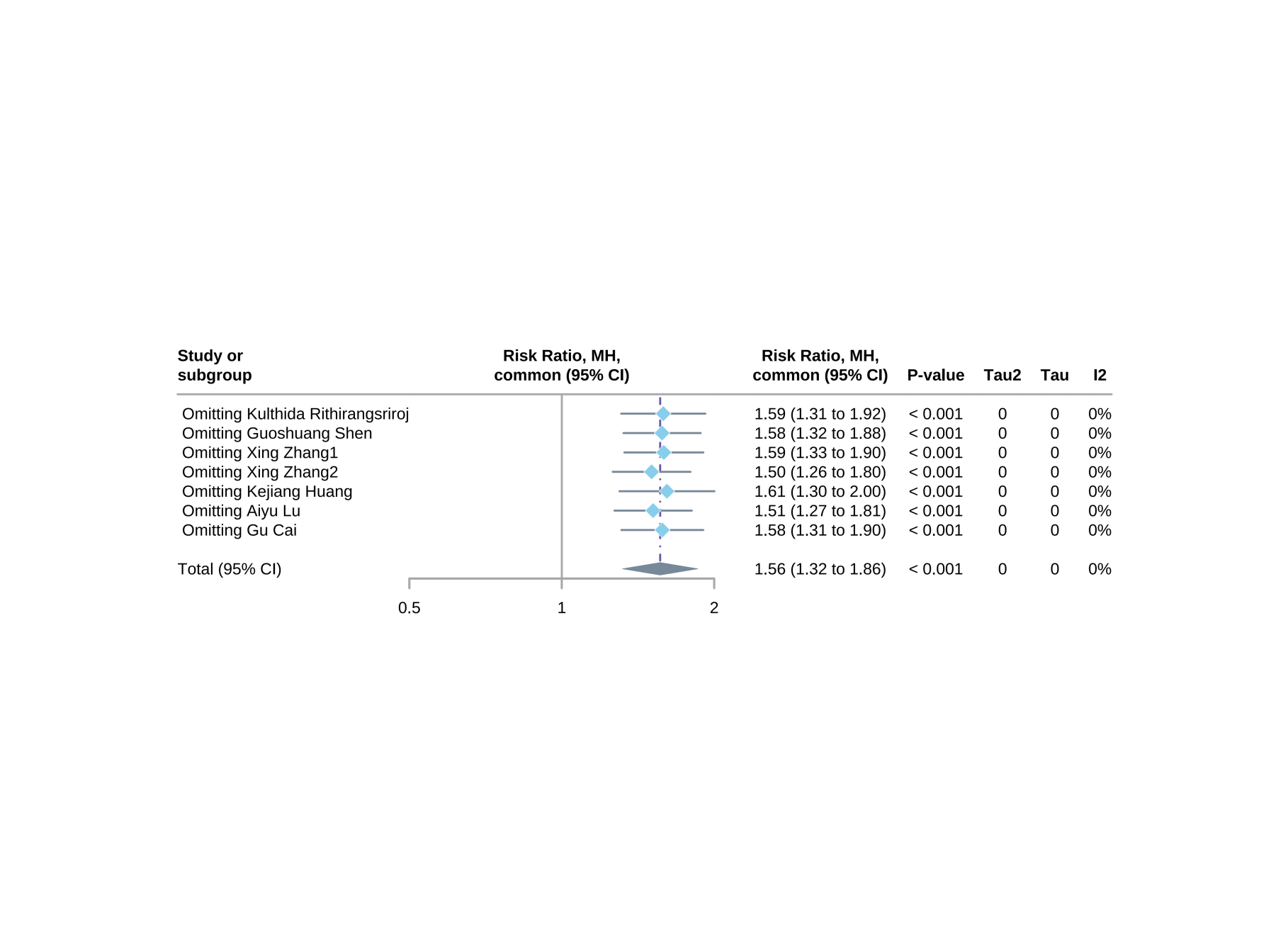

Supplement: Supplementary file 2 [file Table_2.docx]
