# Supplementary material for: Acupuncture for nausea and vomiting induced by highly emetogenic chemotherapy: a systematic review and meta-analysis
Source: Front Neurol. 2026 Jan 5;16:1692411. doi: 10.3389/fneur.2025.1692411 (PMC12812548; doi:10.3389/fneur.2025.1692411)
Supplement: Supplementary file 3 [file Table_3.docx]

Supplementary Material 3

## Supplementary Figures

**Supplementary Figure S1 Subgroup analysis of overall no vomiting events**


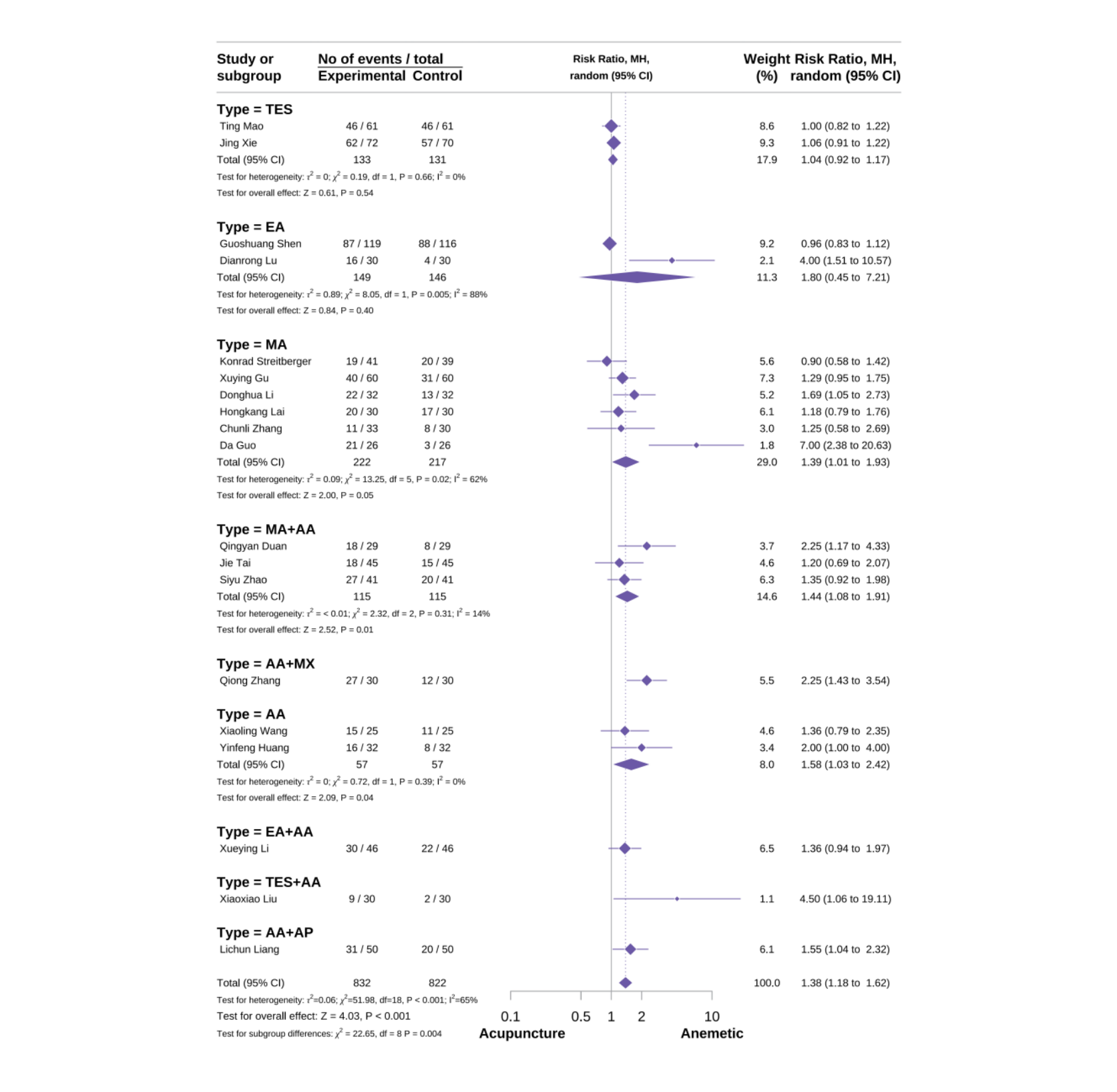


**Supplementary Figure S2 Sensitive analysis and publication bias of overall no vomiting events**


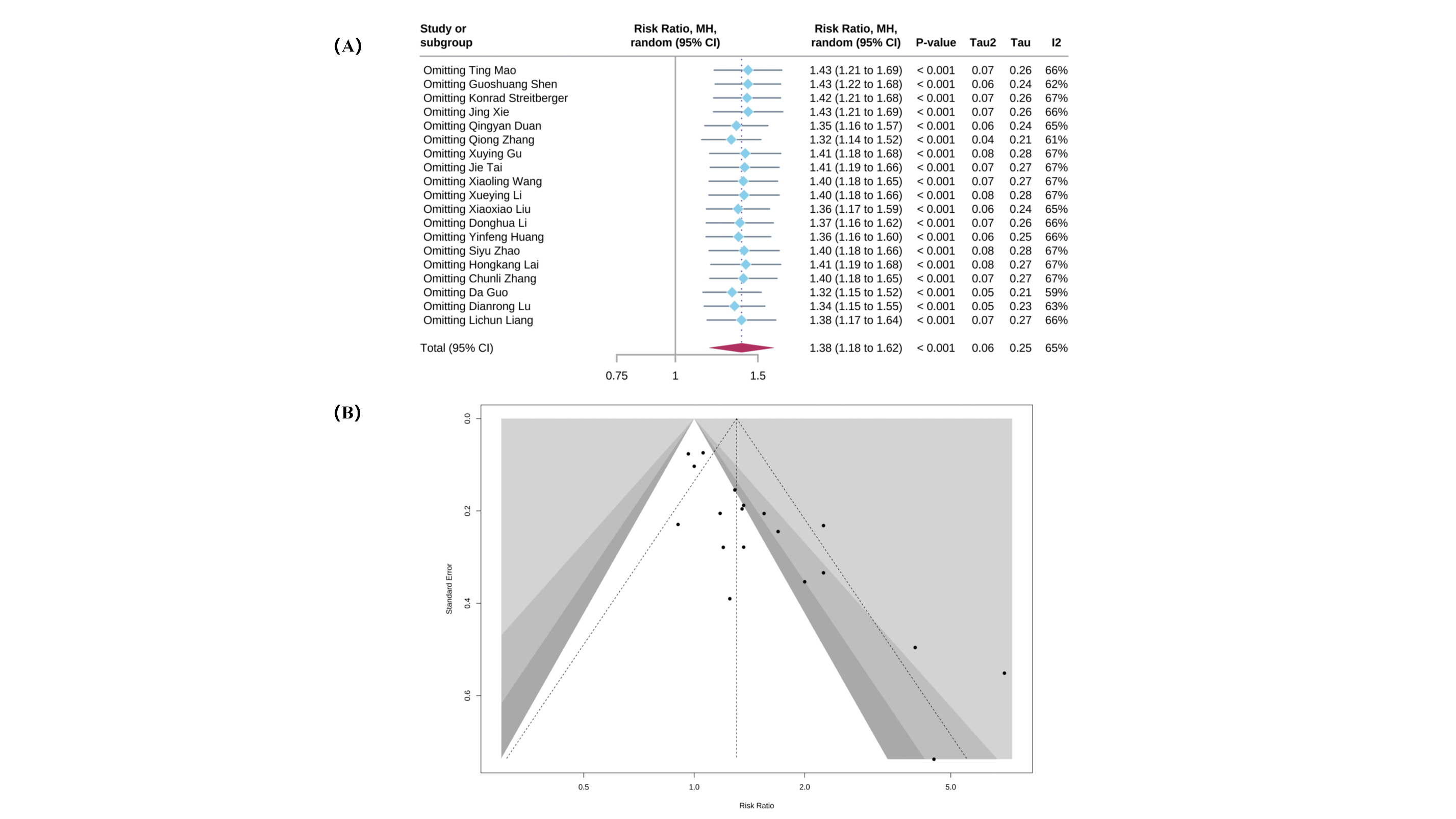


Figure S2 (A) Sensitive analysis of overall nausea severity score (B) publication bias analysis of overall nausea severity score

**Figure S3 Subgroup analysis of overall no significant nausea events**


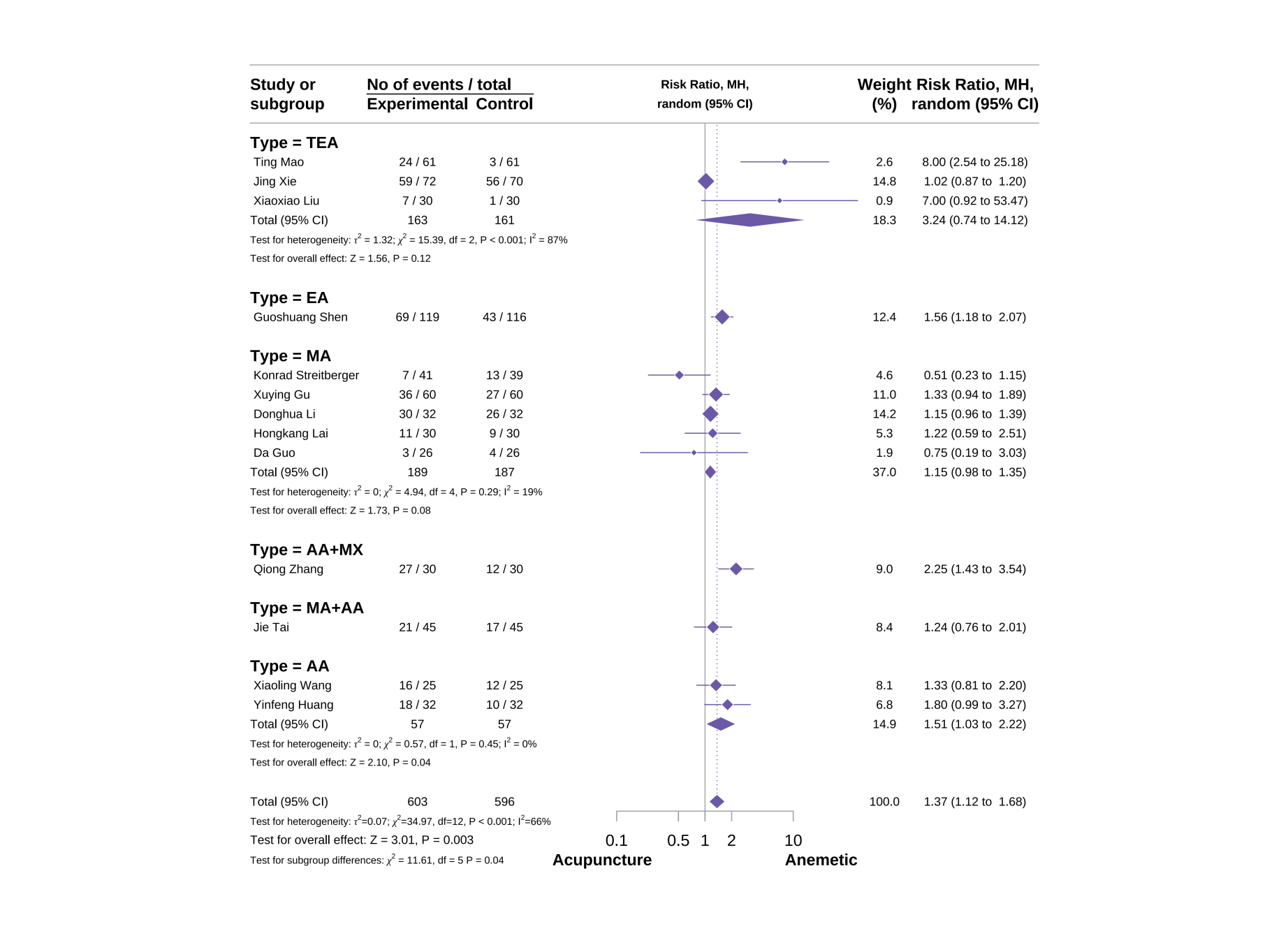


**Figure S4 Sensitive analysis and publication bias of overall no significant nausea events**


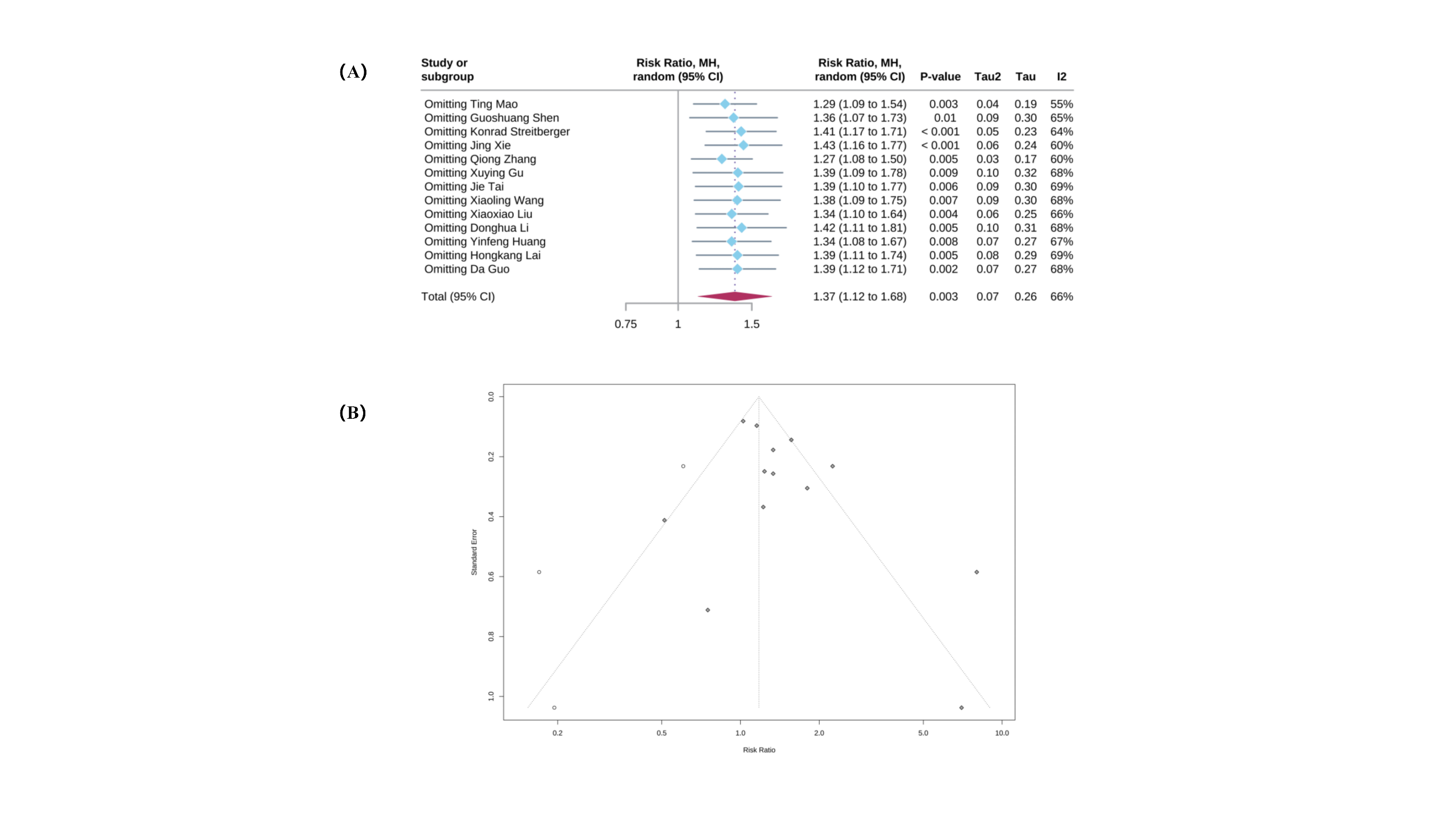


Figure S4 (A) Sensitive analysis of overall no significant nausea events (B) publication bias analysis of overall no significant nausea events

**Figure S5 Subgroup analysis and sensitive analysis of overall vomiting severity score**


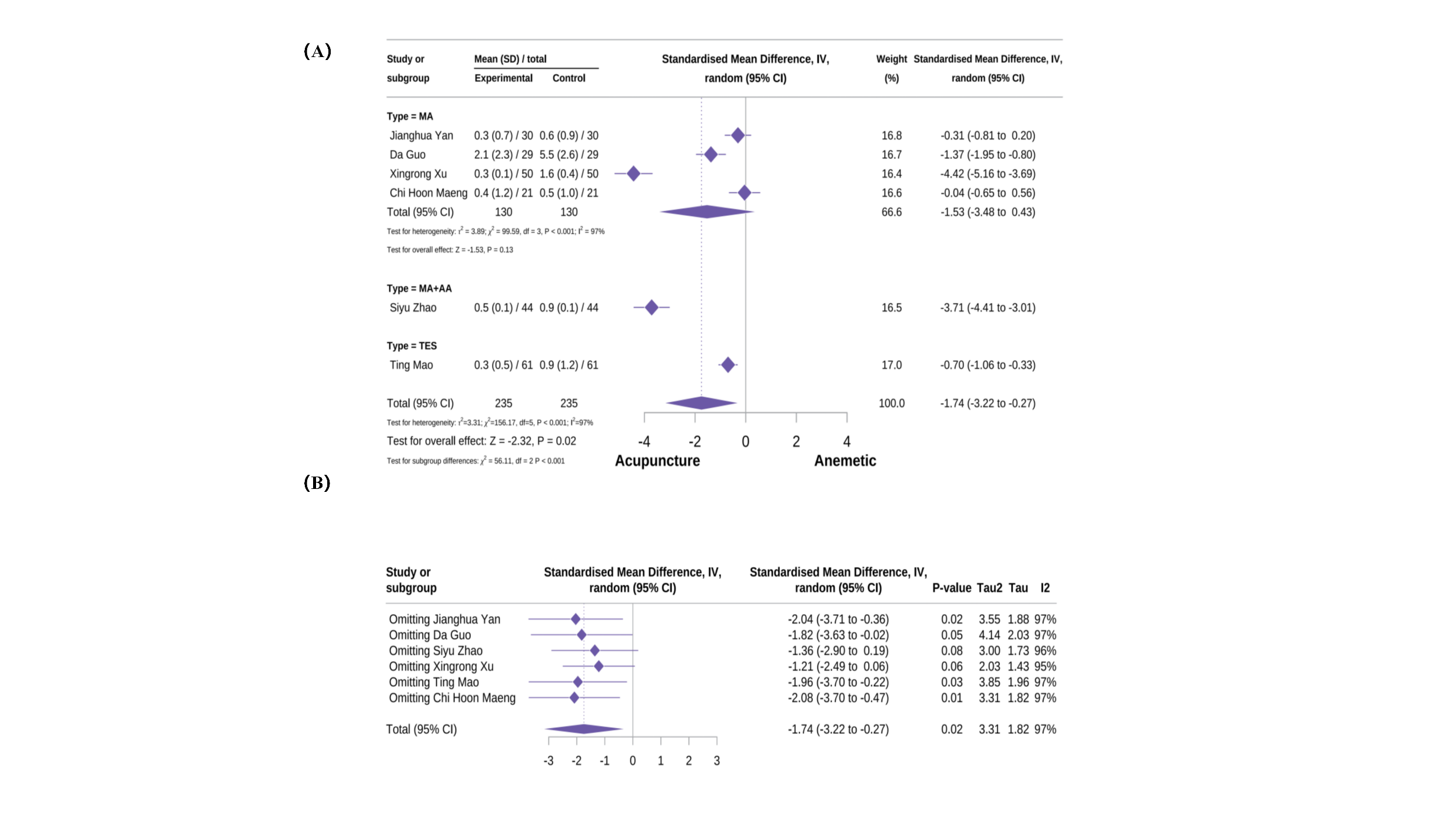


Figure S5 (A) Subgroup analysis of overall overall vomiting severity score (B) Sensitive analysis of overall vomiting severity score

**Figure S6 Subgroup and Sensitive analysis of overall overall nausea severity score**


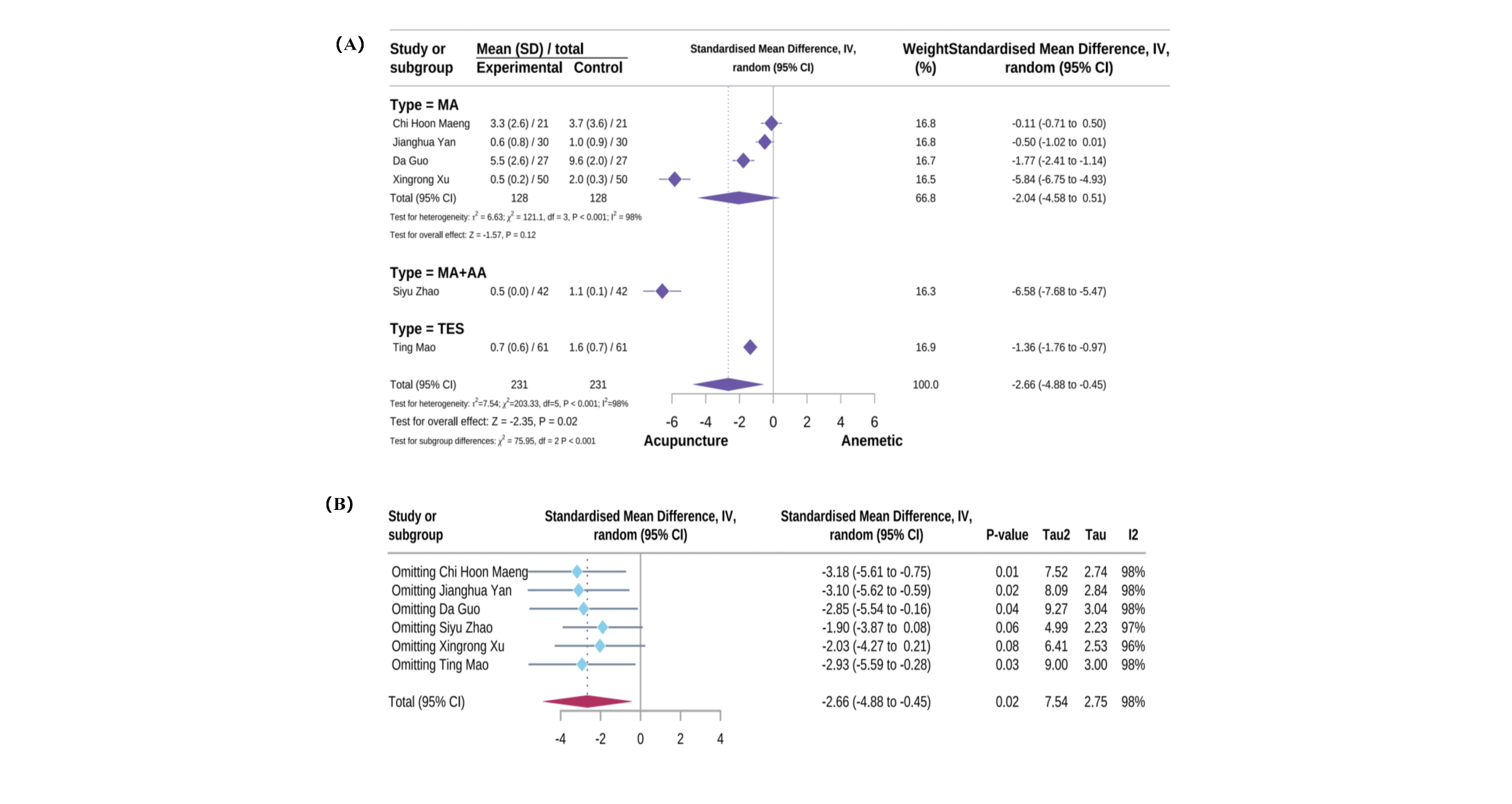


Figure S6 (A) Subgroup analysis of overall nausea severity score (B) Sensitive analysis of overall nausea severity score

**Figure S7 Meta analysis and sensitive analysis of overall nausea frequency score**


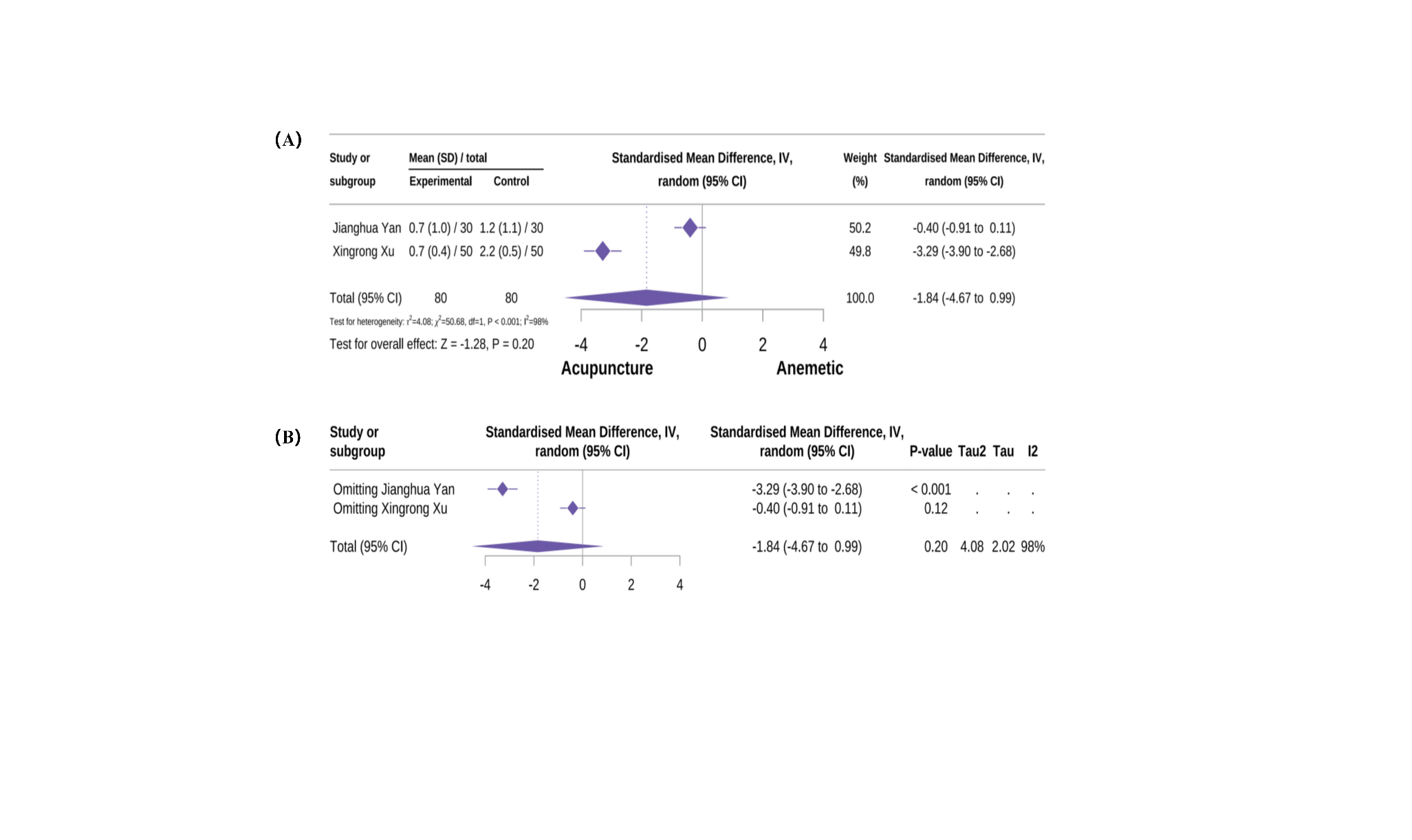


Figure S7 (A) Meta analysis of overall nausea frequency score (B) Sensitive analysis of overall nausea frequency score

**Figure S8 Meta analysis and sensitive analysis of overall nausea duration score**


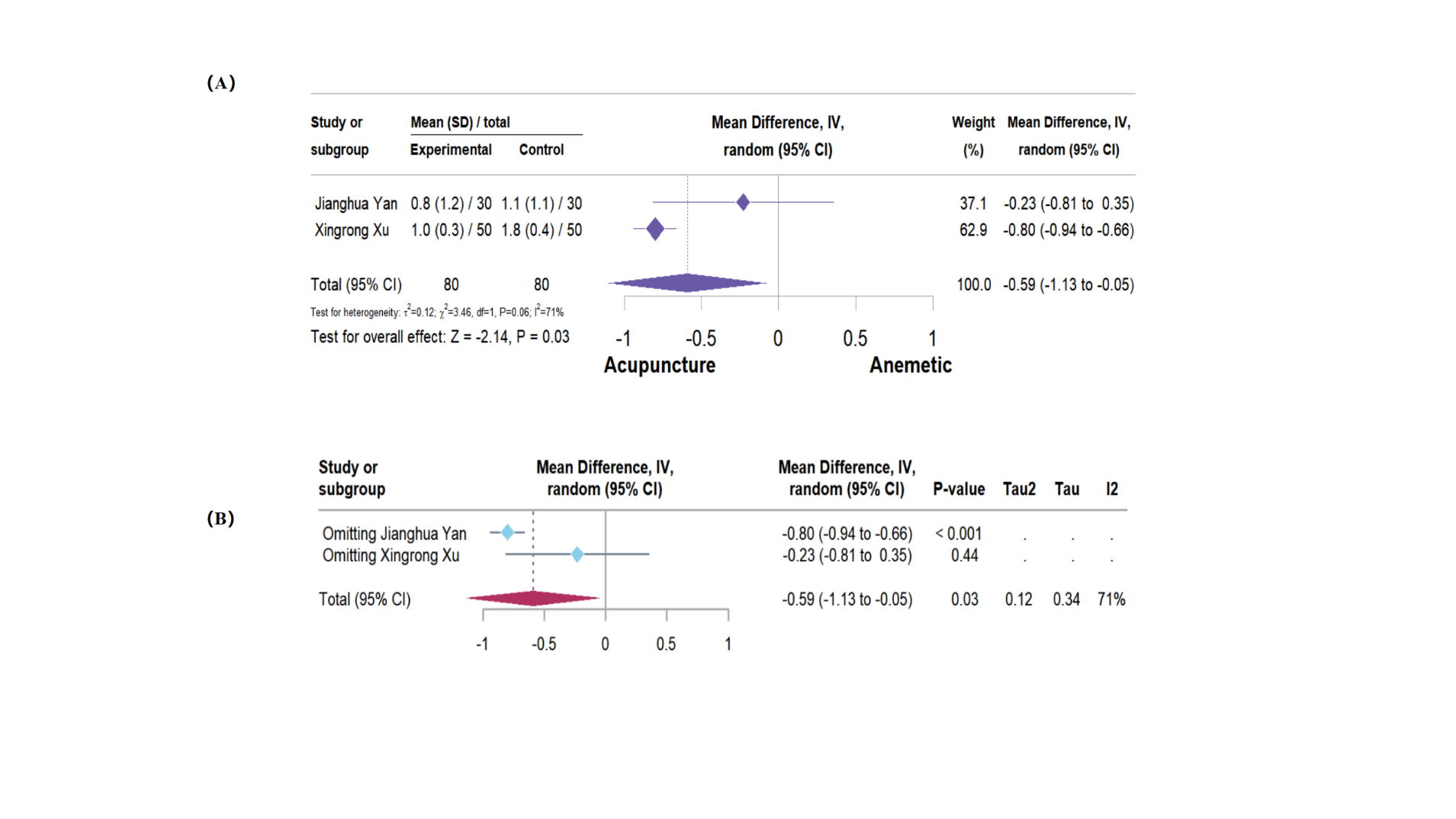


Figure S8 (A) Meta analysis of overall nausea duration score (B) Sensitive analysis of overall nausea duration score

**Figure S9 Subgroup analysis and sensitive analysis of overall CINV score**


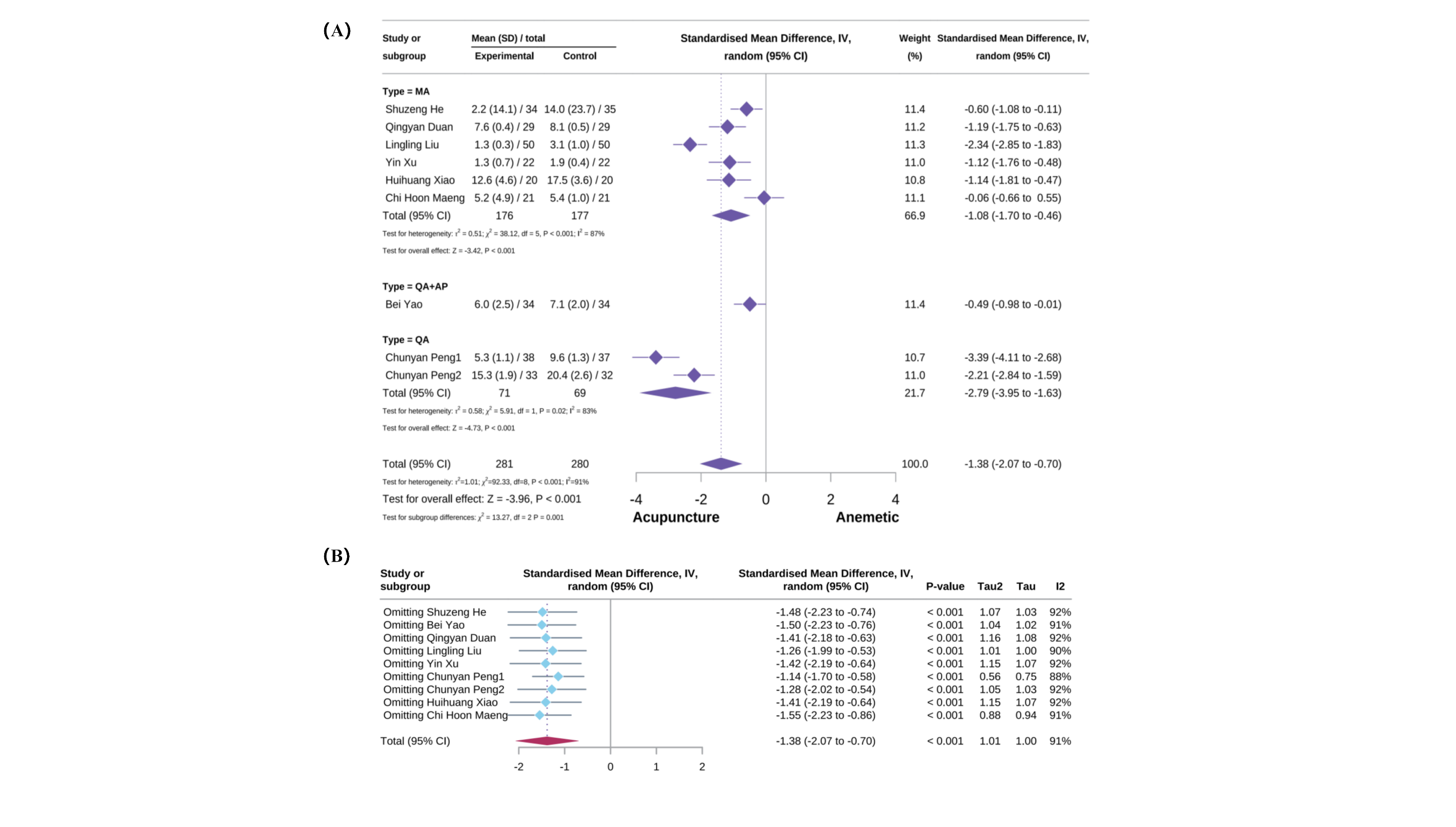


Figure S9 (A) Subgroup analysis of overall CINV score (B) Sensitive analysis of overall CINV score

**Figure S10 Meta analysis, subgroup analysis and sensitive analysis of overall vomiting frequency score**


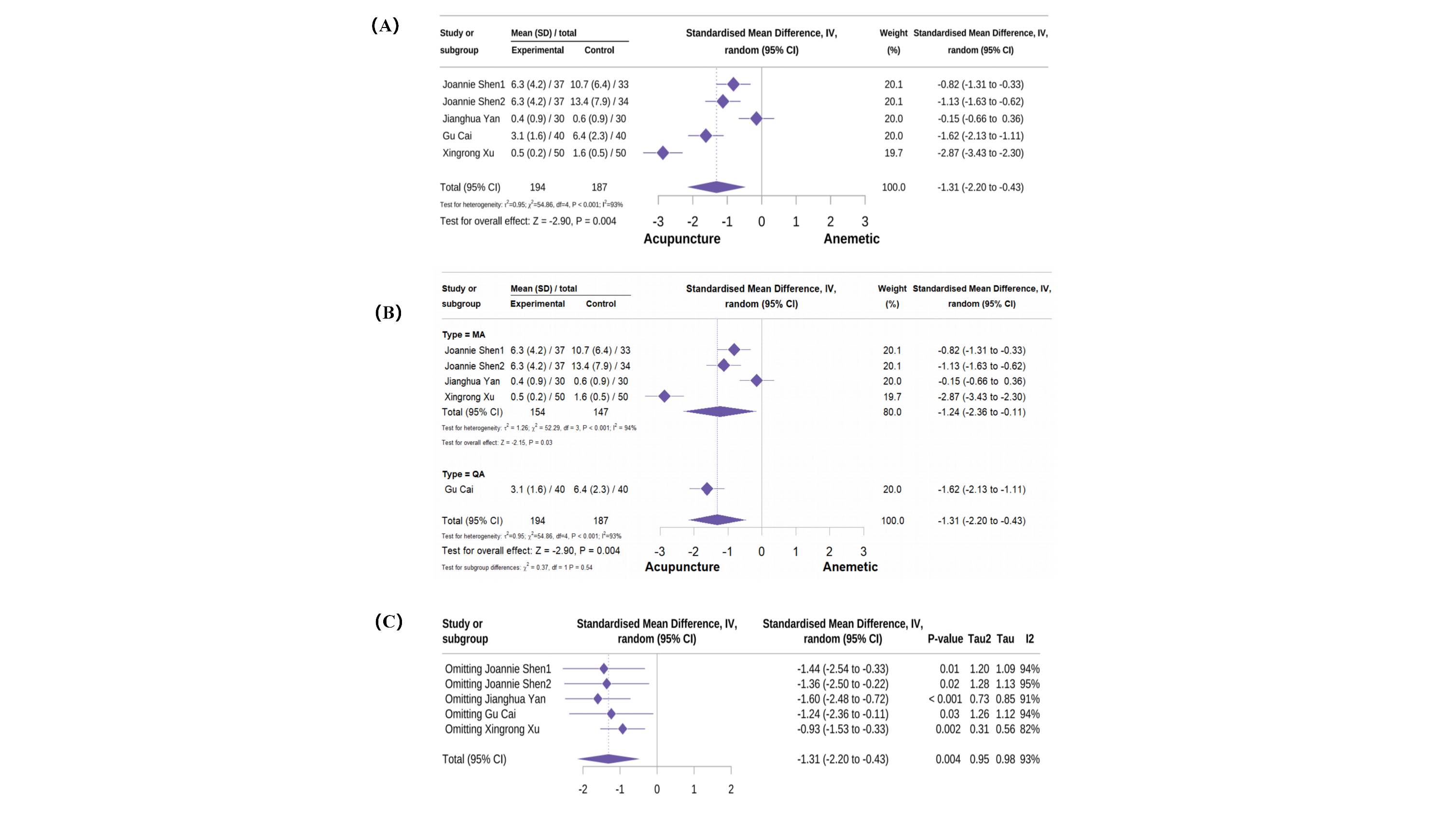


Figure S10 (A) Meta analysis of overall vomiting frequency score (B) Subgroup analysis of overall vomiting frequency score (C) Sensitive analysis of overall vomiting frequency score

**Figure S11 Meta analysis and sensitive analysis of overall overall vomiting volume score**


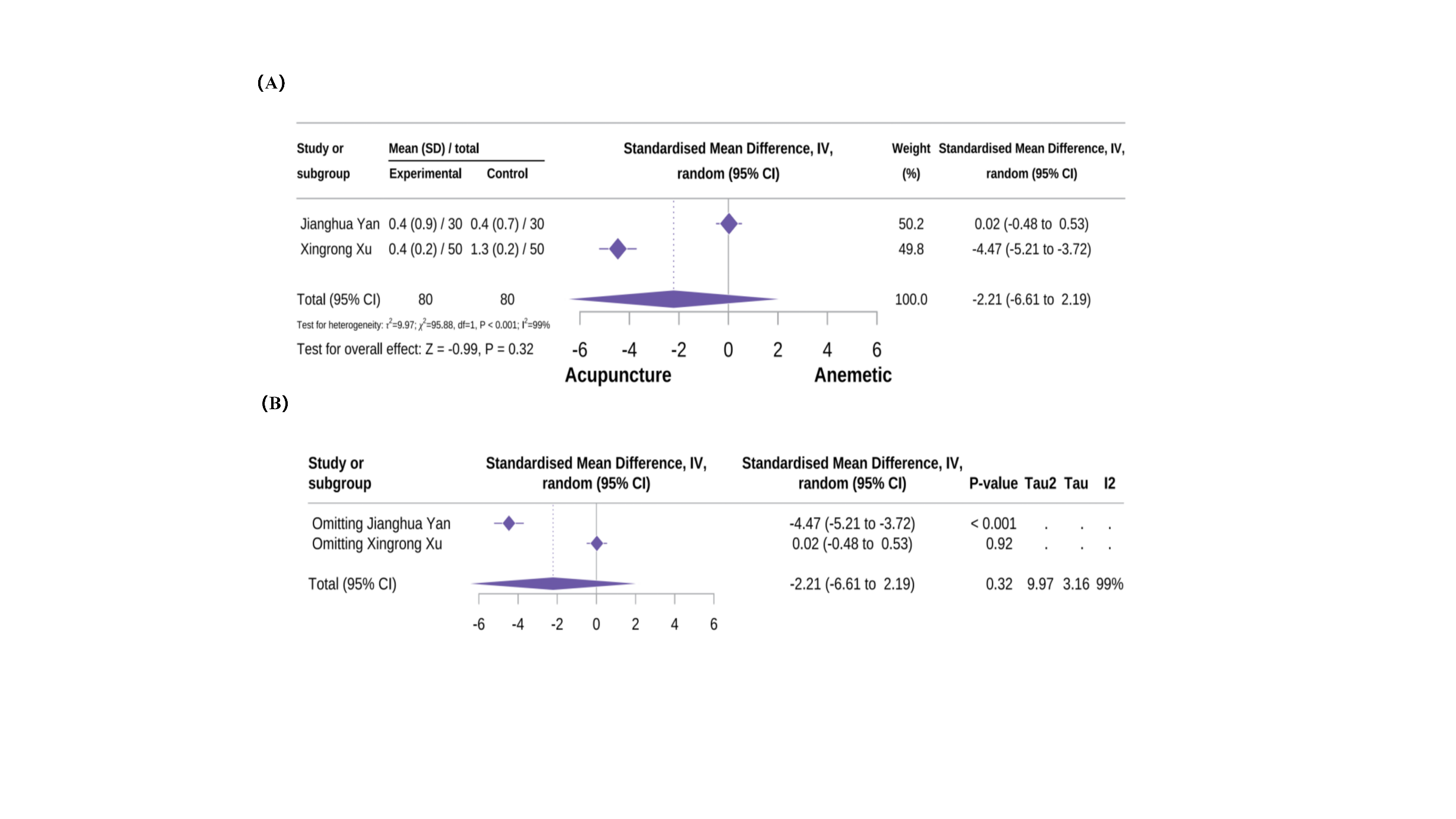


Figure S11 (A) Meta analysis of overall overall vomiting volume score (B) Sensitive analysis of overall overall vomiting volume score
