# Supplementary material for: Acupuncture for nausea and vomiting induced by highly emetogenic chemotherapy: a systematic review and meta-analysis
Source: Front Neurol. 2026 Jan 5;16:1692411. doi: 10.3389/fneur.2025.1692411 (PMC12812548; doi:10.3389/fneur.2025.1692411)
Supplement: Supplementary file 4 [file Table_4.docx]

Supplementary Material 4

## Supplementary Figures

**Supplementary Figure S1 Subgroup analysis of acute no vomiting events**


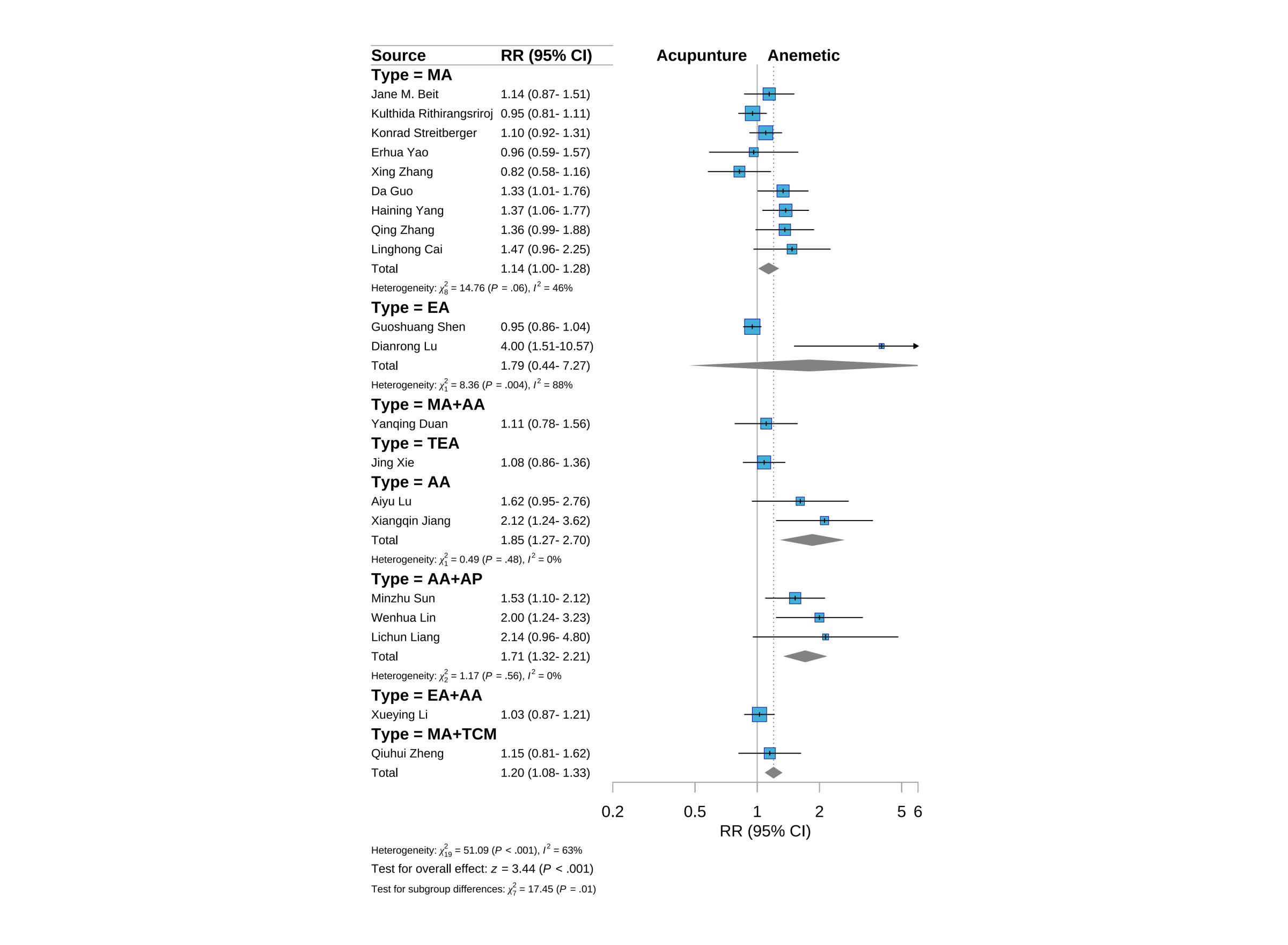


**Supplementary Figure S2 Sensitive analysis and publication bias of acute no vomiting events**


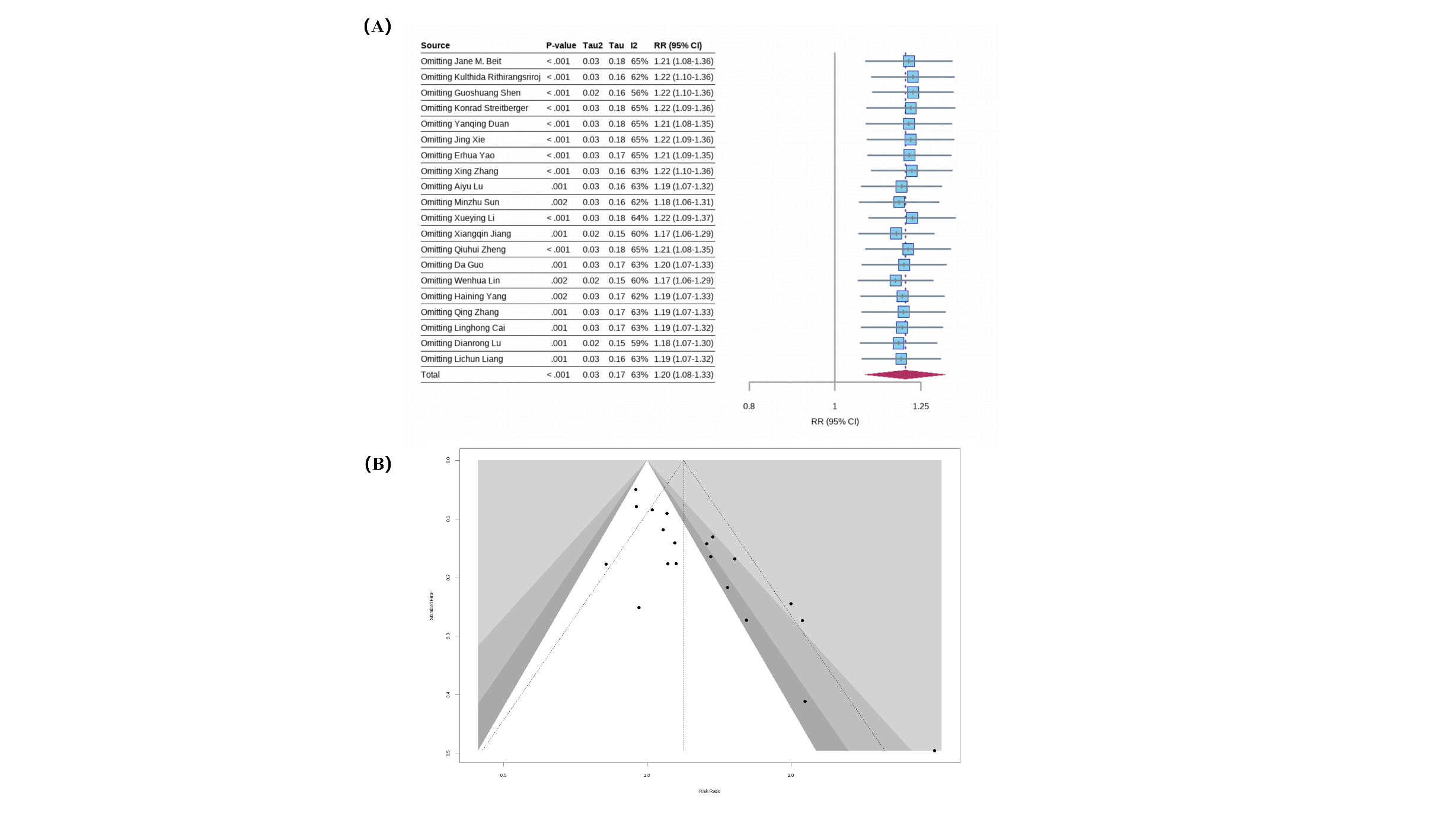


Figure S2 (A) Sensitive analysis of acute no vomiting events (B) publication bias analysis of acute no vomiting events

**Figure S3 Subgroup analysis and sensitive analysis of acute no significant nausea events**


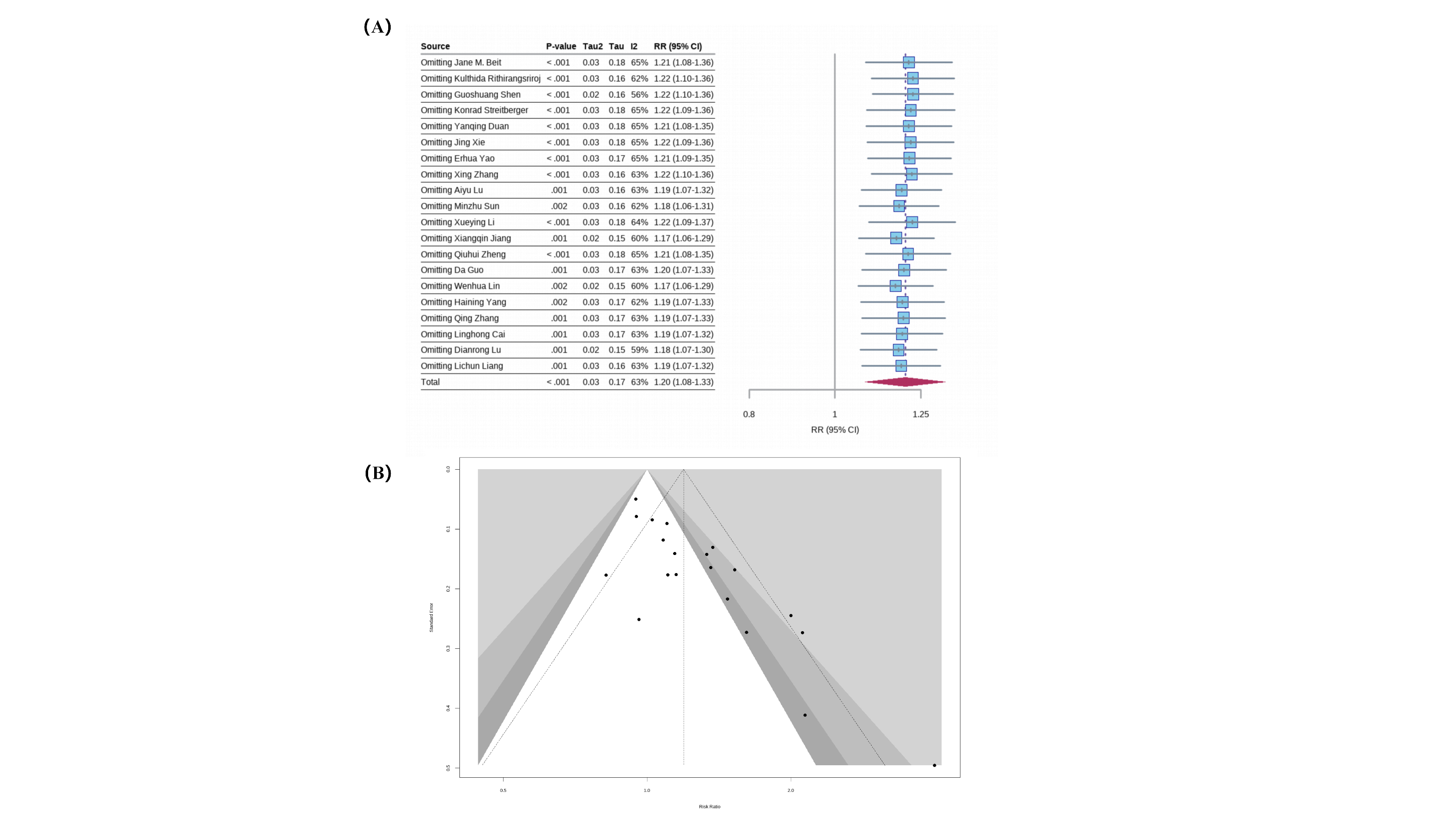


Figure S3 (A) Sensitive analysis of acute no significant nausea events (B) publication bias analysis of acute no significant nausea events

**Figure S4 Subgroup analysis and sensitive analysis of acute vomiting severity score**


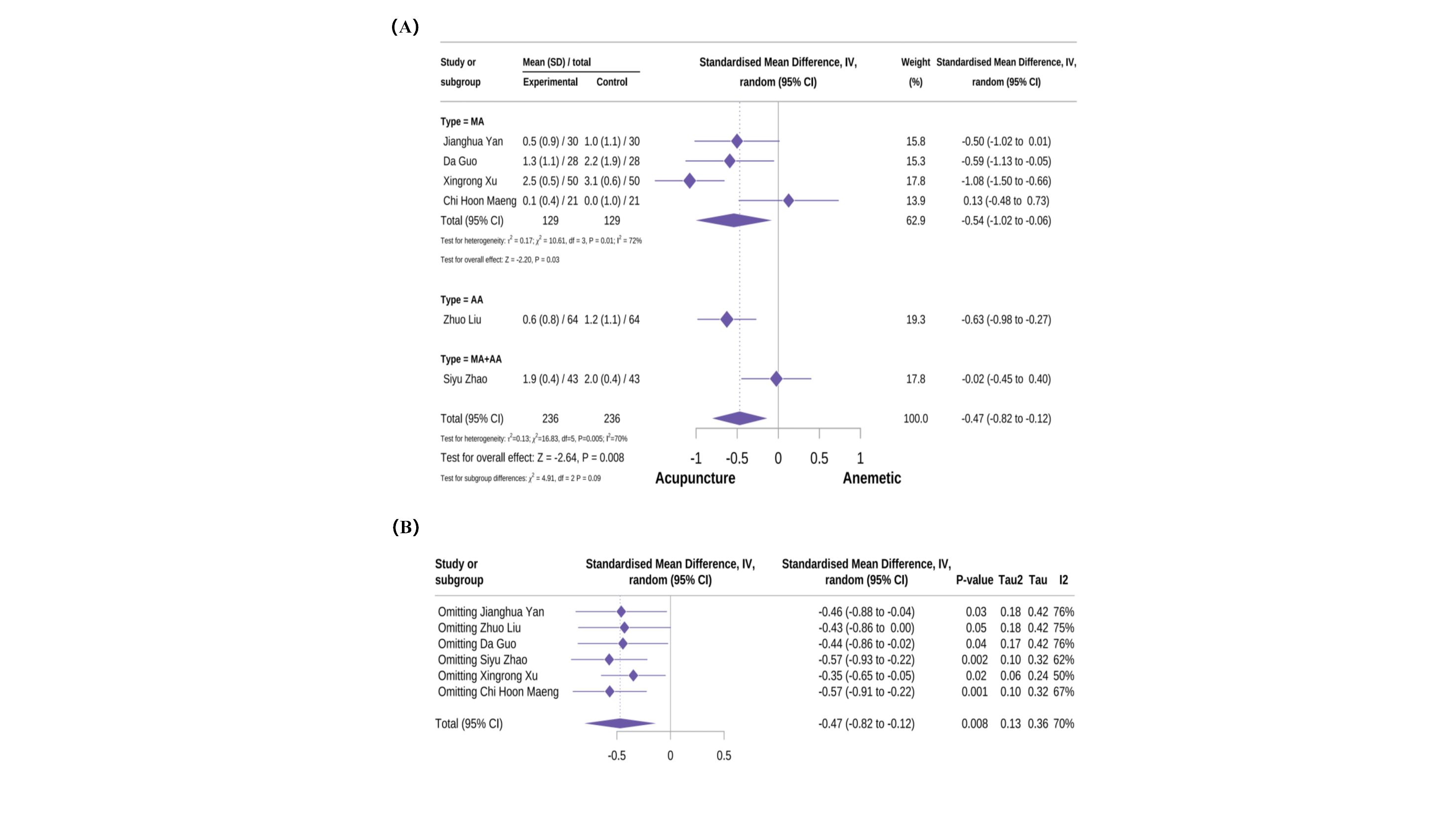


Figure S4 (A) Subgroup analysis of acute vomiting severity score (B) Sensitive analysis of acute vomiting severity score

**Figure S5 Meta analysis, subgroup analysis and sensitive analysis of acute nausea severity score**


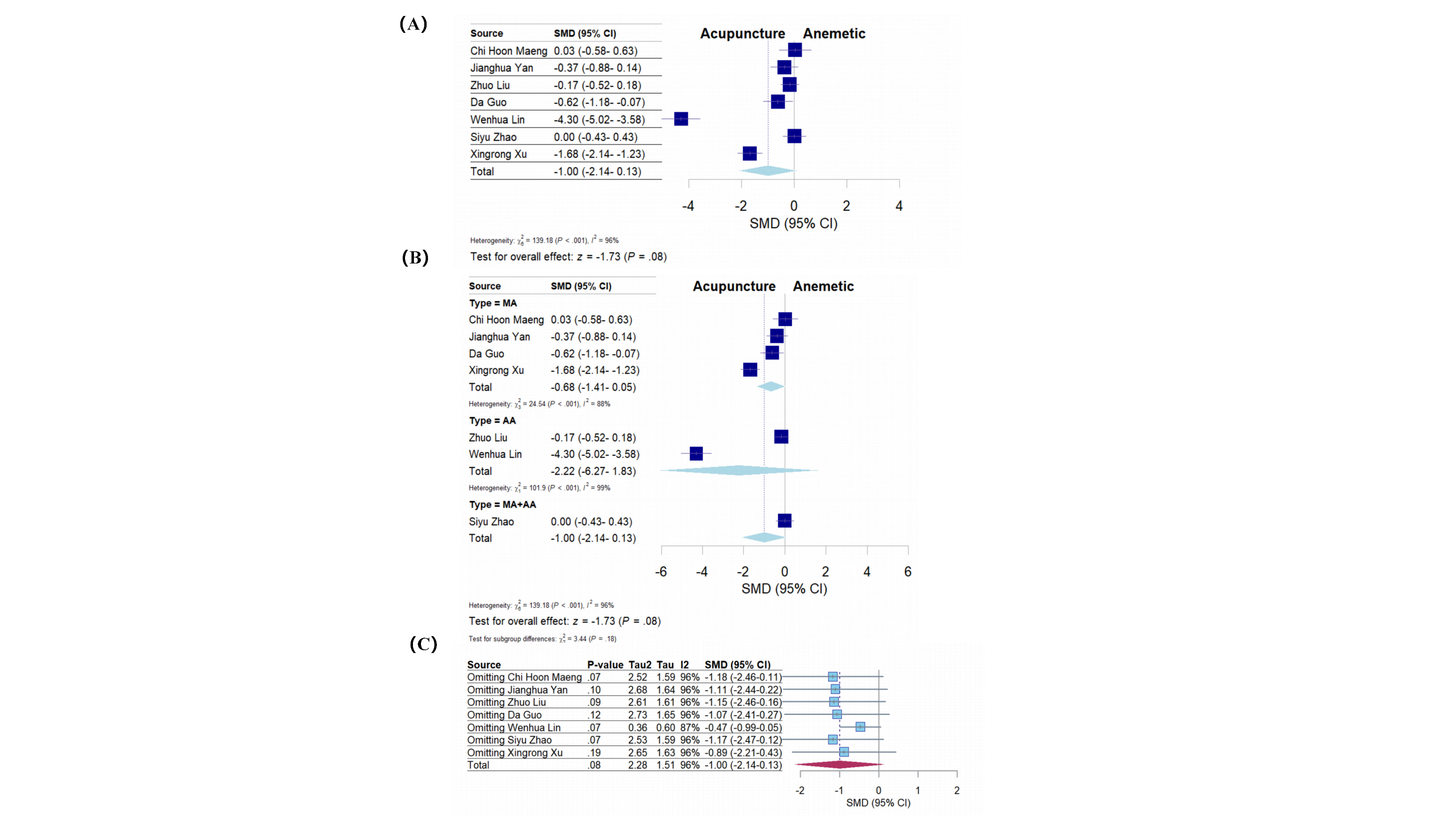


Figure S5 (A) Meta analysis of acute nausea severity score (B) Subgroup analysis of acute nausea severity score (C) Sensitive analysis of acute nausea severity score

**Figure S6 Meta analysis, subgroup analysis and sensitive analysis of acute vomiting frequency score**


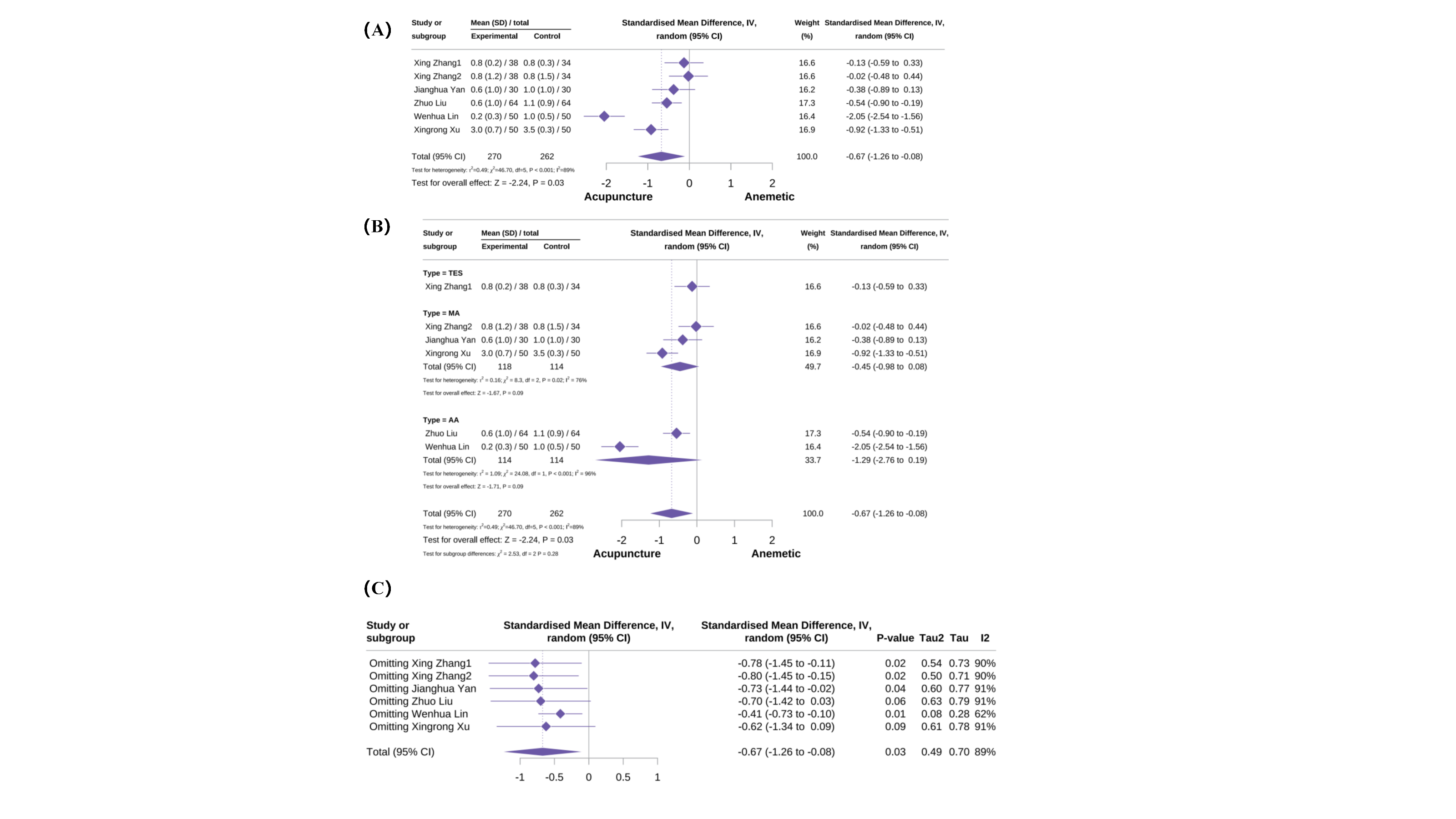


Figure S6 (A) Meta analysis of acute vomiting frequency score (B) Subgroup analysis of acute vomiting frequency score (C) Sensitive analysis of acute vomiting frequency score

**Figure S7 Subgroup analysis and sensitive analysis of acute vomiting volume score**


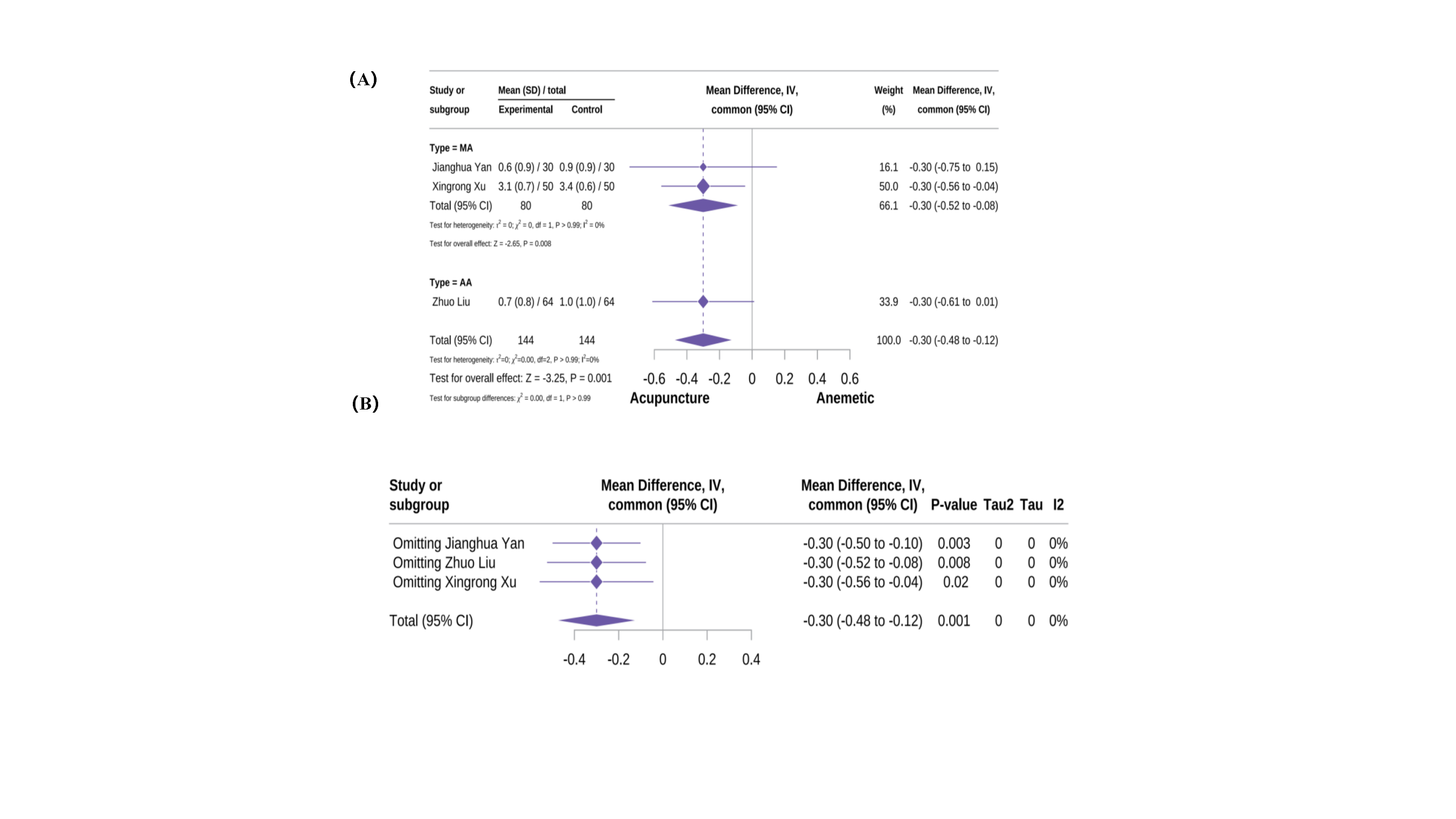


Figure S7 (A) Subgroup analysis of acute vomiting volume score (B) Sensitive analysis of acute vomiting volume score

**Figure S8 Meta analysis, subgroup analysis and sensitive analysis of acute nausea duration score**


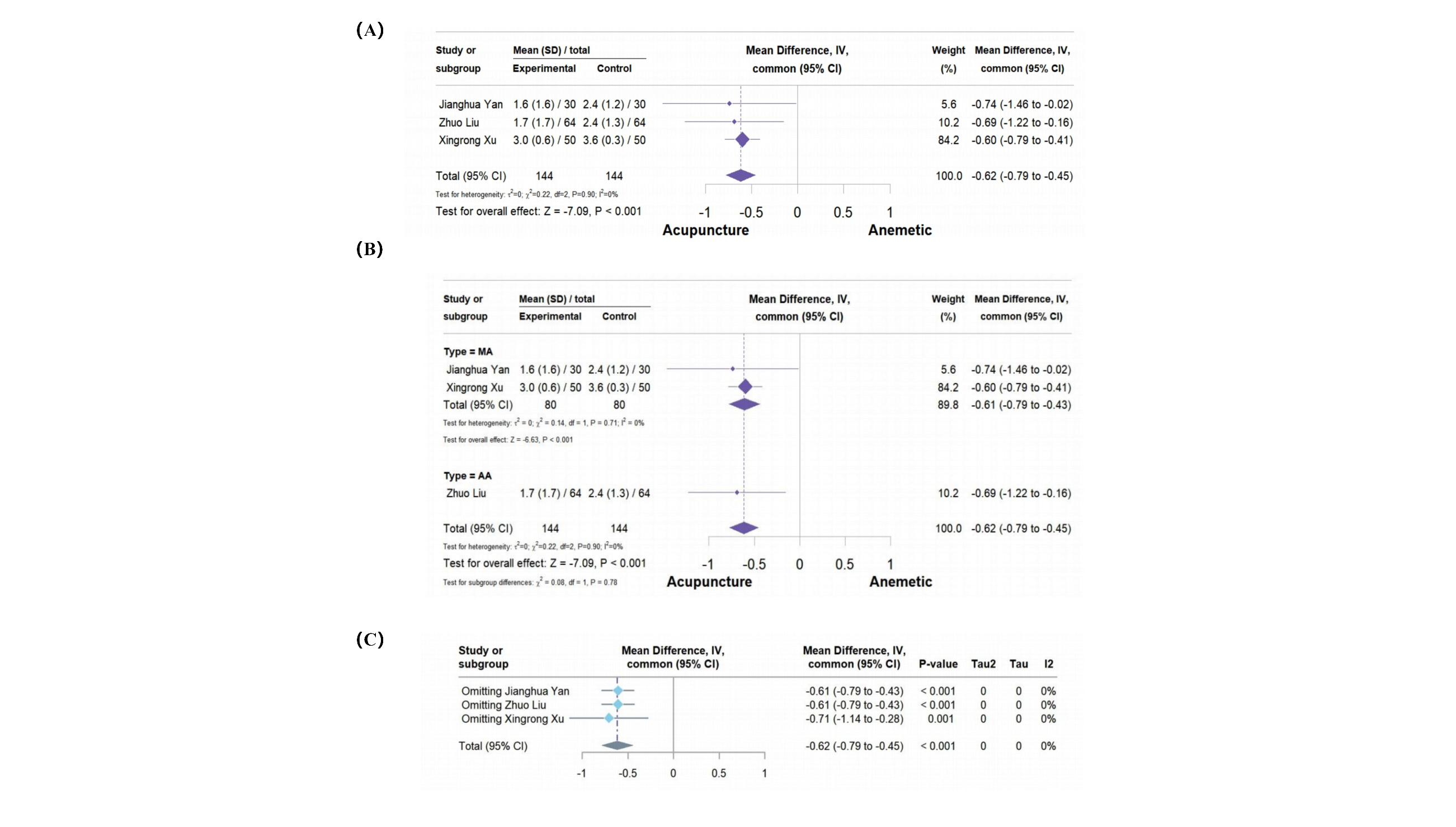


Figure S8 (A) Meta analysis of acute nausea duration score (B) Subgroup analysis of acute nausea duration score (C) Sensitive analysis of acute nausea duration score

**Figure S9 Meta analysis, subgroup analysis and sensitive analysis of acute nausea frequency score**


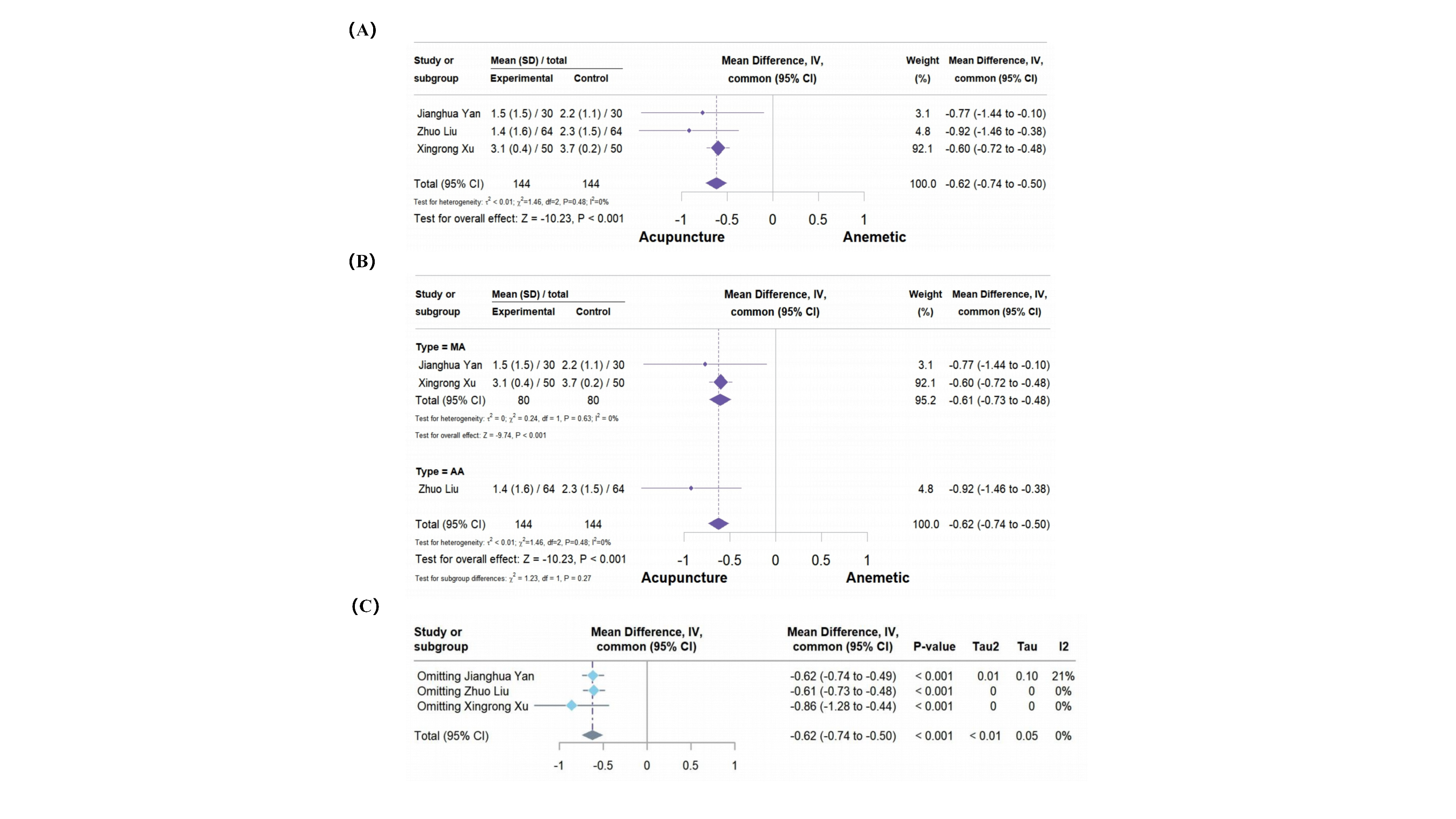


Figure S9 (A) Meta analysis of acute nausea frequency score (B) Subgroup analysis of acute nausea frequency score (C) Sensitive analysis of acute nausea frequency score
