# Supplementary material for: Acupuncture for nausea and vomiting induced by highly emetogenic chemotherapy: a systematic review and meta-analysis
Source: Front Neurol. 2026 Jan 5;16:1692411. doi: 10.3389/fneur.2025.1692411 (PMC12812548; doi:10.3389/fneur.2025.1692411)

Supplementary Material 5

## Supplementary Figures

**Supplementary Figure S1 Subgroup analysis of delay no vomiting events**


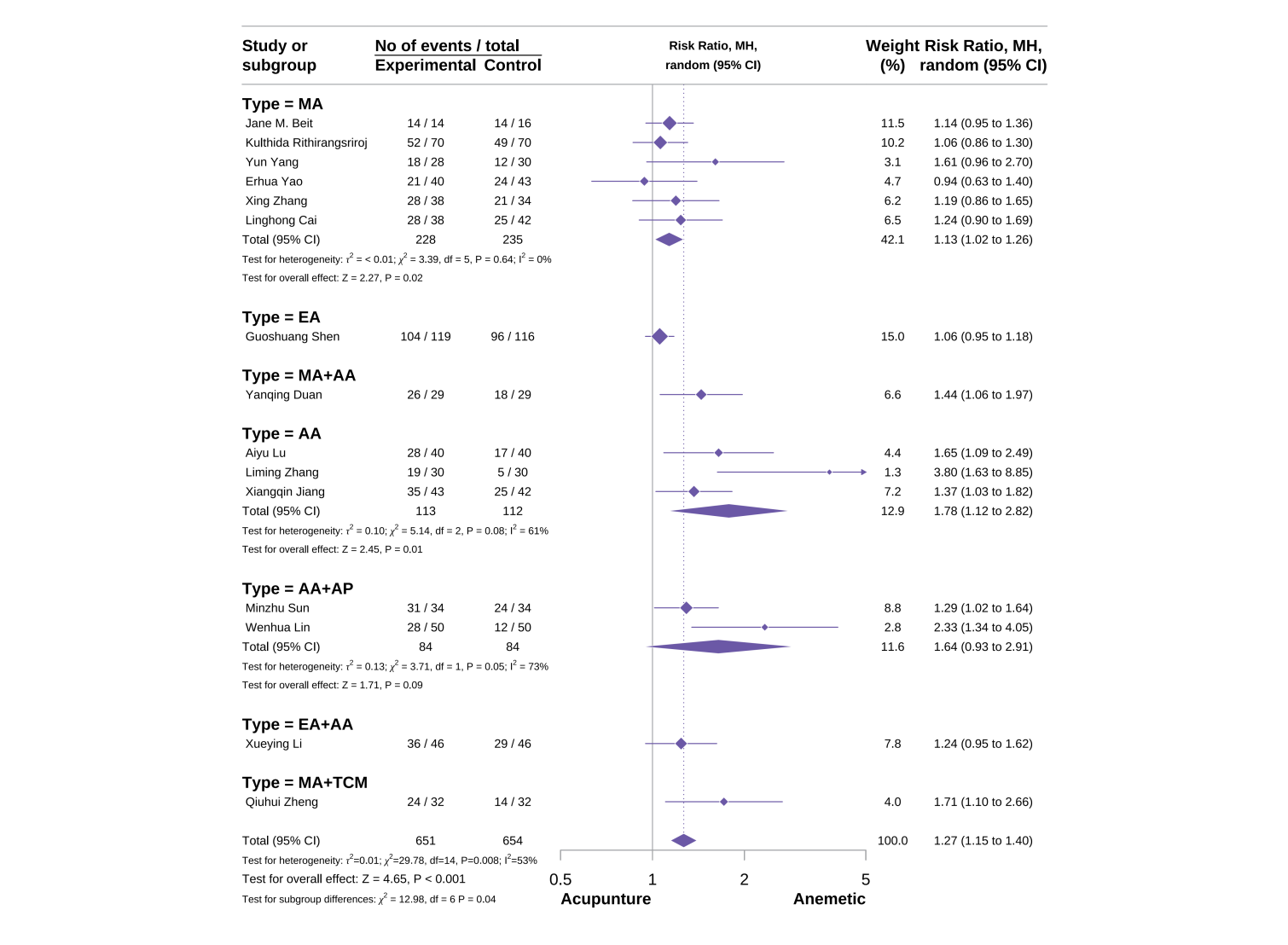


**Supplementary Figure S2 Sensitive analysis and publication bias of delay no vomiting events**


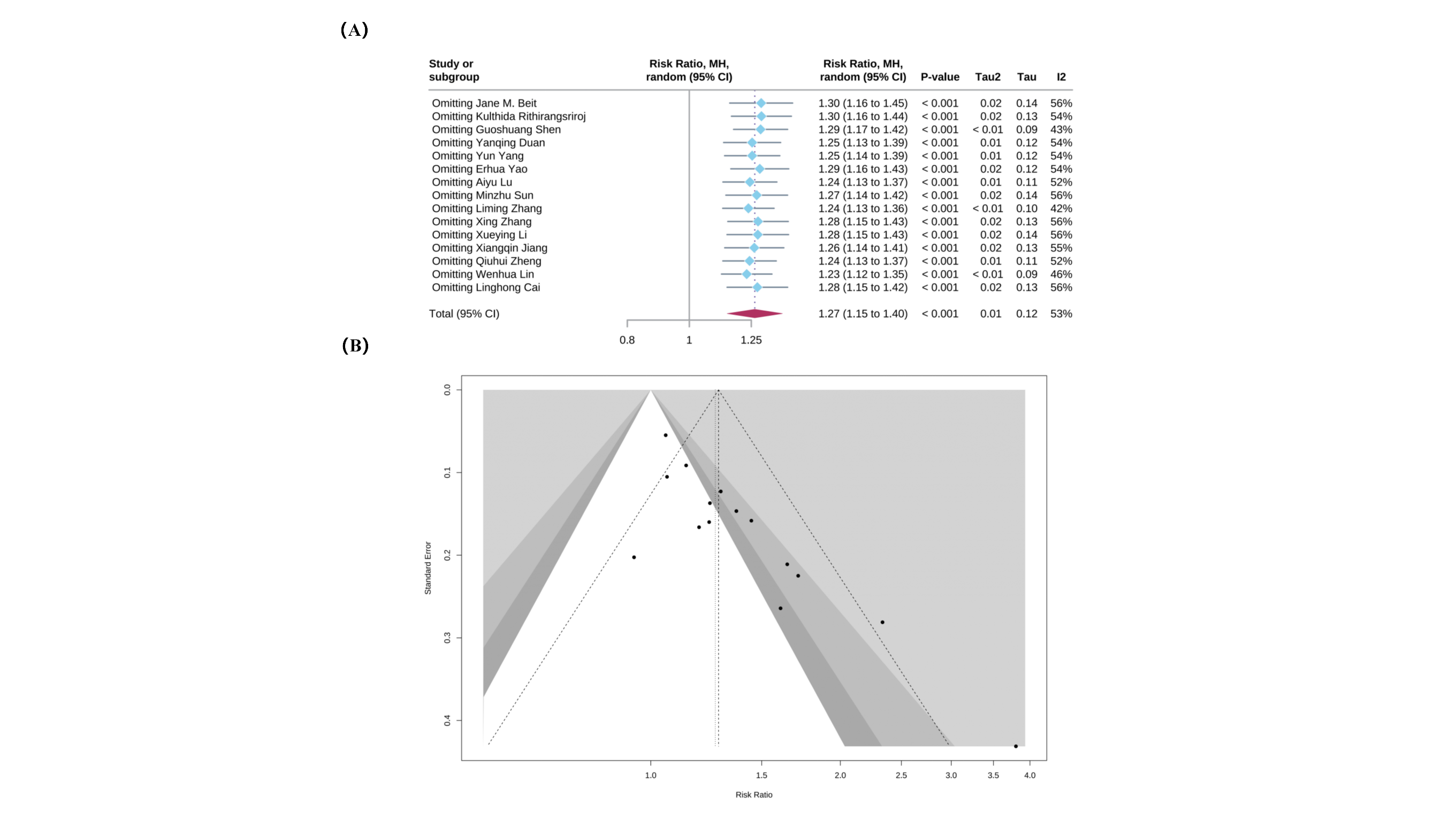


Figure S2 (A) Sensitive analysis of delay no vomiting events (B) publication bias analysis of delay no vomiting events

**Figure S3 Meta analysis of delay nausea severity score**


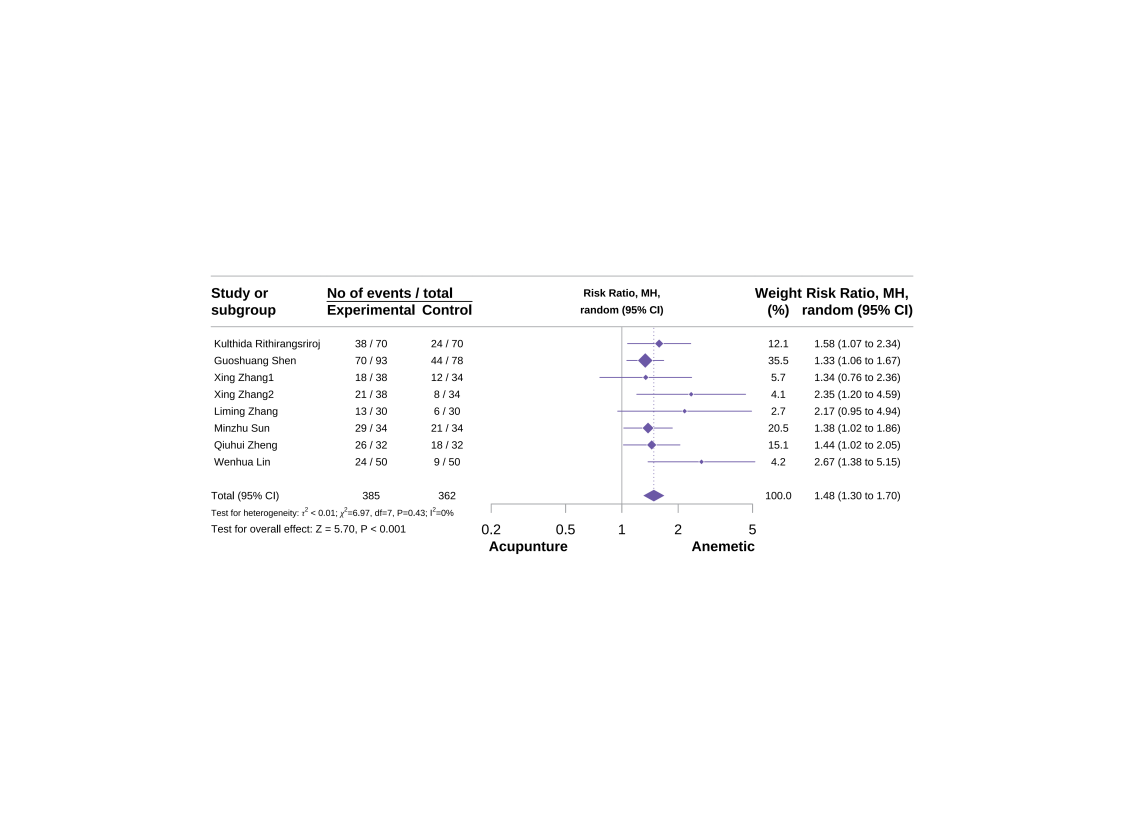


**Figure S4 Subgroup analysis and sensitive analysis of delay no significant nausea events**


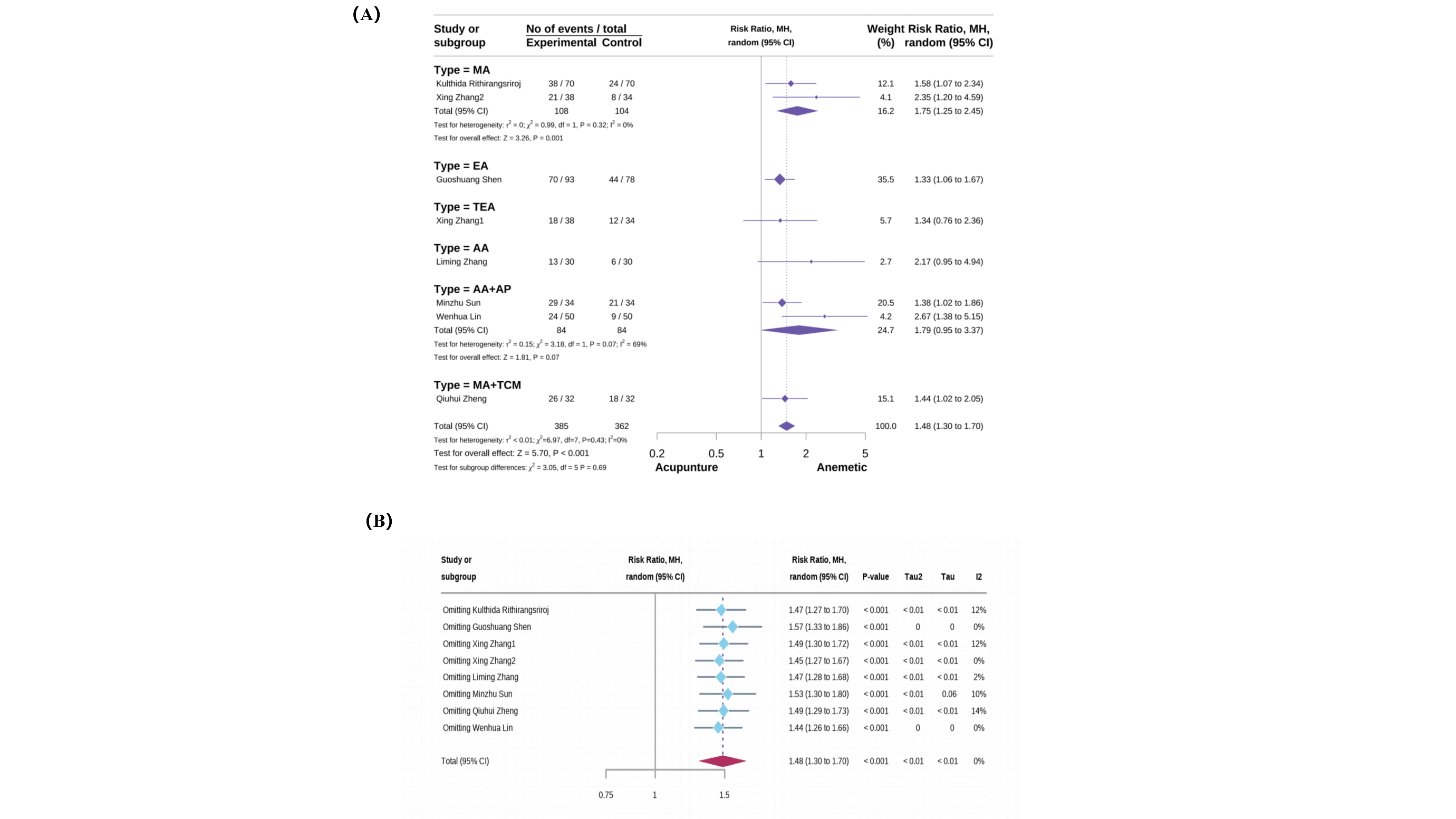


Figure S3 (A) Subgroup analysis of delay no significant nausea events (B) Sensitive analysis of delay no significant nausea events

**Figure S5 Meta analysis, subgroup analysis and sensitive analysis of delay nausea severity score**


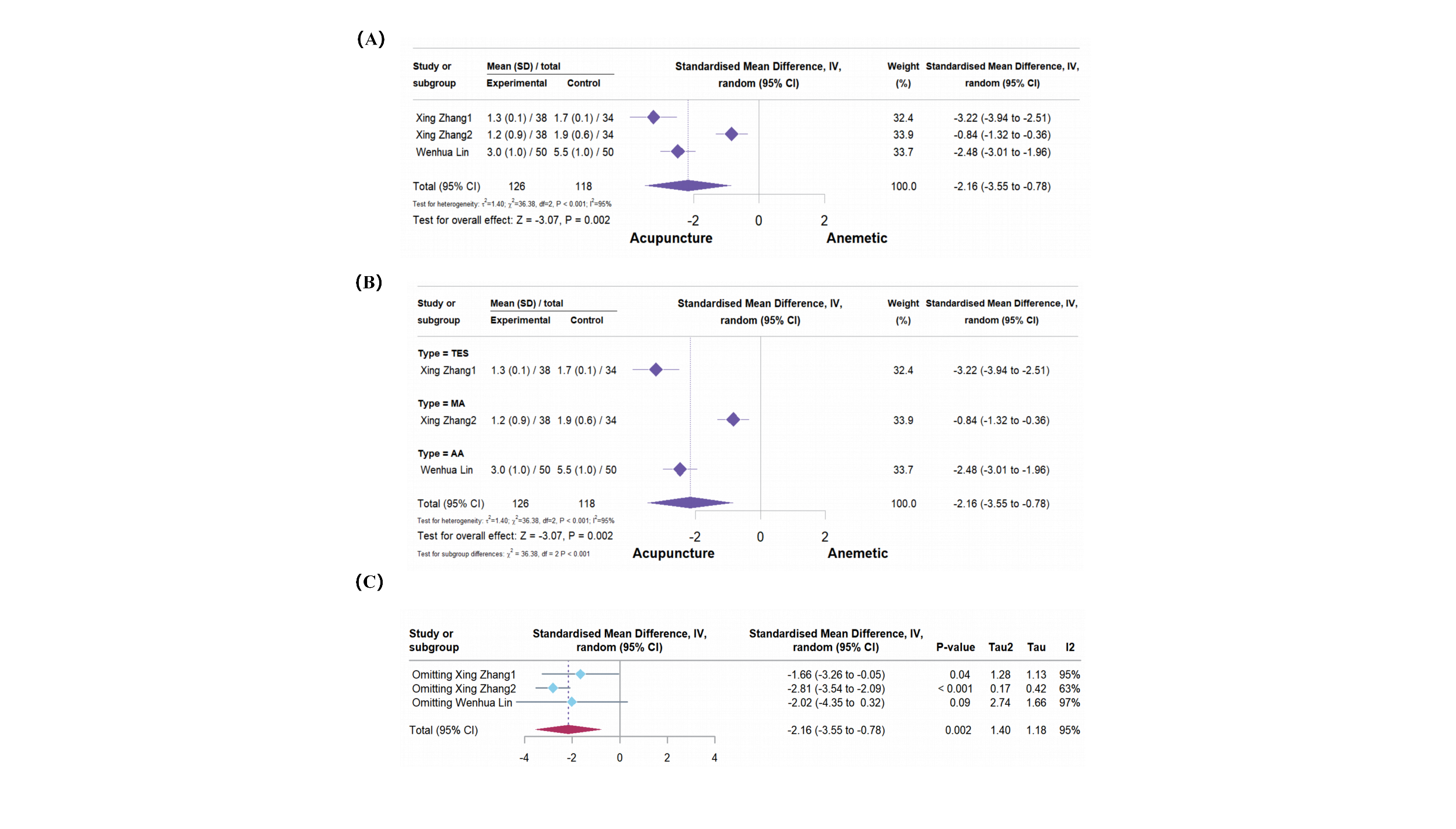


Figure S4 (A) Meta analysis of delay nausea severity score (B) Subgroup analysis of delay nausea severity score (C) Sensitive analysis of delay nausea severity score

**Figure S6 Evidence level assessment**


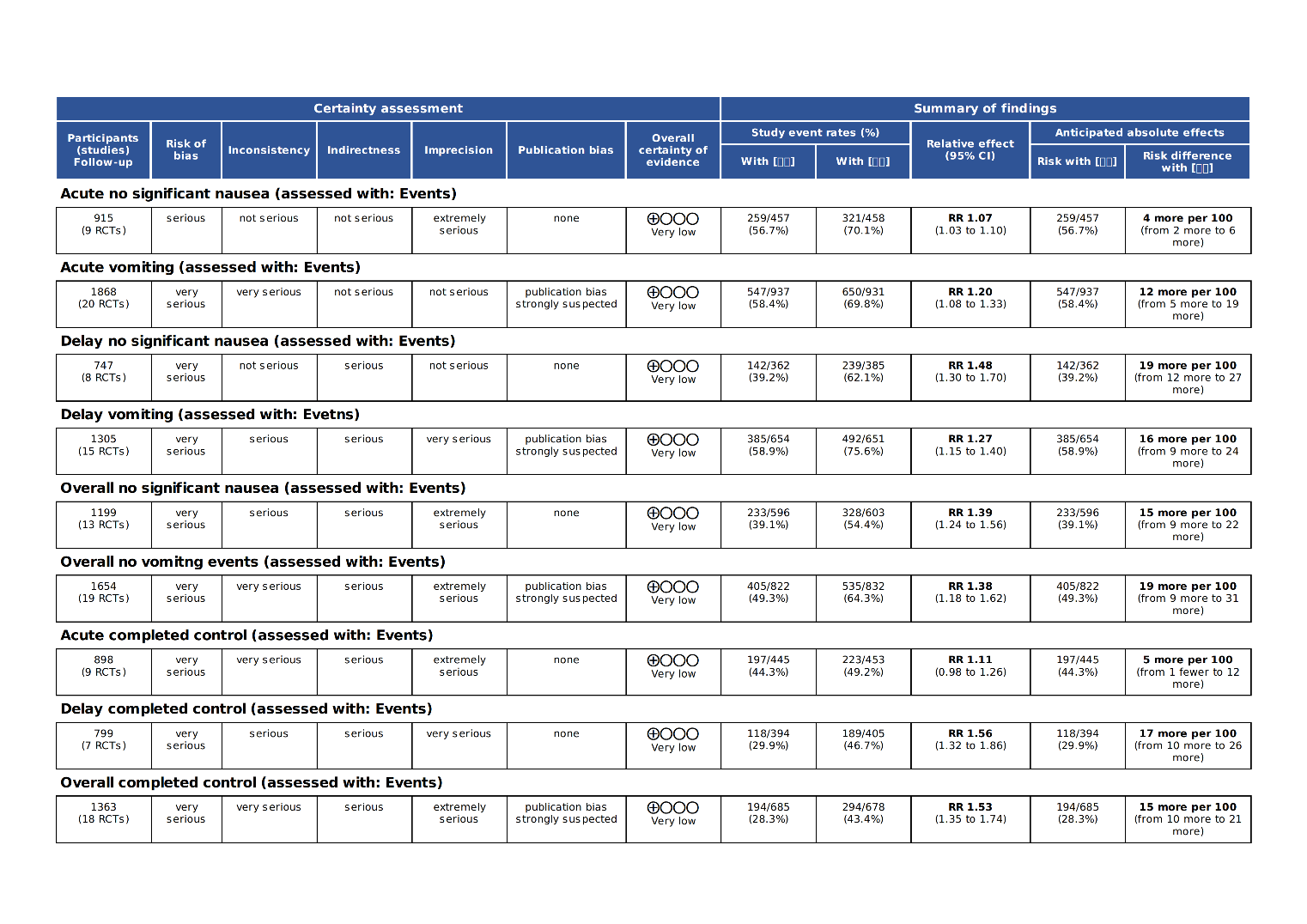


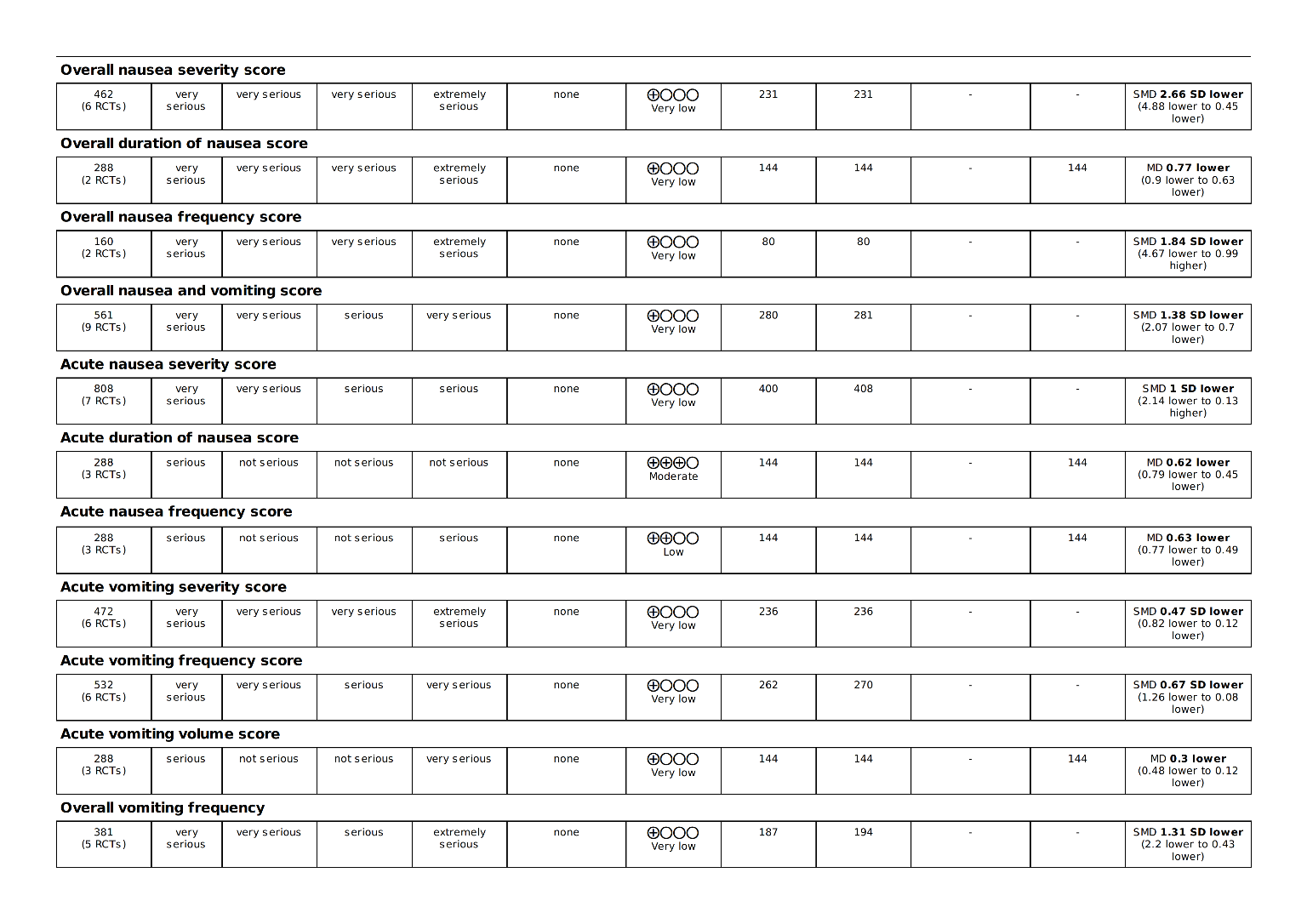


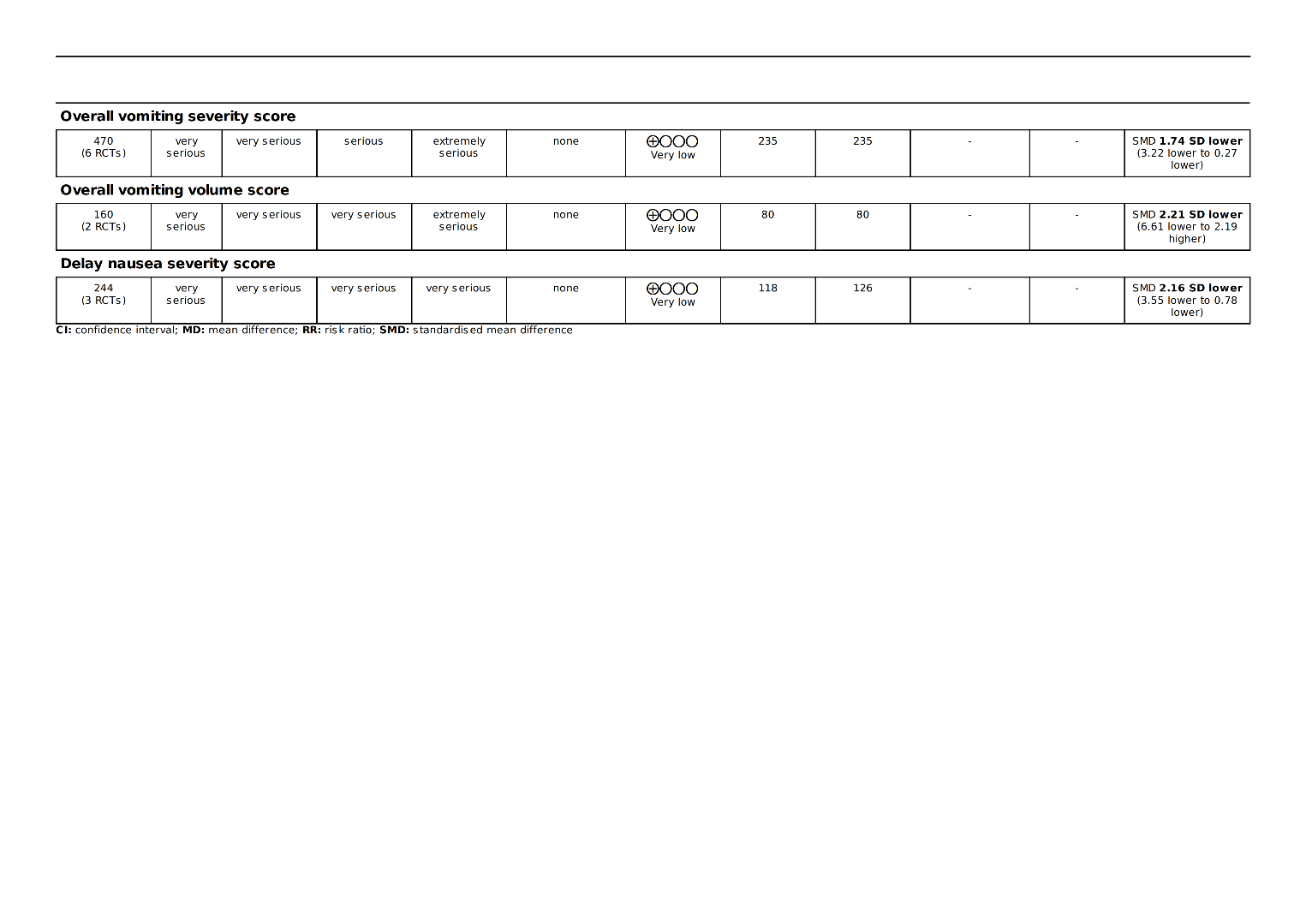

Supplement: Supplementary file 5 [file Table_5.docx]
